# Supplementary material for: Hybridized distance- and contact-based hierarchical structure modeling for folding soluble and membrane proteins
Source: PLoS Comput Biol. 2021 Feb 23;17(2):e1008753. doi: 10.1371/journal.pcbi.1008753 (PMC7935296; doi:10.1371/journal.pcbi.1008753)
Supplement: S1 Table — (DOCX) [file pcbi.1008753.s001.docx]

| **S1 Table.** Target-by-target reconstruction performance on 150 soluble proteins for true C_α_–C_α_ contact maps at various thresholds. | | | | | | | | | | | | | | | | | | | | | | | | | | | |
| --- | --- | --- | --- | --- | --- | --- | --- | --- | --- | --- | --- | --- | --- | --- | --- | --- | --- | --- | --- | --- | --- | --- | --- | --- | --- | --- | --- |
| Target | **8 Å** | | | **8.5 Å** | | | **9 Å** | | | **9.5 Å** | | | **10 Å** | | | **10.5 Å** | | | **11 Å** | | | **11.5 Å** | | | **12 Å** | | |
|  | FT-COMAR | CONFOLD | DConStruct | FT-COMAR | CONFOLD | DConStruct | FT-COMAR | CONFOLD | DConStruct | FT-COMAR | CONFOLD | DConStruct | FT-COMAR | CONFOLD | DConStruct | FT-COMAR | CONFOLD | DConStruct | FT-COMAR | CONFOLD | DConStruct | FT-COMAR | CONFOLD | DConStruct | FT-COMAR | CONFOLD | DConStruct |
| 1a3aA | 0.3212 | 0.8411 | 0.9292 | 0.8245 | 0.8383 | 0.9046 | 0.3303 | 0.8463 | 0.9333 | 0.3425 | 0.865 | 0.9655 | 0.3353 | 0.8921 | 0.9669 | 0.3447 | 0.881 | 0.9613 | 0.3402 | 0.8879 | 0.9721 | 0.3429 | 0.9072 | 0.9712 | 0.9667 | 0.8788 | 0.97 |
| 1a6mA | 0.5619 | 0.8454 | 0.8619 | 0.5882 | 0.904 | 0.9288 | 0.3112 | 0.915 | 0.9369 | 0.7175 | 0.925 | 0.9388 | 0.903 | 0.9138 | 0.9442 | 0.939 | 0.9359 | 0.9698 | 0.9572 | 0.9483 | 0.9706 | 0.9631 | 0.9385 | 0.9741 | 0.358 | 0.9366 | 0.9757 |
| 1a70A | 0.2861 | 0.7759 | 0.843 | 0.7599 | 0.7819 | 0.8181 | 0.801 | 0.8767 | 0.9042 | 0.2909 | 0.8697 | 0.9203 | 0.3073 | 0.8779 | 0.9581 | 0.3013 | 0.8088 | 0.9559 | 0.962 | 0.8829 | 0.9641 | 0.9331 | 0.9088 | 0.9654 | 0.3088 | 0.822 | 0.9549 |
| 1aapA | 0.2937 | 0.7184 | 0.7912 | 0.3087 | 0.7616 | 0.8132 | 0.3315 | 0.7452 | 0.8134 | 0.2993 | 0.7424 | 0.8342 | 0.3629 | 0.7452 | 0.8845 | 0.8061 | 0.792 | 0.8452 | 0.3247 | 0.8021 | 0.8553 | 0.3219 | 0.783 | 0.8659 | 0.3518 | 0.7599 | 0.8162 |
| 1abaA | 0.3167 | 0.7352 | 0.8666 | 0.3002 | 0.7662 | 0.8441 | 0.558 | 0.797 | 0.8849 | 0.3228 | 0.8576 | 0.8714 | 0.8337 | 0.7911 | 0.9109 | 0.7975 | 0.8393 | 0.9086 | 0.346 | 0.8354 | 0.9287 | 0.3637 | 0.8246 | 0.9232 | 0.8966 | 0.8499 | 0.9138 |
| 1ag6A | 0.7028 | 0.8527 | 0.8682 | 0.7219 | 0.7837 | 0.8758 | 0.805 | 0.8096 | 0.9019 | 0.856 | 0.8567 | 0.8911 | 0.8965 | 0.822 | 0.9396 | 0.3203 | 0.8141 | 0.9585 | 0.941 | 0.7961 | 0.9609 | 0.3241 | 0.9092 | 0.9555 | 0.3283 | 0.8393 | 0.9594 |
| 1aoeA | 0.3166 | 0.7831 | 0.8943 | 0.3157 | 0.7803 | 0.9128 | 0.3188 | 0.8481 | 0.9345 | 0.3468 | 0.8489 | 0.9491 | 0.3464 | 0.8859 | 0.9673 | 0.3492 | 0.9167 | 0.9703 | 0.3485 | 0.886 | 0.9773 | 0.9523 | 0.8898 | 0.9812 | 0.3522 | 0.8814 | 0.9731 |
| 1atlA | 0.3242 | 0.9097 | 0.9435 | 0.3329 | 0.8863 | 0.9331 | 0.351 | 0.9075 | 0.9509 | 0.9185 | 0.9391 | 0.9702 | 0.3508 | 0.9281 | 0.9827 | 0.3603 | 0.9429 | 0.9827 | 0.3561 | 0.9477 | 0.9839 | 0.3542 | 0.943 | 0.9855 | 0.355 | 0.9413 | 0.9839 |
| 1atzA | 0.2995 | 0.7568 | 0.819 | 0.2876 | 0.781 | 0.8363 | 0.3182 | 0.79 | 0.8843 | 0.7094 | 0.835 | 0.8925 | 0.7585 | 0.7471 | 0.9183 | 0.7954 | 0.8324 | 0.9333 | 0.8656 | 0.8346 | 0.9328 | 0.838 | 0.8318 | 0.9276 | 0.8734 | 0.8202 | 0.9343 |
| 1avsA | 0.4318 | 0.693 | 0.7493 | 0.5223 | 0.734 | 0.7926 | 0.273 | 0.6514 | 0.7684 | 0.7365 | 0.7907 | 0.8796 | 0.7991 | 0.775 | 0.8845 | 0.3163 | 0.8503 | 0.8873 | 0.8653 | 0.8805 | 0.9432 | 0.8292 | 0.899 | 0.9339 | 0.3213 | 0.8471 | 0.92 |
| 1bdoA | 0.606 | 0.7337 | 0.8448 | 0.2891 | 0.7373 | 0.8313 | 0.2779 | 0.7662 | 0.8696 | 0.7867 | 0.7607 | 0.8983 | 0.8235 | 0.6747 | 0.9166 | 0.8421 | 0.7623 | 0.9133 | 0.8504 | 0.7174 | 0.9317 | 0.8657 | 0.7392 | 0.9446 | 0.9077 | 0.7389 | 0.9378 |
| 1bebA | 0.2926 | 0.7749 | 0.8591 | 0.3125 | 0.8464 | 0.8773 | 0.3195 | 0.8436 | 0.9088 | 0.3234 | 0.8464 | 0.9492 | 0.3208 | 0.8611 | 0.9731 | 0.333 | 0.8245 | 0.976 | 0.3302 | 0.8028 | 0.979 | 0.3275 | 0.8883 | 0.9768 | 0.3335 | 0.8785 | 0.9783 |
| 1behA | 0.7909 | 0.8196 | 0.87 | 0.3186 | 0.8584 | 0.9379 | 0.8991 | 0.8543 | 0.9436 | 0.3485 | 0.8802 | 0.9606 | 0.9491 | 0.8844 | 0.9728 | 0.3449 | 0.8803 | 0.971 | 0.3443 | 0.9006 | 0.9783 | 0.3439 | 0.8854 | 0.9708 | 0.349 | 0.9045 | 0.9799 |
| 1bkrA | 0.5168 | 0.8712 | 0.9018 | 0.3112 | 0.9223 | 0.9293 | 0.307 | 0.8915 | 0.9371 | 0.8439 | 0.906 | 0.9405 | 0.3192 | 0.9171 | 0.9504 | 0.918 | 0.9291 | 0.967 | 0.3188 | 0.9191 | 0.9698 | 0.3221 | 0.9423 | 0.9644 | 0.3233 | 0.942 | 0.9755 |
| 1brfA | 0.515 | 0.6103 | 0.6649 | 0.5728 | 0.6885 | 0.7457 | 0.579 | 0.6816 | 0.2795 | 0.7657 | 0.7325 | 0.788 | 0.7543 | 0.6978 | 0.8561 | 0.3046 | 0.7548 | 0.8562 | 0.3292 | 0.7229 | 0.8811 | 0.7697 | 0.7565 | 0.8539 | 0.3229 | 0.7989 | 0.8811 |
| 1bsgA | 0.8443 | 0.901 | 0.9425 | 0.3557 | 0.9129 | 0.9522 | 0.907 | 0.9419 | 0.9769 | 0.9472 | 0.9355 | 0.9798 | 0.9771 | 0.9575 | 0.9824 | 0.381 | 0.9575 | 0.9877 | 0.3773 | 0.9525 | 0.9836 | 0.3778 | 0.9593 | 0.9897 | 0.9866 | 0.971 | 0.9888 |
| 1c44A | 0.6927 | 0.8009 | 0.7989 | 0.7823 | 0.7826 | 0.8522 | 0.8317 | 0.8512 | 0.8936 | 0.8493 | 0.8479 | 0.8921 | 0.908 | 0.8519 | 0.8987 | 0.8993 | 0.8674 | 0.9077 | 0.937 | 0.8561 | 0.9222 | 0.9252 | 0.871 | 0.9267 | 0.3218 | 0.8491 | 0.9285 |
| 1c52A | 0.2557 | 0.7659 | 0.8481 | 0.2767 | 0.8283 | 0.8964 | 0.7326 | 0.8658 | 0.9018 | 0.8638 | 0.887 | 0.9568 | 0.9287 | 0.8922 | 0.9601 | 0.9152 | 0.872 | 0.9583 | 0.3007 | 0.9028 | 0.9668 | 0.3048 | 0.8494 | 0.9645 | 0.9646 | 0.9062 | 0.9663 |
| 1c9oA | 0.6002 | 0.6976 | 0.7547 | 0.6725 | 0.6926 | 0.734 | 0.2696 | 0.695 | 0.8186 | 0.2823 | 0.7125 | 0.8922 | 0.2858 | 0.7726 | 0.91 | 0.2829 | 0.7287 | 0.9049 | 0.2763 | 0.6466 | 0.8856 | 0.8576 | 0.6862 | 0.9231 | 0.2749 | 0.7542 | 0.914 |
| 1cc8A | 0.2686 | 0.7619 | 0.8234 | 0.3042 | 0.7195 | 0.8354 | 0.6913 | 0.817 | 0.9017 | 0.2898 | 0.8044 | 0.9078 | 0.8229 | 0.8106 | 0.8955 | 0.3268 | 0.843 | 0.9301 | 0.3307 | 0.8346 | 0.9375 | 0.3395 | 0.7471 | 0.9386 | 0.9161 | 0.8459 | 0.9338 |
| 1chdA | 0.3054 | 0.9108 | 0.9367 | 0.3155 | 0.933 | 0.9517 | 0.9061 | 0.9439 | 0.9494 | 0.3287 | 0.9415 | 0.9651 | 0.3218 | 0.9597 | 0.9724 | 0.9618 | 0.9454 | 0.9793 | 0.327 | 0.9521 | 0.9839 | 0.9643 | 0.9601 | 0.984 | 0.983 | 0.9433 | 0.9805 |
| 1cjwA | 0.2835 | 0.8031 | 0.9026 | 0.2979 | 0.783 | 0.9267 | 0.816 | 0.847 | 0.9382 | 0.857 | 0.8074 | 0.9423 | 0.3218 | 0.8274 | 0.9534 | 0.9348 | 0.8585 | 0.9514 | 0.3387 | 0.8103 | 0.9616 | 0.3315 | 0.8493 | 0.9672 | 0.9606 | 0.8744 | 0.9669 |
| 1ckeA | 0.5513 | 0.8502 | 0.8527 | 0.5989 | 0.878 | 0.8409 | 0.7206 | 0.8259 | 0.8636 | 0.7847 | 0.8528 | 0.8766 | 0.831 | 0.8799 | 0.9024 | 0.3576 | 0.8627 | 0.8996 | 0.9312 | 0.8777 | 0.9104 | 0.9251 | 0.8847 | 0.9189 | 0.9516 | 0.8757 | 0.9205 |
| 1ctfA | 0.2767 | 0.6762 | 0.7485 | 0.2923 | 0.7662 | 0.8123 | 0.3009 | 0.7616 | 0.89 | 0.3175 | 0.793 | 0.8825 | 0.9333 | 0.8245 | 0.8947 | 0.8832 | 0.8068 | 0.8944 | 0.3042 | 0.8246 | 0.8967 | 0.3174 | 0.8271 | 0.9171 | 0.3216 | 0.8156 | 0.9284 |
| 1cxyA | 0.2634 | 0.5331 | 0.621 | 0.4244 | 0.6622 | 0.709 | 0.6262 | 0.7332 | 0.7614 | 0.2843 | 0.693 | 0.8354 | 0.3114 | 0.7905 | 0.8975 | 0.2897 | 0.8355 | 0.9073 | 0.3035 | 0.8372 | 0.9384 | 0.301 | 0.7798 | 0.9343 | 0.8883 | 0.7534 | 0.9278 |
| 1cznA | 0.6909 | 0.8855 | 0.9117 | 0.3067 | 0.886 | 0.9267 | 0.3091 | 0.898 | 0.9429 | 0.9095 | 0.9082 | 0.9589 | 0.325 | 0.9416 | 0.9645 | 0.3295 | 0.9224 | 0.9659 | 0.3347 | 0.9433 | 0.9667 | 0.9687 | 0.8884 | 0.97 | 0.3312 | 0.914 | 0.973 |
| 1d0qA | 0.5347 | 0.7866 | 0.8687 | 0.6782 | 0.7739 | 0.8362 | 0.3159 | 0.8453 | 0.8614 | 0.8116 | 0.8154 | 0.8628 | 0.8226 | 0.856 | 0.9195 | 0.8124 | 0.838 | 0.9288 | 0.8602 | 0.8824 | 0.9387 | 0.358 | 0.8842 | 0.9423 | 0.3733 | 0.8685 | 0.9582 |
| 1d1qA | 0.6877 | 0.8569 | 0.9346 | 0.3161 | 0.9062 | 0.9484 | 0.3254 | 0.8667 | 0.9553 | 0.3305 | 0.9072 | 0.9648 | 0.3542 | 0.9072 | 0.9644 | 0.3404 | 0.9055 | 0.9648 | 0.9563 | 0.9168 | 0.9711 | 0.9496 | 0.9104 | 0.978 | 0.9699 | 0.9178 | 0.9702 |
| 1d4oA | 0.7036 | 0.8765 | 0.9251 | 0.763 | 0.913 | 0.9229 | 0.319 | 0.9324 | 0.9524 | 0.9145 | 0.9393 | 0.9673 | 0.32 | 0.9506 | 0.9689 | 0.957 | 0.9402 | 0.9703 | 0.9724 | 0.9363 | 0.9733 | 0.3335 | 0.9303 | 0.9783 | 0.9787 | 0.9462 | 0.9724 |
| 1dbxA | 0.7199 | 0.761 | 0.8705 | 0.3219 | 0.7953 | 0.9371 | 0.3301 | 0.8196 | 0.9308 | 0.9085 | 0.8345 | 0.9434 | 0.9356 | 0.847 | 0.9516 | 0.335 | 0.8213 | 0.9609 | 0.9362 | 0.8421 | 0.9669 | 0.9364 | 0.8216 | 0.9597 | 0.3356 | 0.8256 | 0.9625 |
| 1dixA | 0.7544 | 0.8661 | 0.8856 | 0.8275 | 0.851 | 0.9187 | 0.8881 | 0.8974 | 0.9524 | 0.8899 | 0.9095 | 0.9625 | 0.3538 | 0.904 | 0.9533 | 0.9578 | 0.8956 | 0.9728 | 0.9531 | 0.8944 | 0.9726 | 0.9599 | 0.9115 | 0.9752 | 0.977 | - | 0.9811 |
| 1dlwA | 0.5852 | 0.8894 | 0.8921 | 0.2576 | 0.834 | 0.9076 | 0.7068 | 0.9063 | 0.9303 | 0.2915 | 0.8676 | 0.9419 | 0.3048 | 0.8678 | 0.9526 | 0.3117 | 0.8926 | 0.9598 | 0.3153 | 0.9066 | 0.9726 | 0.3078 | 0.9151 | 0.9628 | 0.3046 | 0.9181 | 0.9605 |
| 1dmgA | 0.2939 | 0.8207 | 0.8789 | 0.7246 | 0.8205 | 0.9091 | 0.3208 | 0.8504 | 0.9121 | 0.3141 | 0.8376 | 0.9226 | 0.8543 | 0.8412 | 0.9283 | 0.886 | 0.8752 | 0.9305 | 0.9058 | 0.862 | 0.934 | 0.9087 | 0.8743 | 0.9325 | 0.3277 | 0.8583 | 0.9396 |
| 1dqgA | 0.293 | 0.8349 | 0.8948 | 0.3067 | 0.837 | 0.9091 | 0.3085 | 0.8653 | 0.9214 | 0.9266 | 0.8294 | 0.9428 | 0.3123 | 0.8932 | 0.9596 | 0.9505 | 0.9012 | 0.9647 | 0.3135 | 0.8895 | 0.967 | 0.3058 | 0.899 | 0.9638 | 0.3139 | 0.8578 | 0.9656 |
| 1dsxA | 0.2788 | 0.7102 | 0.7611 | 0.3092 | 0.8231 | 0.8947 | 0.2925 | 0.7656 | 0.879 | 0.307 | 0.8024 | 0.8999 | 0.8572 | 0.8612 | 0.9462 | 0.8684 | 0.9131 | 0.9561 | 0.906 | 0.9156 | 0.9579 | 0.3173 | 0.9122 | 0.9466 | 0.9523 | 0.8785 | 0.9596 |
| 1eazA | 0.609 | 0.7569 | 0.7492 | 0.6173 | 0.7579 | 0.7534 | 0.2923 | 0.6607 | 0.801 | 0.3062 | 0.7997 | 0.8938 | 0.8568 | 0.8299 | 0.9391 | 0.3162 | 0.8919 | 0.9501 | 0.8795 | 0.8454 | 0.957 | 0.3251 | 0.8984 | 0.9517 | 0.3177 | 0.9159 | 0.9507 |
| 1ej0A | 0.3108 | 0.8777 | 0.9159 | 0.7945 | 0.8828 | 0.9207 | 0.8569 | 0.8818 | 0.9317 | 0.3424 | 0.8877 | 0.9583 | 0.3421 | 0.9182 | 0.9703 | 0.343 | 0.9261 | 0.977 | 0.3412 | 0.9246 | 0.9841 | 0.3477 | 0.9255 | 0.9823 | 0.9726 | 0.9279 | 0.9781 |
| 1ej8A | 0.5991 | 0.7445 | 0.7645 | 0.7553 | 0.7856 | 0.8451 | 0.881 | 0.801 | 0.8605 | 0.882 | 0.7991 | 0.9155 | 0.3471 | 0.8081 | 0.9392 | 0.3449 | 0.86 | 0.9557 | 0.3448 | 0.8035 | 0.9659 | 0.9351 | 0.8539 | 0.9533 | 0.9591 | 0.7818 | 0.9524 |
| 1ek0A | 0.7319 | 0.826 | 0.8963 | 0.3046 | 0.9044 | 0.9267 | 0.3287 | 0.8956 | 0.9482 | 0.878 | 0.8589 | 0.943 | 0.9325 | 0.882 | 0.9414 | 0.9486 | 0.8914 | 0.9516 | 0.3322 | 0.8832 | 0.956 | 0.9475 | 0.917 | 0.9711 | 0.337 | 0.9154 | 0.961 |
| 1f6bA | 0.7372 | 0.7916 | 0.8816 | 0.7482 | 0.8205 | 0.892 | 0.807 | 0.8281 | 0.8924 | 0.3072 | 0.8382 | 0.9178 | 0.3064 | 0.8523 | 0.9334 | 0.9228 | 0.8545 | 0.9412 | 0.9514 | 0.874 | 0.9518 | 0.3148 | 0.8583 | 0.9354 | 0.3112 | 0.8707 | 0.943 |
| 1fcyA | 0.3343 | 0.9352 | 0.9521 | 0.8219 | 0.9384 | 0.9557 | 0.8747 | 0.9396 | 0.9606 | 0.3607 | 0.9433 | 0.9698 | 0.3674 | 0.9504 | 0.9791 | 0.3714 | 0.9389 | 0.9814 | 0.9776 | 0.9498 | 0.9779 | 0.3706 | 0.964 | 0.9859 | 0.3698 | 0.9539 | 0.9853 |
| 1fk5A | 0.5442 | 0.817 | 0.8829 | 0.3049 | 0.8314 | 0.8893 | 0.7097 | 0.7987 | 0.9206 | 0.3089 | 0.7857 | 0.921 | 0.8854 | 0.8322 | 0.9225 | 0.9067 | 0.8104 | 0.9271 | 0.3232 | 0.8337 | 0.9336 | 0.3193 | 0.8424 | 0.9397 | 0.3282 | 0.8599 | 0.9448 |
| 1fl0A | 0.8219 | 0.8483 | 0.8898 | 0.7979 | 0.8609 | 0.9401 | 0.8634 | 0.8507 | 0.9546 | 0.9189 | 0.8786 | 0.9488 | 0.3312 | 0.9046 | 0.9629 | 0.9415 | 0.895 | 0.9674 | 0.9641 | 0.8739 | 0.9714 | 0.327 | 0.8739 | 0.9744 | 0.9715 | 0.8916 | 0.9759 |
| 1fnaA | 0.6113 | 0.7257 | 0.6968 | 0.5883 | 0.7119 | 0.768 | 0.2863 | 0.7069 | 0.7937 | 0.3002 | 0.8032 | 0.8677 | 0.7794 | 0.827 | 0.9108 | 0.8466 | 0.7657 | 0.9278 | 0.8704 | 0.7485 | 0.9244 | 0.8673 | 0.6958 | 0.9363 | 0.8874 | 0.847 | 0.943 |
| 1fqtA | 0.3117 | 0.8463 | 0.8634 | 0.7551 | 0.8351 | 0.8782 | 0.328 | 0.8653 | 0.8737 | 0.327 | 0.878 | 0.3279 | 0.3418 | 0.8324 | 0.9562 | 0.8974 | 0.8668 | 0.9602 | 0.3406 | 0.8685 | 0.9643 | 0.9369 | 0.8745 | 0.9703 | 0.9531 | 0.8669 | 0.9689 |
| 1fvgA | 0.6974 | 0.868 | 0.9271 | 0.3301 | 0.8621 | 0.9356 | 0.3451 | 0.8929 | 0.9435 | 0.8729 | 0.8952 | 0.9539 | 0.3458 | 0.9119 | 0.9571 | 0.3491 | 0.9232 | 0.974 | 0.3471 | 0.93 | 0.976 | 0.3427 | 0.9094 | 0.9781 | 0.9728 | 0.9286 | 0.9791 |
| 1fvkA | 0.6634 | 0.8883 | 0.9132 | 0.7258 | 0.8963 | 0.9152 | 0.3327 | 0.8857 | 0.9342 | 0.8487 | 0.9114 | 0.9579 | 0.8871 | 0.8779 | 0.965 | 0.9302 | 0.9088 | 0.9631 | 0.961 | 0.8747 | 0.9718 | 0.9508 | 0.9282 | 0.9692 | 0.3614 | 0.9308 | 0.9765 |
| 1fx2A | 0.4305 | 0.6404 | 0.8108 | 0.5364 | 0.6708 | 0.6874 | 0.4568 | 0.7184 | 0.7528 | 0.621 | 0.5949 | 0.7408 | 0.3468 | 0.5992 | 0.8282 | 0.6866 | 0.8063 | 0.829 | 0.7154 | 0.6651 | 0.7764 | 0.8181 | 0.7809 | 0.897 | 0.8447 | 0.8206 | 0.9245 |
| 1g2rA | 0.2515 | 0.8432 | 0.8898 | 0.2777 | 0.8119 | 0.8779 | 0.273 | 0.8397 | 0.8812 | 0.2834 | 0.8514 | 0.9044 | 0.8493 | 0.8332 | 0.915 | 0.8493 | 0.8484 | 0.9398 | 0.8638 | 0.8831 | 0.9502 | 0.3287 | 0.9002 | 0.9483 | 0.9193 | 0.8679 | 0.9223 |
| 1g9oA | 0.5924 | 0.6924 | 0.7367 | 0.6021 | 0.6924 | 0.7419 | 0.711 | 0.7457 | 0.8109 | 0.7308 | 0.8269 | 0.8551 | 0.2892 | 0.7995 | 0.8875 | 0.8652 | 0.8184 | 0.9132 | 0.8717 | 0.7797 | 0.9043 | 0.8593 | 0.8036 | 0.9054 | 0.3113 | 0.8094 | 0.9193 |
| 1gbsA | 0.3014 | 0.8708 | 0.9144 | 0.3217 | 0.8882 | 0.9304 | 0.3223 | 0.8886 | 0.9367 | 0.328 | 0.8952 | 0.9597 | 0.9351 | 0.9039 | 0.9547 | 0.3262 | 0.9122 | 0.9544 | 0.9769 | 0.9219 | 0.9626 | 0.3247 | 0.903 | 0.9672 | 0.321 | 0.8913 | 0.9691 |
| 1gmiA | 0.708 | 0.7369 | 0.7947 | 0.7047 | 0.7572 | 0.753 | 0.7908 | 0.7538 | 0.8436 | 0.8179 | 0.7933 | 0.8716 | 0.8631 | 0.8211 | 0.9036 | 0.3263 | 0.7603 | 0.9269 | 0.8981 | 0.8034 | 0.9437 | 0.9163 | 0.8405 | 0.9297 | 0.3472 | 0.8167 | 0.9589 |
| 1gmxA | 0.6057 | 0.7684 | 0.8102 | 0.6367 | 0.7884 | 0.8563 | 0.2872 | 0.889 | 0.8956 | 0.3098 | 0.8665 | 0.9332 | 0.911 | 0.8848 | 0.9414 | 0.916 | 0.8754 | 0.9395 | 0.8901 | 0.8702 | 0.9442 | 0.9198 | 0.893 | 0.9474 | 0.3202 | 0.8828 | 0.9605 |
| 1guuA | 0.2271 | 0.6727 | 0.7671 | 0.4597 | 0.7984 | 0.8317 | 0.234 | 0.8268 | 0.8402 | 0.252 | 0.7452 | 0.8597 | 0.7305 | 0.8034 | 0.8527 | 0.7192 | 0.8759 | 0.881 | 0.8034 | 0.8444 | 0.9027 | 0.8076 | 0.8165 | 0.9191 | 0.8513 | 0.912 | 0.904 |
| 1gz2A | 0.6919 | 0.8144 | 0.819 | 0.7923 | 0.7955 | 0.8858 | 0.3183 | 0.8311 | 0.8951 | 0.8658 | 0.7843 | 0.9367 | 0.8881 | 0.8356 | 0.9518 | 0.8775 | 0.853 | 0.9494 | 0.3302 | 0.8619 | 0.9498 | 0.9381 | 0.8503 | 0.9587 | 0.3275 | 0.7929 | 0.9659 |
| 1gzcA | 0.3204 | 0.8652 | 0.8485 | 0.8264 | 0.8902 | 0.8969 | 0.9357 | 0.8897 | 0.934 | 0.3426 | 0.8993 | 0.9624 | 0.9664 | 0.897 | 0.9685 | 0.9608 | 0.9006 | 0.9782 | 0.3443 | 0.91 | 0.9819 | 0.9696 | 0.9145 | 0.9834 | 0.987 | 0.9235 | 0.9859 |
| 1h0pA | 0.8563 | 0.8673 | 0.9247 | 0.8792 | 0.853 | 0.9484 | 0.3108 | 0.8638 | 0.943 | 0.3228 | 0.8834 | 0.9648 | 0.3196 | 0.8806 | 0.9768 | 0.9583 | 0.8708 | 0.98 | 0.9816 | 0.8942 | 0.981 | 0.9619 | 0.8799 | 0.9792 | 0.319 | 0.8705 | 0.9809 |
| 1h2eA | 0.3303 | 0.9102 | 0.935 | 0.3439 | 0.8598 | 0.9427 | 0.8264 | 0.9171 | 0.9502 | 0.896 | 0.9229 | 0.9621 | 0.9484 | 0.9106 | 0.9784 | 0.9591 | 0.909 | 0.9798 | 0.9603 | 0.9464 | 0.9825 | 0.3645 | 0.9515 | 0.9805 | 0.9822 | 0.9473 | 0.9813 |
| 1h4xA | 0.5257 | 0.7644 | 0.8681 | 0.6896 | 0.7852 | 0.9187 | 0.2934 | 0.7743 | 0.923 | 0.304 | 0.8021 | 0.9468 | 0.314 | 0.7892 | 0.9526 | 0.9308 | 0.7967 | 0.9596 | 0.3198 | 0.8686 | 0.9654 | 0.3231 | 0.8754 | 0.9708 | 0.9635 | 0.872 | 0.9727 |
| 1h98A | 0.6987 | 0.8093 | 0.8439 | 0.2753 | 0.8526 | 0.867 | 0.8017 | 0.8049 | 0.8702 | 0.292 | 0.8156 | 0.9055 | 0.2882 | 0.8251 | 0.93 | 0.9156 | 0.8907 | 0.9415 | 0.921 | 0.83 | 0.9319 | 0.2944 | 0.8649 | 0.948 | 0.9301 | 0.8565 | 0.9352 |
| 1hdoA | 0.3156 | 0.9103 | 0.9401 | 0.3169 | 0.914 | 0.9505 | 0.861 | 0.9317 | 0.9598 | 0.3255 | 0.9063 | 0.9647 | 0.3257 | 0.9509 | 0.9783 | 0.3226 | 0.9405 | 0.9831 | 0.3235 | 0.9531 | 0.979 | 0.3277 | 0.9551 | 0.9781 | 0.9826 | 0.9523 | 0.9842 |
| 1hfcA | 0.3129 | 0.8602 | 0.92 | 0.3087 | 0.8655 | 0.9427 | 0.3028 | 0.8265 | 0.9457 | 0.3174 | 0.8984 | 0.9642 | 0.928 | 0.9055 | 0.9655 | 0.9414 | 0.9103 | 0.9779 | 0.3207 | 0.9126 | 0.9707 | 0.324 | 0.9052 | 0.9702 | 0.3203 | 0.9018 | 0.9752 |
| 1hh8A | 0.3501 | 0.9117 | 0.8321 | 0.3587 | 0.9146 | 0.8744 | 0.3576 | 0.9378 | 0.8248 | 0.3668 | 0.9212 | 0.8748 | 0.3882 | 0.9171 | 0.9431 | 0.4022 | 0.9543 | 0.9582 | 0.3995 | 0.9293 | 0.9706 | 0.399 | 0.9422 | 0.9625 | 0.3959 | 0.9176 | 0.9798 |
| 1htwA | 0.6454 | 0.8699 | 0.8952 | 0.7743 | 0.913 | 0.9291 | 0.2852 | 0.923 | 0.9387 | 0.8495 | 0.9323 | 0.9481 | 0.3141 | 0.9166 | 0.9549 | 0.3197 | 0.9187 | 0.969 | 0.3177 | 0.9493 | 0.9724 | 0.9603 | 0.9469 | 0.9722 | 0.9672 | 0.959 | 0.9779 |
| 1hxnA | 0.3231 | 0.8598 | 0.8291 | 0.3465 | 0.8587 | 0.8763 | 0.839 | 0.8593 | 0.9136 | 0.3415 | 0.8852 | 0.9457 | 0.9323 | 0.8829 | 0.9646 | 0.9355 | 0.8918 | 0.9675 | 0.9706 | 0.8887 | 0.9727 | 0.9642 | 0.9032 | 0.9755 | 0.348 | 0.8932 | 0.9774 |
| 1i1jA | 0.5708 | 0.6794 | 0.5241 | 0.6748 | 0.7039 | 0.5207 | 0.3149 | 0.7464 | 0.5842 | 0.8065 | 0.7476 | 0.3498 | 0.8749 | 0.7712 | 0.921 | 0.3211 | 0.7975 | 0.9034 | 0.8892 | 0.8089 | 0.9207 | 0.3334 | 0.8174 | 0.9241 | 0.3331 | 0.7941 | 0.3249 |
| 1i1nA | 0.8203 | 0.9002 | 0.9457 | 0.8846 | 0.9273 | 0.9517 | 0.3132 | 0.9235 | 0.9645 | 0.321 | 0.937 | 0.9698 | 0.3216 | 0.9183 | 0.9744 | 0.323 | 0.9398 | 0.9811 | 0.9713 | 0.9217 | 0.9851 | 0.3223 | 0.9392 | 0.9847 | 0.3212 | 0.9255 | 0.9873 |
| 1i4jA | 0.6563 | 0.8177 | 0.8429 | 0.3368 | 0.7316 | 0.8557 | 0.6283 | 0.7665 | 0.8739 | 0.3544 | 0.7283 | 0.8639 | 0.3703 | 0.7415 | 0.8747 | 0.8939 | 0.7197 | 0.9208 | 0.8763 | 0.7307 | 0.8949 | 0.863 | 0.7982 | 0.8919 | 0.3602 | 0.7366 | 0.8991 |
| 1i58A | 0.5522 | 0.8415 | 0.8937 | 0.3019 | 0.8562 | 0.888 | 0.6555 | 0.8326 | 0.942 | 0.7681 | 0.9006 | 0.9273 | 0.3499 | 0.909 | 0.9443 | 0.3521 | 0.912 | 0.9641 | 0.9262 | 0.8982 | 0.9561 | 0.3465 | 0.9081 | 0.9764 | 0.3534 | 0.9133 | 0.9589 |
| 1i5gA | 0.2946 | 0.8274 | 0.8977 | 0.2894 | 0.8625 | 0.9221 | 0.782 | 0.8584 | 0.9461 | 0.3106 | 0.9116 | 0.9442 | 0.9411 | 0.8954 | 0.9687 | 0.9541 | 0.9004 | 0.9659 | 0.9642 | 0.899 | 0.9739 | 0.957 | 0.8836 | 0.9756 | 0.3164 | 0.9104 | 0.9731 |
| 1i71A | 0.7079 | 0.7228 | 0.7669 | 0.3361 | 0.6944 | 0.8308 | 0.7302 | 0.6974 | 0.8576 | 0.3388 | 0.7592 | 0.8542 | 0.3551 | 0.7371 | 0.8741 | 0.358 | 0.3145 | 0.9016 | 0.8687 | 0.7471 | 0.8871 | 0.8768 | 0.3413 | 0.9062 | 0.8795 | 0.7412 | 0.9194 |
| 1ihzA | 0.5862 | 0.8491 | 0.8527 | 0.7691 | 0.8246 | 0.88 | 0.3051 | 0.8594 | 0.9032 | 0.788 | 0.8737 | 0.9341 | 0.3371 | 0.8873 | 0.9464 | 0.3331 | 0.8817 | 0.9434 | 0.3312 | 0.8529 | 0.957 | 0.339 | 0.9048 | 0.9668 | 0.9397 | 0.9056 | 0.9624 |
| 1iibA | 0.3012 | 0.8451 | 0.8882 | 0.2935 | 0.8606 | 0.8861 | 0.2971 | 0.8535 | 0.9044 | 0.3132 | 0.8931 | 0.9297 | 0.9218 | 0.9226 | 0.9406 | 0.9394 | 0.9161 | 0.9415 | 0.9557 | 0.9343 | 0.9369 | 0.9462 | 0.9085 | 0.9567 | 0.338 | 0.8918 | 0.9482 |
| 1im5A | 0.3121 | 0.8819 | 0.921 | 0.7905 | 0.88 | 0.9226 | 0.8752 | 0.8821 | 0.96 | 0.3332 | 0.8912 | 0.9604 | 0.3357 | 0.906 | 0.9711 | 0.9373 | 0.9124 | 0.973 | 0.9595 | 0.883 | 0.973 | 0.954 | 0.9141 | 0.9751 | 0.9802 | 0.8936 | 0.9828 |
| 1iwdA | 0.3535 | 0.8997 | 0.9141 | 0.3535 | 0.8557 | 0.9403 | 0.3622 | 0.895 | 0.9546 | 0.9145 | 0.9009 | 0.9666 | 0.9643 | 0.9109 | 0.9744 | 0.954 | 0.9216 | 0.9831 | 0.3607 | 0.9276 | 0.9824 | 0.9615 | 0.9158 | 0.9838 | 0.984 | 0.931 | 0.9865 |
| 1j3aA | 0.2936 | 0.8238 | 0.902 | 0.3032 | 0.8531 | 0.9096 | 0.7467 | 0.8352 | 0.9373 | 0.3217 | 0.8848 | 0.9356 | 0.8483 | 0.8992 | 0.9549 | 0.8937 | 0.9066 | 0.9595 | 0.3275 | 0.8975 | 0.9639 | 0.3249 | 0.8681 | 0.96 | 0.3358 | 0.9249 | 0.959 |
| 1jbeA | 0.2638 | 0.8458 | 0.8892 | 0.2888 | 0.8806 | 0.8996 | 0.2915 | 0.8769 | 0.9334 | 0.8652 | 0.8774 | 0.953 | 0.9179 | 0.9177 | 0.9561 | 0.9304 | 0.9025 | 0.963 | 0.2982 | 0.9159 | 0.9643 | 0.9499 | 0.9144 | 0.956 | 0.9601 | 0.8908 | 0.9659 |
| 1jbkA | 0.2996 | 0.8766 | 0.8978 | 0.7204 | 0.8915 | 0.9141 | 0.3031 | 0.9011 | 0.9563 | 0.8502 | 0.9056 | 0.9578 | 0.3212 | 0.9309 | 0.9703 | 0.9137 | 0.9388 | 0.9763 | 0.3323 | 0.9245 | 0.9708 | 0.9453 | 0.9109 | 0.9746 | 0.3257 | 0.9238 | 0.9696 |
| 1jfuA | 0.3073 | 0.8531 | 0.9086 | 0.3253 | 0.8805 | 0.9293 | 0.3215 | 0.8836 | 0.9488 | 0.9234 | 0.876 | 0.9567 | 0.9631 | 0.8677 | 0.9606 | 0.9512 | 0.9083 | 0.9655 | 0.9723 | 0.9053 | 0.9722 | 0.9692 | 0.9075 | 0.9725 | 0.334 | 0.9356 | 0.9753 |
| 1jfxA | 0.8399 | 0.8402 | 0.9254 | 0.8025 | 0.8723 | 0.9412 | 0.8508 | 0.8924 | 0.9446 | 0.3306 | 0.8983 | 0.9697 | 0.9517 | 0.9268 | 0.9742 | 0.9563 | 0.9279 | 0.9798 | 0.3353 | 0.9297 | 0.9813 | 0.3343 | 0.9202 | 0.9826 | 0.3336 | 0.9216 | 0.9885 |
| 1jkxA | 0.7627 | 0.9053 | 0.9152 | 0.7384 | 0.8677 | 0.9263 | 0.3206 | 0.8956 | 0.9404 | 0.3205 | 0.9003 | 0.9514 | 0.3231 | 0.9064 | 0.9681 | 0.9363 | 0.9125 | 0.9677 | 0.3285 | 0.9321 | 0.9813 | 0.328 | 0.9395 | 0.9758 | 0.9786 | 0.9387 | 0.9827 |
| 1jl1A | 0.59 | 0.7745 | 0.8534 | 0.6631 | 0.8084 | 0.8963 | 0.7092 | 0.8759 | 0.91 | 0.826 | 0.8976 | 0.9283 | 0.8729 | 0.865 | 0.9482 | 0.9005 | 0.8967 | 0.9575 | 0.379 | 0.9016 | 0.9727 | 0.3725 | 0.9213 | 0.9787 | 0.378 | 0.9263 | 0.973 |
| 1jo0A | 0.3312 | 0.8446 | 0.852 | 0.3095 | 0.8075 | 0.9133 | 0.3186 | 0.8373 | 0.9163 | 0.7759 | 0.8493 | 0.9413 | 0.8802 | 0.8641 | 0.9411 | 0.9016 | 0.8331 | 0.965 | 0.3271 | 0.8704 | 0.9688 | 0.3386 | 0.8625 | 0.9676 | 0.3294 | 0.8602 | 0.9539 |
| 1jo8A | 0.2866 | 0.6531 | 0.7114 | 0.2871 | 0.5461 | 0.266 | 0.7371 | 0.6447 | 0.8278 | 0.2828 | 0.731 | 0.8645 | 0.8349 | 0.7274 | 0.8659 | 0.3087 | 0.7974 | 0.8735 | 0.2889 | 0.7719 | 0.8631 | 0.2937 | 0.7976 | 0.8732 | 0.8648 | 0.7316 | 0.9045 |
| 1josA | 0.2847 | 0.7934 | 0.8327 | 0.2852 | 0.8316 | 0.8396 | 0.6635 | 0.83 | 0.8619 | 0.2958 | 0.8515 | 0.8562 | 0.85 | 0.8082 | 0.8822 | 0.3018 | 0.859 | 0.9138 | 0.8913 | 0.8472 | 0.9222 | 0.897 | 0.8508 | 0.9238 | 0.9305 | 0.866 | 0.94 |
| 1jvwA | 0.7399 | 0.8665 | 0.8508 | 0.7487 | 0.7791 | 0.8791 | 0.3491 | 0.8304 | 0.8488 | 0.3515 | 0.8897 | 0.9407 | 0.3781 | 0.8899 | 0.9303 | 0.8848 | 0.8811 | 0.9536 | 0.9065 | 0.872 | 0.9545 | 0.3765 | 0.8975 | 0.9502 | 0.9324 | 0.8599 | 0.9704 |
| 1jwqA | 0.7892 | 0.923 | 0.9601 | 0.8577 | 0.9125 | 0.9629 | 0.3388 | 0.935 | 0.9631 | 0.8927 | 0.9358 | 0.9646 | 0.9444 | 0.9419 | 0.9654 | 0.9581 | 0.9461 | 0.98 | 0.334 | 0.9507 | 0.9785 | 0.9644 | 0.9469 | 0.9801 | 0.9795 | 0.9453 | 0.9822 |
| 1jyhA | 0.298 | 0.6381 | 0.5963 | 0.5041 | 0.6571 | 0.6437 | 0.2843 | 0.7412 | 0.6619 | 0.6531 | 0.7577 | 0.7786 | 0.7915 | 0.8089 | 0.9112 | 0.8463 | 0.8709 | 0.9434 | 0.3215 | 0.8423 | 0.9673 | 0.9131 | 0.8949 | 0.9619 | 0.9423 | 0.8422 | 0.9787 |
| 1k6kA | 0.2998 | 0.8719 | 0.9048 | 0.6068 | 0.8809 | 0.9159 | 0.75 | 0.9025 | 0.9419 | 0.7851 | 0.9304 | 0.9372 | 0.8298 | 0.9427 | 0.9651 | 0.347 | 0.9381 | 0.9621 | 0.9456 | 0.9458 | 0.9712 | 0.3391 | 0.9426 | 0.9772 | 0.3491 | 0.9206 | 0.9754 |
| 1k7cA | 0.3233 | 0.9151 | 0.9259 | 0.8258 | 0.9143 | 0.9447 | 0.8899 | 0.9276 | 0.9518 | 0.3389 | 0.9515 | 0.9696 | 0.3374 | 0.9344 | 0.9713 | 0.3408 | 0.9433 | 0.979 | 0.3402 | 0.951 | 0.9805 | 0.9589 | 0.9482 | 0.9789 | 0.3392 | 0.9604 | 0.978 |
| 1k7jA | 0.316 | 0.8783 | 0.903 | 0.8548 | 0.8857 | 0.9442 | 0.9179 | 0.9182 | 0.9627 | 0.9368 | 0.9122 | 0.9725 | 0.3382 | 0.9289 | 0.9824 | 0.9662 | 0.922 | 0.9811 | 0.9828 | 0.9405 | 0.9864 | 0.9703 | 0.9398 | 0.9849 | 0.34 | 0.9428 | 0.9879 |
| 1kidA | 0.3005 | 0.8731 | 0.8924 | 0.7642 | 0.8716 | 0.9012 | 0.3127 | 0.8341 | 0.9244 | 0.895 | 0.9034 | 0.9415 | 0.336 | 0.8888 | 0.9543 | 0.3349 | 0.9215 | 0.9609 | 0.3194 | 0.9168 | 0.9672 | 0.3188 | 0.9136 | 0.9658 | 0.3277 | 0.9177 | 0.963 |
| 1kq6A | 0.5665 | 0.7554 | 0.8069 | 0.2867 | 0.8143 | 0.8203 | 0.3026 | 0.7918 | 0.86 | 0.632 | 0.8202 | 0.8955 | 0.8703 | 0.8385 | 0.9075 | 0.8449 | 0.8396 | 0.9243 | 0.3289 | 0.8211 | 0.9439 | 0.3356 | 0.8215 | 0.9445 | 0.8547 | 0.864 | 0.9478 |
| 1kqrA | 0.3419 | 0.8414 | 0.8534 | 0.7765 | 0.7713 | 0.8599 | 0.3595 | 0.8584 | 0.9167 | 0.3692 | 0.8436 | 0.939 | 0.3773 | 0.8918 | 0.9605 | 0.3786 | 0.9045 | 0.9625 | 0.3703 | 0.8637 | 0.9654 | 0.3749 | 0.8937 | 0.9719 | 0.3711 | 0.8908 | 0.9695 |
| 1ktgA | 0.2812 | 0.7963 | 0.8614 | 0.7828 | 0.7738 | 0.8516 | 0.325 | 0.8002 | 0.8612 | 0.3333 | 0.8378 | 0.9111 | 0.3591 | 0.8622 | 0.9305 | 0.3565 | 0.8209 | 0.9309 | 0.3431 | 0.8878 | 0.959 | 0.906 | 0.8909 | 0.9531 | 0.9269 | 0.8589 | 0.9465 |
| 1ku3A | 0.282 | 0.6804 | 0.828 | 0.362 | 0.7305 | 0.866 | 0.2912 | 0.7308 | 0.8269 | 0.4235 | 0.6901 | 0.7873 | 0.341 | 0.8312 | 0.8881 | 0.3562 | 0.8321 | 0.8999 | 0.7133 | 0.7634 | 0.8868 | 0.3469 | 0.7963 | 0.9126 | 0.7328 | 0.7955 | 0.9074 |
| 1kw4A | 0.2412 | 0.7539 | 0.8097 | 0.4429 | 0.696 | 0.7885 | 0.5884 | 0.7716 | 0.8229 | 0.2624 | 0.8118 | 0.8867 | 0.2753 | 0.8029 | 0.8796 | 0.304 | 0.8615 | 0.936 | 0.8976 | 0.8547 | 0.9349 | 0.282 | 0.8949 | 0.9498 | 0.2887 | 0.8372 | 0.9408 |
| 1lm4A | 0.3385 | 0.7347 | 0.8608 | 0.7821 | 0.7614 | 0.8959 | 0.8453 | 0.7868 | 0.8812 | 0.3544 | 0.7534 | 0.8173 | 0.8759 | 0.8076 | 0.8248 | 0.3475 | 0.8061 | 0.8143 | 0.3557 | 0.7996 | 0.8209 | 0.9429 | 0.8183 | 0.8352 | 0.9562 | 0.8072 | 0.827 |
| 1lo7A | 0.4452 | 0.6977 | 0.6983 | 0.5273 | 0.7433 | 0.7654 | 0.3058 | 0.8419 | 0.7736 | 0.3328 | 0.7399 | 0.8896 | 0.338 | 0.7887 | 0.9096 | 0.9209 | 0.85 | 0.9285 | 0.3416 | 0.8565 | 0.9318 | 0.9056 | 0.8542 | 0.9297 | 0.9295 | 0.8281 | 0.9166 |
| 1lpyA | 0.4769 | 0.7621 | 0.8057 | 0.5067 | 0.7613 | 0.8794 | 0.3244 | 0.8451 | 0.9365 | 0.695 | 0.8448 | 0.9438 | 0.9238 | 0.8396 | 0.9612 | 0.9215 | 0.8858 | 0.9676 | 0.9315 | 0.8338 | 0.963 | 0.9011 | 0.8316 | 0.9654 | 0.332 | 0.8782 | 0.9693 |
| 1m4jA | 0.3123 | 0.8754 | 0.9193 | 0.7416 | 0.8234 | 0.9256 | 0.33 | 0.8626 | 0.9191 | 0.3353 | 0.8659 | 0.9318 | 0.3431 | 0.8729 | 0.9519 | 0.9352 | 0.9095 | 0.9679 | 0.3462 | 0.8576 | 0.9657 | 0.3477 | 0.8852 | 0.9563 | 0.352 | 0.8875 | 0.9502 |
| 1m8aA | 0.5365 | 0.6745 | 0.7156 | 0.2983 | 0.7366 | 0.7739 | 0.2718 | 0.7651 | 0.8325 | 0.2871 | 0.6954 | 0.8374 | 0.3064 | 0.7839 | 0.8507 | 0.7996 | 0.6653 | 0.8723 | 0.8282 | 0.711 | 0.9257 | 0.7866 | 0.7754 | 0.8932 | 0.8552 | 0.7786 | 0.917 |
| 1mk0A | 0.5954 | 0.8148 | 0.8663 | 0.6709 | 0.8136 | 0.8945 | 0.2948 | 0.8383 | 0.9253 | 0.7719 | 0.8476 | 0.9517 | 0.3258 | 0.8564 | 0.9598 | 0.332 | 0.8732 | 0.9463 | 0.9296 | 0.8839 | 0.9618 | 0.3367 | 0.8752 | 0.9536 | 0.9408 | 0.8941 | 0.959 |
| 1mugA | 0.8158 | 0.8604 | 0.8962 | 0.3031 | 0.8754 | 0.9417 | 0.3086 | 0.8654 | 0.9496 | 0.317 | 0.9045 | 0.9603 | 0.9614 | 0.8973 | 0.9618 | 0.9586 | 0.9174 | 0.9686 | 0.3138 | 0.9181 | 0.9752 | 0.3165 | 0.9247 | 0.9768 | 0.9839 | 0.9254 | 0.976 |
| 1nb9A | 0.6768 | 0.7793 | 0.8545 | 0.3099 | 0.8144 | 0.836 | 0.331 | 0.817 | 0.8631 | 0.3166 | 0.8404 | 0.9186 | 0.8546 | 0.841 | 0.9208 | 0.8778 | 0.8703 | 0.9396 | 0.3305 | 0.8711 | 0.9478 | 0.9319 | 0.8721 | 0.961 | 0.9311 | 0.8737 | 0.9626 |
| 1ne2A | 0.3109 | 0.7826 | 0.8918 | 0.3196 | 0.8556 | 0.9189 | 0.8358 | 0.8252 | 0.8993 | 0.9018 | 0.8292 | 0.8941 | 0.3204 | 0.8577 | 0.9141 | 0.3284 | 0.8618 | 0.9212 | 0.945 | 0.8731 | 0.9329 | 0.9386 | 0.8721 | 0.9496 | 0.3295 | 0.8453 | 0.9377 |
| 1npsA | 0.7943 | 0.7495 | 0.8492 | 0.7831 | 0.7736 | 0.865 | 0.8761 | 0.8126 | 0.8926 | 0.873 | 0.8466 | 0.9315 | 0.905 | 0.8425 | 0.9393 | 0.9101 | 0.8566 | 0.9321 | 0.9263 | 0.8116 | 0.9413 | 0.3148 | 0.8677 | 0.9437 | 0.942 | 0.8195 | 0.9349 |
| 1nrvA | 0.586 | 0.7649 | 0.8045 | 0.6473 | 0.8126 | 0.8611 | 0.7765 | 0.8281 | 0.9259 | 0.3233 | 0.8957 | 0.9282 | 0.3393 | 0.9049 | 0.9541 | 0.9074 | 0.8829 | 0.955 | 0.934 | 0.9206 | 0.9547 | 0.9411 | 0.8947 | 0.9551 | 0.9609 | 0.9078 | 0.956 |
| 1ny1A | 0.7565 | 0.8943 | 0.9374 | 0.3275 | 0.9168 | 0.9526 | 0.3286 | 0.9041 | 0.9661 | 0.8965 | 0.9622 | 0.9808 | 0.3423 | 0.9468 | 0.984 | 0.9595 | 0.9496 | 0.9855 | 0.9735 | 0.9434 | 0.988 | 0.9731 | 0.9516 | 0.9842 | 0.9859 | 0.9417 | 0.9893 |
| 1o1zA | 0.7278 | 0.8606 | 0.9363 | 0.7773 | 0.8842 | 0.9267 | 0.887 | 0.901 | 0.9467 | 0.8988 | 0.9229 | 0.972 | 0.9346 | 0.9187 | 0.9753 | 0.9437 | 0.9382 | 0.9765 | 0.977 | 0.9371 | 0.9814 | 0.9536 | 0.9389 | 0.9841 | 0.3759 | 0.9433 | 0.982 |
| 1p90A | 0.3143 | 0.7535 | 0.777 | 0.3151 | 0.7387 | 0.7809 | 0.7045 | 0.8215 | 0.8474 | 0.3284 | 0.8862 | 0.9178 | 0.9023 | 0.8852 | 0.9611 | 0.3343 | 0.9347 | 0.9746 | 0.9532 | 0.9229 | 0.9711 | 0.9302 | 0.9102 | 0.9594 | 0.3441 | 0.9125 | 0.9721 |
| 1pchA | 0.2759 | 0.9122 | 0.923 | 0.7902 | 0.86 | 0.9126 | 0.8132 | 0.9104 | 0.9313 | 0.3077 | 0.875 | 0.9222 | 0.9199 | 0.9372 | 0.9638 | 0.3114 | 0.8961 | 0.9647 | 0.946 | 0.9132 | 0.9608 | 0.9532 | 0.9309 | 0.9571 | 0.9561 | 0.9302 | 0.9676 |
| 1pkoA | 0.5722 | 0.7137 | 0.7933 | 0.3091 | 0.7094 | 0.8431 | 0.323 | 0.6957 | 0.8551 | 0.3318 | 0.7074 | 0.888 | 0.3254 | 0.7385 | 0.886 | 0.3463 | 0.3329 | 0.901 | 0.3357 | 0.7181 | 0.9048 | 0.8989 | 0.7011 | 0.901 | 0.9193 | 0.7557 | 0.9164 |
| 1qf9A | 0.5329 | 0.8603 | 0.8912 | 0.5947 | 0.8781 | 0.8959 | 0.3108 | 0.9094 | 0.915 | 0.8012 | 0.8941 | 0.9654 | 0.3292 | 0.9397 | 0.9714 | 0.9405 | 0.9436 | 0.9813 | 0.9469 | 0.9296 | 0.9765 | 0.9468 | 0.9359 | 0.9775 | 0.3336 | 0.9463 | 0.9748 |
| 1qjpA | 0.6582 | 0.6729 | 0.7203 | 0.6489 | 0.7653 | 0.7431 | 0.6837 | 0.7394 | 0.7603 | 0.3724 | 0.7726 | 0.8721 | 0.3696 | 0.764 | 0.8645 | 0.8909 | 0.793 | 0.8786 | 0.875 | 0.8108 | 0.8754 | 0.88 | 0.8097 | 0.8721 | 0.8777 | 0.7962 | 0.8757 |
| 1ql0A | 0.3013 | 0.8531 | 0.9323 | 0.3259 | 0.8926 | 0.948 | 0.9111 | 0.9196 | 0.9562 | 0.9254 | 0.9189 | 0.9735 | 0.3262 | 0.9164 | 0.9737 | 0.3315 | 0.9286 | 0.9812 | 0.3281 | 0.9136 | 0.9773 | 0.9683 | 0.9 | 0.9815 | 0.3317 | 0.9174 | 0.9858 |
| 1r26A | 0.525 | 0.6604 | 0.7972 | 0.6983 | 0.7742 | 0.8907 | 0.3115 | 0.8516 | 0.9269 | 0.3307 | 0.8865 | 0.9367 | 0.9159 | 0.8574 | 0.9414 | 0.3499 | 0.877 | 0.9522 | 0.3463 | 0.874 | 0.9674 | 0.938 | 0.8991 | 0.9742 | 0.9533 | 0.8877 | 0.9688 |
| 1roaA | 0.2536 | 0.6711 | 0.5503 | 0.2914 | 0.7109 | 0.7628 | 0.3158 | 0.7455 | 0.8646 | 0.6738 | 0.7777 | 0.8712 | 0.8023 | 0.7709 | 0.8981 | 0.8244 | 0.7902 | 0.9227 | 0.8793 | 0.8391 | 0.9412 | 0.3537 | 0.7914 | 0.9336 | 0.3626 | 0.8229 | 0.9443 |
| 1rw1A | 0.2879 | 0.8361 | 0.8879 | 0.2927 | 0.8522 | 0.8883 | 0.2959 | 0.8776 | 0.9057 | 0.3135 | 0.8625 | 0.9318 | 0.825 | 0.8668 | 0.9667 | 0.3377 | 0.9092 | 0.9583 | 0.9295 | 0.8381 | 0.9549 | 0.9242 | 0.906 | 0.9541 | 0.9054 | 0.8862 | 0.9605 |
| 1rw7A | 0.802 | 0.9079 | 0.9348 | 0.851 | 0.92 | 0.9523 | 0.8844 | 0.9254 | 0.9592 | 0.9133 | 0.9426 | 0.9687 | 0.9653 | 0.9422 | 0.9806 | 0.9623 | 0.9319 | 0.9828 | 0.3356 | 0.9399 | 0.9835 | 0.9689 | 0.9391 | 0.9798 | 0.9822 | 0.9482 | 0.9846 |
| 1rybA | 0.757 | 0.8762 | 0.9158 | 0.3114 | 0.9123 | 0.9312 | 0.3221 | 0.9093 | 0.9334 | 0.9102 | 0.9275 | 0.9479 | 0.9344 | 0.9236 | 0.9506 | 0.3287 | 0.9146 | 0.9642 | 0.9537 | 0.9262 | 0.9599 | 0.9542 | 0.9308 | 0.9704 | 0.9592 | 0.9397 | 0.9693 |
| 1smxA | 0.6009 | 0.6716 | 0.723 | 0.2669 | 0.7042 | 0.7495 | 0.2858 | 0.6929 | 0.7929 | 0.295 | 0.7241 | 0.8375 | 0.3154 | 0.7865 | 0.8903 | 0.7946 | 0.791 | 0.8939 | 0.8107 | 0.7018 | 0.8878 | 0.772 | 0.7471 | 0.8917 | 0.8593 | 0.7278 | 0.8968 |
| 1svyA | 0.2914 | 0.8628 | 0.8256 | 0.6033 | 0.8718 | 0.8714 | 0.3112 | 0.8404 | 0.8769 | 0.8488 | 0.903 | 0.9128 | 0.9112 | 0.8868 | 0.9432 | 0.3547 | 0.8924 | 0.95 | 0.9166 | 0.9013 | 0.9659 | 0.8998 | 0.922 | 0.9476 | 0.357 | 0.8931 | 0.9614 |
| 1t8kA | 0.6391 | 0.8622 | 0.9097 | 0.282 | 0.8732 | 0.8945 | 0.7439 | 0.8844 | 0.9258 | 0.7747 | 0.9162 | 0.9153 | 0.8822 | 0.8603 | 0.956 | 0.3108 | 0.9081 | 0.9611 | 0.3004 | 0.9066 | 0.9555 | 0.2945 | 0.9306 | 0.9564 | 0.9407 | 0.9191 | 0.9583 |
| 1tifA | 0.2657 | 0.541 | 0.7439 | 0.2644 | 0.5747 | 0.647 | 0.5799 | 0.6696 | 0.7602 | 0.6384 | 0.7264 | 0.7615 | 0.7226 | 0.6464 | 0.8075 | 0.7209 | 0.6944 | 0.7806 | 0.3295 | 0.6515 | 0.7546 | 0.7889 | 0.7317 | 0.8214 | 0.3463 | 0.8178 | 0.8501 |
| 1tqgA | 0.3505 | 0.8971 | 0.9193 | 0.4725 | 0.9115 | 0.9237 | 0.2883 | 0.9457 | 0.9393 | 0.3323 | 0.9623 | 0.9628 | 0.9311 | 0.9084 | 0.9674 | 0.3604 | 0.959 | 0.9728 | 0.3569 | 0.9485 | 0.9684 | 0.3602 | 0.9481 | 0.9606 | 0.944 | 0.9619 | 0.9695 |
| 1tqhA | 0.7313 | 0.9242 | 0.9394 | 0.8079 | 0.8912 | 0.91 | 0.3435 | 0.9222 | 0.9546 | 0.8963 | 0.95 | 0.9804 | 0.9519 | 0.9601 | 0.9766 | 0.3545 | 0.963 | 0.9788 | 0.3492 | 0.9475 | 0.9739 | 0.9529 | 0.9522 | 0.9847 | 0.978 | 0.9479 | 0.9887 |
| 1tzvA | 0.633 | 0.8796 | 0.9393 | 0.2848 | 0.9175 | 0.9455 | 0.6634 | 0.8943 | 0.953 | 0.7906 | 0.91 | 0.9501 | 0.9119 | 0.9336 | 0.9539 | 0.3321 | 0.949 | 0.9772 | 0.3392 | 0.921 | 0.9843 | 0.331 | 0.946 | 0.9754 | 0.9447 | 0.9241 | 0.9709 |
| 1vfyA | 0.4297 | 0.6093 | 0.6611 | 0.4561 | 0.6362 | 0.6914 | 0.2889 | 0.5891 | 0.837 | 0.2981 | 0.5693 | 0.8463 | 0.3086 | 0.5895 | 0.8379 | 0.6933 | 0.6761 | 0.7832 | 0.3209 | 0.6747 | 0.8611 | 0.308 | 0.6719 | 0.8514 | 0.3176 | 0.6925 | 0.8707 |
| 1vhuA | 0.7624 | 0.9166 | 0.9351 | 0.8279 | 0.9232 | 0.9513 | 0.32 | 0.9449 | 0.9699 | 0.9239 | 0.9439 | 0.9692 | 0.3242 | 0.9526 | 0.9806 | 0.3232 | 0.9538 | 0.9815 | 0.3235 | 0.9574 | 0.9827 | 0.9687 | 0.963 | 0.9814 | 0.3188 | 0.9543 | 0.9834 |
| 1vjkA | 0.6128 | 0.817 | 0.7607 | 0.6642 | 0.8693 | 0.8633 | 0.7396 | 0.7858 | 0.8955 | 0.3274 | 0.8789 | 0.9073 | 0.9025 | 0.8707 | 0.9488 | 0.3187 | 0.9192 | 0.9496 | 0.9482 | 0.9044 | 0.9623 | 0.3249 | 0.8845 | 0.9469 | 0.9548 | 0.8968 | 0.9527 |
| 1vmbA | 0.6072 | 0.7352 | 0.7262 | 0.6735 | 0.7646 | 0.7967 | 0.2894 | 0.7649 | 0.8193 | 0.3204 | 0.7865 | 0.8603 | 0.3218 | 0.6898 | 0.9024 | 0.86 | 0.807 | 0.9099 | 0.3301 | 0.7599 | 0.9163 | 0.8616 | 0.7813 | 0.9104 | 0.3541 | 0.8132 | 0.9323 |
| 1vp6A | 0.6077 | 0.8279 | 0.8417 | 0.2991 | 0.819 | 0.8563 | 0.3129 | 0.8383 | 0.9215 | 0.3068 | 0.8595 | 0.9432 | 0.8961 | 0.8963 | 0.9562 | 0.9339 | 0.8901 | 0.966 | 0.9363 | 0.8887 | 0.9754 | 0.3296 | 0.8883 | 0.97 | 0.3303 | 0.9023 | 0.9706 |
| 1w0hA | 0.2834 | 0.8459 | 0.9182 | 0.6884 | 0.8328 | 0.9248 | 0.8069 | 0.8849 | 0.9436 | 0.8325 | 0.9135 | 0.9582 | 0.9399 | 0.9261 | 0.9755 | 0.3536 | 0.8979 | 0.9762 | 0.3608 | 0.9088 | 0.9807 | 0.3539 | 0.8963 | 0.9799 | 0.3541 | 0.9162 | 0.9859 |
| 1whiA | 0.6222 | 0.7686 | 0.8415 | 0.7897 | 0.7321 | 0.8737 | 0.3156 | 0.7808 | 0.8947 | 0.8638 | 0.8188 | 0.9224 | 0.3328 | 0.8264 | 0.9289 | 0.9007 | 0.8565 | 0.9417 | 0.9149 | 0.8441 | 0.9428 | 0.9209 | 0.824 | 0.9491 | 0.3439 | 0.8493 | 0.9423 |
| 1wjxA | 0.5335 | 0.7606 | 0.3196 | 0.2953 | 0.8017 | 0.8251 | 0.2907 | 0.7281 | 0.8991 | 0.8531 | 0.806 | 0.8864 | 0.8463 | 0.8227 | 0.9422 | 0.33 | 0.7963 | 0.9542 | 0.8962 | 0.8287 | 0.9457 | 0.9223 | 0.8417 | 0.9439 | 0.3285 | 0.8341 | 0.9539 |
| 1wkcA | 0.2882 | 0.8941 | 0.8884 | 0.3034 | 0.8718 | 0.9024 | 0.3069 | 0.8556 | 0.9348 | 0.8473 | 0.8884 | 0.9323 | 0.9333 | 0.8901 | 0.9519 | 0.9336 | 0.8888 | 0.9551 | 0.3218 | 0.8911 | 0.9663 | 0.9452 | 0.9033 | 0.9745 | 0.3241 | 0.8934 | 0.9677 |
| 1xdzA | 0.7519 | 0.8507 | 0.8974 | 0.3211 | 0.9279 | 0.9441 | 0.3232 | 0.9267 | 0.9445 | 0.3346 | 0.9206 | 0.9721 | 0.3336 | 0.9477 | 0.9834 | 0.3384 | 0.9515 | 0.9791 | 0.3403 | 0.9389 | 0.9855 | 0.9671 | 0.9473 | 0.9868 | 0.3355 | 0.9207 | 0.9849 |
| 1xffA | 0.8334 | 0.9271 | 0.9377 | 0.3418 | 0.9309 | 0.9603 | 0.3404 | 0.9581 | 0.9707 | 0.3459 | 0.9587 | 0.9765 | 0.3436 | 0.9622 | 0.9846 | 0.3475 | 0.9598 | 0.9856 | 0.344 | 0.9578 | 0.9846 | 0.9716 | 0.955 | 0.9886 | 0.9885 | 0.9525 | 0.9869 |
| 1xkrA | 0.6824 | 0.8724 | 0.8829 | 0.7604 | 0.8889 | 0.9193 | 0.8225 | 0.9118 | 0.9406 | 0.8414 | 0.9262 | 0.9677 | 0.3511 | 0.9516 | 0.9728 | 0.9317 | 0.9554 | 0.9764 | 0.3547 | 0.9396 | 0.981 | 0.9506 | 0.9637 | 0.9796 | 0.979 | 0.9546 | 0.9841 |
| 2arcA | 0.2975 | 0.8249 | 0.9116 | 0.2991 | 0.8654 | 0.9339 | 0.3014 | 0.7851 | 0.932 | 0.3152 | 0.8744 | 0.9517 | 0.9362 | 0.8727 | 0.9672 | 0.9444 | 0.8913 | 0.9693 | 0.3255 | 0.8767 | 0.9674 | 0.9487 | 0.9119 | 0.9752 | 0.3344 | 0.9247 | 0.978 |
| 2cuaA | 0.8022 | 0.7302 | 0.857 | 0.3004 | 0.8122 | 0.8836 | 0.3164 | 0.8001 | 0.8869 | 0.8747 | 0.8004 | 0.9282 | 0.8766 | 0.8118 | 0.9531 | 0.9009 | 0.8004 | 0.9547 | 0.9412 | 0.8803 | 0.9663 | 0.9424 | 0.8054 | 0.9622 | 0.958 | 0.9117 | 0.9618 |
| 2hs1A | 0.2773 | 0.6755 | 0.6549 | 0.6446 | 0.7078 | 0.7194 | 0.6848 | 0.6472 | 0.7285 | 0.7856 | 0.7902 | 0.8861 | 0.322 | 0.8094 | 0.901 | 0.8608 | 0.8174 | 0.9314 | 0.3296 | 0.8275 | 0.9276 | 0.3201 | 0.7694 | 0.9522 | 0.949 | 0.8371 | 0.9523 |
| 2mhrA | 0.3766 | 0.8788 | 0.919 | 0.5819 | 0.8932 | 0.9485 | 0.6326 | 0.8909 | 0.9513 | 0.8059 | 0.8794 | 0.9664 | 0.9406 | 0.9289 | 0.9628 | 0.3376 | 0.9406 | 0.9646 | 0.3468 | 0.952 | 0.972 | 0.3416 | 0.9238 | 0.9776 | 0.962 | 0.9097 | 0.9748 |
| 2phyA | 0.2843 | 0.794 | 0.8309 | 0.2852 | 0.8538 | 0.8969 | 0.2916 | 0.8144 | 0.9027 | 0.8518 | 0.8917 | 0.9361 | 0.9231 | 0.8789 | 0.9438 | 0.9105 | 0.9208 | 0.9658 | 0.9596 | 0.8977 | 0.9663 | 0.954 | 0.8893 | 0.9719 | 0.9636 | 0.921 | 0.9725 |
| 2tpsA | 0.8409 | 0.9432 | 0.9579 | 0.8715 | 0.9402 | 0.956 | 0.9046 | 0.9413 | 0.9744 | 0.3392 | 0.9456 | 0.9777 | 0.3339 | 0.9654 | 0.9817 | 0.3415 | 0.9586 | 0.9877 | 0.9822 | 0.9429 | 0.9841 | 0.3427 | 0.9655 | 0.9886 | 0.3394 | 0.9688 | 0.988 |
| 2vxnA | 0.3271 | 0.9024 | 0.9104 | 0.3255 | 0.9355 | 0.9559 | 0.8784 | 0.9454 | 0.969 | 0.3321 | 0.9376 | 0.9774 | 0.3271 | 0.956 | 0.981 | 0.9546 | 0.9532 | 0.9847 | 0.972 | 0.9583 | 0.9869 | 0.3349 | 0.9643 | 0.9861 | 0.3288 | 0.9645 | 0.9863 |
| 3borA | 0.2891 | 0.889 | 0.9202 | 0.7854 | 0.8947 | 0.9426 | 0.3169 | 0.9097 | 0.9488 | 0.326 | 0.8985 | 0.9592 | 0.933 | 0.9383 | 0.9615 | 0.9372 | 0.9318 | 0.9583 | 0.9516 | 0.926 | 0.9622 | 0.3386 | 0.9393 | 0.9661 | 0.3353 | 0.9333 | 0.9638 |
| 3dqgA | 0.3311 | 0.561 | 0.6975 | 0.3334 | 0.669 | 0.7386 | 0.3564 | 0.6033 | 0.8093 | 0.3643 | 0.7108 | 0.8346 | 0.3723 | 0.6662 | 0.907 | 0.3941 | 0.649 | 0.9176 | 0.3893 | 0.6359 | 0.916 | 0.3857 | 0.8088 | 0.95 | 0.3898 | 0.793 | 0.9219 |
| 5ptpA | 0.8649 | 0.8669 | 0.931 | 0.3579 | 0.8716 | 0.9544 | 0.3533 | 0.8961 | 0.9618 | 0.9362 | 0.8785 | 0.9718 | 0.3553 | 0.9137 | 0.9788 | 0.9599 | 0.9061 | 0.9807 | 0.355 | 0.8816 | 0.9798 | 0.9665 | 0.9071 | 0.98 | 0.9835 | 0.8977 | 0.9823 |
|  |  |  |  |  |  |  |  |  |  |  |  |  |  |  |  |  |  |  |  |  |  |  |  |  |  |  |  |
| Mean | 0.49 | 0.81 | 0.85 | 0.51 | 0.82 | 0.87 | 0.51 | 0.84 | 0.90 | 0.57 | 0.85 | 0.91 | 0.62 | 0.86 | 0.94 | 0.65 | 0.87 | 0.95 | 0.62 | 0.87 | 0.95 | 0.65 | 0.88 | 0.95 | 0.63 | 0.88 | 0.95 |
| Median | 0.52 | 0.83 | 0.87 | 0.46 | 0.84 | 0.90 | 0.33 | 0.85 | 0.92 | 0.37 | 0.87 | 0.94 | 0.76 | 0.88 | 0.95 | 0.81 | 0.89 | 0.96 | 0.38 | 0.88 | 0.97 | 0.82 | 0.90 | 0.96 | 0.38 | 0.89 | 0.97 |
