# Supplementary material for: Hybridized distance- and contact-based hierarchical structure modeling for folding soluble and membrane proteins
Source: PLoS Comput Biol. 2021 Feb 23;17(2):e1008753. doi: 10.1371/journal.pcbi.1008753 (PMC7935296; doi:10.1371/journal.pcbi.1008753)
Supplement: S2 Table — (DOCX) [file pcbi.1008753.s002.docx]

| **S2 Table.** Target-by-target reconstruction performance on 150 soluble proteins for true C_β_–C_β_ contact maps at various thresholds. | | | | | | | | | | | | | | | | | | |
| --- | --- | --- | --- | --- | --- | --- | --- | --- | --- | --- | --- | --- | --- | --- | --- | --- | --- | --- |
| Target | **8 Å** | | **8.5 Å** | | **9 Å** | | **9.5 Å** | | **10 Å** | | **10.5 Å** | | **11 Å** | | **11.5 Å** | | **12 Å** | |
|  | CONFOLD | DConStruct | CONFOLD | DConStruct | CONFOLD | DConStruct | CONFOLD | DConStruct | CONFOLD | DConStruct | CONFOLD | DConStruct | CONFOLD | DConStruct | CONFOLD | DConStruct | CONFOLD | DConStruct |
| 1a3aA | 0.8825 | 0.9127 | 0.9001 | 0.9107 | 0.8827 | 0.9364 | 0.8936 | 0.9391 | 0.8921 | 0.9486 | 0.9143 | 0.9435 | 0.9188 | 0.9511 | 0.9173 | 0.9435 | 0.9242 | 0.946 |
| 1a6mA | 0.9026 | 0.8707 | 0.9105 | 0.8721 | 0.9254 | 0.8829 | 0.9217 | 0.8955 | 0.9369 | 0.9103 | 0.9429 | 0.9302 | 0.9425 | 0.9279 | 0.948 | 0.9291 | 0.9503 | 0.9357 |
| 1a70A | 0.8531 | 0.8698 | 0.8717 | 0.8795 | 0.9137 | 0.8968 | 0.9152 | 0.9043 | 0.8976 | 0.9258 | 0.93 | 0.9155 | 0.9305 | 0.9263 | 0.9268 | 0.9306 | 0.9219 | 0.9311 |
| 1aapA | 0.693 | 0.8124 | 0.6657 | 0.761 | 0.8189 | 0.8427 | 0.8387 | 0.8309 | 0.8094 | 0.7888 | 0.7997 | 0.8315 | 0.8048 | 0.8244 | 0.7985 | 0.8119 | 0.7759 | 0.8055 |
| 1abaA | 0.8352 | 0.8567 | 0.8502 | 0.8844 | 0.8498 | 0.8661 | 0.8454 | 0.8198 | 0.9073 | 0.8571 | 0.8497 | 0.8738 | 0.8959 | 0.8784 | 0.9159 | 0.8988 | 0.8743 | 0.8834 |
| 1ag6A | 0.8397 | 0.8947 | 0.9043 | 0.9085 | 0.8558 | 0.902 | 0.9222 | 0.9183 | 0.9183 | 0.93 | 0.9195 | 0.9124 | 0.9365 | 0.936 | 0.9287 | 0.9309 | 0.9289 | 0.9331 |
| 1aoeA | 0.909 | 0.9163 | 0.8958 | 0.9463 | 0.9211 | 0.9433 | 0.9311 | 0.9378 | 0.919 | 0.9529 | 0.9325 | 0.9577 | 0.8871 | 0.959 | 0.8608 | 0.9574 | 0.9275 | 0.9638 |
| 1atlA | 0.9213 | 0.9436 | 0.9186 | 0.941 | 0.9286 | 0.9514 | 0.9418 | 0.9563 | 0.9415 | 0.9643 | 0.9569 | 0.9637 | 0.9277 | 0.9622 | 0.9429 | 0.9605 | 0.9389 | 0.9676 |
| 1atzA | 0.8048 | 0.7988 | 0.799 | 0.8384 | 0.8083 | 0.8317 | 0.7703 | 0.8761 | 0.8618 | 0.8693 | 0.8812 | 0.9137 | 0.8741 | 0.891 | 0.8497 | 0.9043 | 0.8475 | 0.9135 |
| 1avsA | 0.8387 | 0.887 | 0.8373 | 0.8944 | 0.903 | 0.8558 | 0.874 | 0.8415 | 0.9059 | 0.8775 | 0.8972 | 0.8694 | 0.8921 | 0.8763 | 0.8945 | 0.8446 | 0.913 | 0.8584 |
| 1bdoA | 0.7247 | 0.8398 | 0.7373 | 0.8414 | 0.7361 | 0.8476 | 0.2968 | 0.8686 | 0.794 | 0.9036 | 0.7939 | 0.9021 | 0.7994 | 0.9236 | 0.307 | 0.9097 | 0.2983 | 0.9176 |
| 1bebA | 0.8714 | 0.932 | 0.8815 | 0.9323 | 0.8818 | 0.9413 | 0.862 | 0.9478 | 0.8883 | 0.9563 | 0.9198 | 0.9603 | 0.8996 | 0.9614 | 0.919 | 0.9676 | 0.9225 | 0.9662 |
| 1behA | 0.8967 | 0.9336 | 0.9026 | 0.9345 | 0.8911 | 0.9389 | 0.925 | 0.9524 | 0.9239 | 0.9518 | 0.9226 | 0.9527 | 0.9107 | 0.949 | 0.92 | 0.9522 | 0.9177 | 0.9552 |
| 1bkrA | 0.8926 | 0.9051 | 0.9023 | 0.8959 | 0.9279 | 0.9139 | 0.9191 | 0.9293 | 0.9482 | 0.9343 | 0.9469 | 0.9448 | 0.939 | 0.9408 | 0.9534 | 0.9421 | 0.9587 | 0.9513 |
| 1brfA | 0.7719 | 0.7853 | 0.8071 | 0.7505 | 0.7804 | 0.7778 | 0.8011 | 0.8316 | 0.8255 | 0.8275 | 0.8119 | 0.801 | 0.8104 | 0.8675 | 0.876 | 0.81 | 0.8697 | 0.8352 |
| 1bsgA | 0.9487 | 0.9481 | 0.9457 | 0.9581 | 0.9655 | 0.9615 | 0.9588 | 0.9639 | 0.9721 | 0.9712 | 0.9705 | 0.9725 | 0.9709 | 0.9699 | 0.9754 | 0.9684 | 0.9682 | 0.9684 |
| 1c44A | 0.821 | 0.8417 | 0.8287 | 0.8377 | 0.839 | 0.8259 | 0.88 | 0.8549 | 0.8843 | 0.8691 | 0.883 | 0.8684 | 0.8798 | 0.8671 | 0.9008 | 0.8422 | 0.8947 | 0.875 |
| 1c52A | 0.888 | 0.9165 | 0.8992 | 0.9101 | 0.9305 | 0.9235 | 0.9083 | 0.9352 | 0.919 | 0.9372 | 0.9361 | 0.9394 | 0.9174 | 0.9391 | 0.9425 | 0.9334 | 0.9438 | 0.9358 |
| 1c9oA | 0.7234 | 0.8217 | 0.7186 | 0.7863 | 0.7058 | 0.8184 | 0.7054 | 0.8005 | 0.7713 | 0.8785 | 0.7105 | 0.8435 | 0.7071 | 0.8308 | 0.7228 | 0.841 | 0.7223 | 0.8398 |
| 1cc8A | 0.7367 | 0.8167 | 0.7917 | 0.8441 | 0.8128 | 0.8527 | 0.8201 | 0.839 | 0.8515 | 0.8503 | 0.8125 | 0.8982 | 0.8288 | 0.8926 | 0.8839 | 0.9001 | 0.8533 | 0.9017 |
| 1chdA | 0.9461 | 0.9462 | 0.9398 | 0.952 | 0.9475 | 0.9597 | 0.9645 | 0.9639 | 0.9505 | 0.9595 | 0.9567 | 0.9628 | 0.9595 | 0.9653 | 0.9658 | 0.9594 | 0.9646 | 0.9572 |
| 1cjwA | 0.8221 | 0.9108 | 0.8295 | 0.9155 | 0.8213 | 0.9097 | 0.8559 | 0.9379 | 0.8576 | 0.9327 | 0.8622 | 0.942 | 0.8597 | 0.9393 | 0.8637 | 0.9526 | 0.8458 | 0.9398 |
| 1ckeA | 0.8681 | 0.8322 | 0.8648 | 0.8278 | 0.8981 | 0.8512 | 0.8848 | 0.8739 | 0.8921 | 0.8817 | 0.8949 | 0.8779 | 0.9012 | 0.8992 | 0.883 | 0.8976 | 0.9046 | 0.903 |
| 1ctfA | 0.821 | 0.753 | 0.8399 | 0.7952 | 0.8174 | 0.7875 | 0.7728 | 0.8248 | 0.8236 | 0.8542 | 0.8062 | 0.76 | 0.8233 | 0.7988 | 0.8224 | 0.834 | 0.8008 | 0.8028 |
| 1cxyA | 0.7626 | 0.7989 | 0.7933 | 0.835 | 0.784 | 0.8557 | 0.8008 | 0.881 | 0.8329 | 0.8483 | 0.8404 | 0.8558 | 0.8612 | 0.8566 | 0.8292 | 0.8669 | 0.8355 | 0.9048 |
| 1cznA | 0.9145 | 0.9266 | 0.935 | 0.9311 | 0.9377 | 0.9357 | 0.9454 | 0.9414 | 0.9572 | 0.946 | 0.9591 | 0.9421 | 0.9526 | 0.9512 | 0.961 | 0.9459 | 0.9591 | 0.9482 |
| 1d0qA | 0.8118 | 0.8294 | 0.8476 | 0.8428 | 0.8392 | 0.8304 | 0.8769 | 0.8316 | 0.912 | 0.8952 | 0.8866 | 0.9038 | 0.9352 | 0.906 | 0.8862 | 0.8881 | 0.8938 | 0.9055 |
| 1d1qA | 0.904 | 0.9298 | 0.9274 | 0.9291 | 0.925 | 0.9285 | 0.9315 | 0.9499 | 0.9299 | 0.9367 | 0.9283 | 0.9436 | 0.9412 | 0.9374 | 0.9419 | 0.9403 | 0.9395 | 0.9267 |
| 1d4oA | 0.9366 | 0.9331 | 0.9459 | 0.9304 | 0.9468 | 0.9454 | 0.9553 | 0.9434 | 0.9663 | 0.9551 | 0.9568 | 0.9622 | 0.9557 | 0.9576 | 0.9508 | 0.9495 | 0.9545 | 0.9544 |
| 1dbxA | 0.8338 | 0.9087 | 0.8283 | 0.917 | 0.841 | 0.914 | 0.8589 | 0.932 | 0.8633 | 0.9313 | 0.8848 | 0.942 | 0.8619 | 0.9298 | 0.8699 | 0.9242 | 0.8689 | 0.9357 |
| 1dixA | 0.8994 | 0.9115 | 0.9132 | 0.9166 | 0.8993 | 0.936 | 0.9073 | 0.9363 | 0.9134 | 0.9324 | 0.94 | 0.9396 | 0.9306 | 0.9505 | 0.9188 | 0.9397 | 0.9082 | 0.945 |
| 1dlwA | 0.8739 | 0.8927 | 0.9019 | 0.9036 | 0.9046 | 0.9025 | 0.8994 | 0.915 | 0.9433 | 0.9161 | 0.9214 | 0.9234 | 0.9575 | 0.9509 | 0.9663 | 0.9457 | 0.9388 | 0.9455 |
| 1dmgA | 0.8571 | 0.884 | 0.858 | 0.8787 | 0.8827 | 0.8854 | 0.865 | 0.8961 | 0.8954 | 0.9085 | 0.8687 | 0.8928 | 0.893 | 0.9097 | 0.8785 | 0.9091 | 0.862 | 0.9072 |
| 1dqgA | 0.8828 | 0.9097 | 0.883 | 0.9171 | 0.8839 | 0.9402 | 0.9158 | 0.9351 | 0.9251 | 0.9351 | 0.9136 | 0.9405 | 0.9206 | 0.9412 | 0.9239 | 0.9422 | 0.9063 | 0.9362 |
| 1dsxA | 0.8932 | 0.8774 | 0.8529 | 0.8425 | 0.8409 | 0.8648 | 0.8343 | 0.8573 | 0.8991 | 0.9119 | 0.9324 | 0.9169 | 0.9355 | 0.9151 | 0.9162 | 0.9206 | 0.9242 | 0.9219 |
| 1eazA | 0.8745 | 0.8819 | 0.8511 | 0.8883 | 0.884 | 0.8941 | 0.9036 | 0.9292 | 0.9305 | 0.9302 | 0.9158 | 0.9417 | 0.9091 | 0.9442 | 0.9102 | 0.9427 | 0.9362 | 0.9499 |
| 1ej0A | 0.9174 | 0.9103 | 0.9279 | 0.9305 | 0.9237 | 0.9411 | 0.9327 | 0.9397 | 0.9336 | 0.9422 | 0.9483 | 0.9558 | 0.9293 | 0.951 | 0.9448 | 0.9469 | 0.9303 | 0.9436 |
| 1ej8A | 0.8259 | 0.8427 | 0.808 | 0.865 | 0.87 | 0.9029 | 0.8684 | 0.9172 | 0.842 | 0.9332 | 0.8422 | 0.9283 | 0.8483 | 0.9348 | 0.8364 | 0.9362 | 0.8529 | 0.9354 |
| 1ek0A | 0.8958 | 0.902 | 0.899 | 0.8932 | 0.8765 | 0.9162 | 0.8919 | 0.9213 | 0.9193 | 0.9262 | 0.9265 | 0.9345 | 0.9189 | 0.941 | 0.9181 | 0.9466 | 0.9168 | 0.9439 |
| 1f6bA | 0.8258 | 0.8725 | 0.8773 | 0.8882 | 0.8563 | 0.8924 | 0.873 | 0.9004 | 0.8736 | 0.9029 | 0.895 | 0.9164 | 0.8968 | 0.9149 | 0.8779 | 0.9134 | 0.8822 | 0.9149 |
| 1fcyA | 0.9379 | 0.942 | 0.9534 | 0.9412 | 0.9577 | 0.9502 | 0.9543 | 0.9569 | 0.9674 | 0.964 | - | 0.9693 | 0.9665 | 0.9683 | 0.9607 | 0.9665 | 0.9677 | 0.9694 |
| 1fk5A | 0.8631 | 0.8569 | 0.8851 | 0.8506 | 0.8824 | 0.88 | 0.8802 | 0.8594 | 0.8763 | 0.8651 | 0.8689 | 0.8614 | 0.857 | 0.879 | 0.8431 | 0.8773 | 0.8873 | 0.8686 |
| 1fl0A | 0.9016 | 0.9142 | 0.8893 | 0.9314 | 0.9246 | 0.9282 | 0.916 | 0.9348 | 0.9156 | 0.9204 | 0.9216 | 0.9436 | 0.9261 | 0.951 | 0.9291 | 0.9561 | 0.9136 | 0.9517 |
| 1fnaA | 0.7947 | 0.806 | 0.8248 | 0.8519 | 0.7714 | 0.8626 | 0.8175 | 0.857 | 0.7885 | 0.8991 | 0.8831 | 0.8903 | 0.8889 | 0.8828 | 0.8857 | 0.8906 | 0.858 | 0.8982 |
| 1fqtA | 0.8724 | 0.8923 | 0.8589 | 0.879 | 0.8749 | 0.8967 | 0.856 | 0.9052 | 0.8823 | 0.9171 | 0.9018 | 0.9103 | 0.921 | 0.939 | 0.8939 | 0.921 | 0.9089 | 0.9308 |
| 1fvgA | 0.8783 | 0.9054 | 0.9096 | 0.95 | 0.9127 | 0.9386 | 0.9336 | 0.9542 | 0.929 | 0.9612 | 0.9339 | 0.9501 | 0.9403 | 0.9578 | 0.9493 | 0.953 | 0.9406 | 0.9392 |
| 1fvkA | 0.8981 | 0.8694 | 0.9067 | 0.8858 | 0.9284 | 0.903 | 0.9453 | 0.9299 | 0.9487 | 0.9468 | 0.9424 | 0.9329 | 0.9281 | 0.9413 | 0.9467 | 0.9443 | 0.9373 | 0.9382 |
| 1fx2A | 0.8005 | 0.7958 | 0.7094 | 0.8429 | 0.8299 | 0.8863 | 0.8237 | 0.8579 | 0.7672 | 0.906 | 0.6632 | 0.8694 | 0.7941 | 0.8339 | 0.8345 | 0.9071 | 0.8631 | 0.9022 |
| 1g2rA | 0.8276 | 0.8223 | 0.8314 | 0.8835 | 0.8475 | 0.8436 | 0.8581 | 0.8991 | 0.8917 | 0.9274 | 0.8935 | 0.9213 | 0.8782 | 0.927 | 0.9162 | 0.9047 | 0.8861 | 0.917 |
| 1g9oA | 0.7579 | 0.7917 | 0.8049 | 0.7975 | 0.8127 | 0.8194 | 0.7853 | 0.8202 | 0.7728 | 0.8738 | 0.8333 | 0.8859 | 0.8048 | 0.8794 | 0.831 | 0.8667 | 0.8233 | 0.8773 |
| 1gbsA | 0.8857 | 0.8953 | 0.9054 | 0.9152 | 0.8927 | 0.9268 | 0.9279 | 0.9161 | 0.9286 | 0.9227 | 0.9317 | 0.9299 | 0.9125 | 0.9266 | 0.9381 | 0.9245 | 0.9395 | 0.9258 |
| 1gmiA | 0.8046 | 0.8416 | 0.81 | 0.8592 | 0.805 | 0.8664 | 0.8273 | 0.8696 | 0.8412 | 0.8943 | 0.8378 | 0.8866 | 0.825 | 0.9058 | 0.88 | 0.9161 | 0.849 | 0.9201 |
| 1gmxA | 0.878 | 0.8715 | 0.883 | 0.8769 | 0.905 | 0.9018 | 0.9057 | 0.9176 | 0.9159 | 0.9167 | 0.9301 | 0.9296 | 0.9241 | 0.9297 | 0.9191 | 0.9228 | 0.9375 | 0.9197 |
| 1guuA | 0.858 | 0.7803 | 0.7863 | 0.7688 | 0.8773 | 0.8395 | 0.8602 | 0.8584 | 0.8326 | 0.8597 | 0.8734 | 0.8848 | 0.8782 | 0.8862 | 0.9018 | 0.9103 | 0.9025 | 0.8976 |
| 1gz2A | 0.8641 | 0.8885 | 0.8503 | 0.897 | 0.8689 | 0.8962 | 0.87 | 0.9124 | 0.8909 | 0.9229 | 0.8951 | 0.936 | 0.8927 | 0.9377 | 0.8908 | 0.9476 | 0.9129 | 0.9376 |
| 1gzcA | 0.9203 | 0.9452 | 0.934 | 0.9403 | 0.9359 | 0.9491 | 0.9381 | 0.9553 | 0.9581 | 0.9616 | 0.9514 | 0.966 | 0.9613 | 0.9734 | 0.9575 | 0.9684 | 0.9628 | 0.9725 |
| 1h0pA | 0.8648 | 0.9376 | 0.8959 | 0.9401 | 0.8998 | 0.9438 | 0.8937 | 0.9546 | 0.9185 | 0.9594 | 0.9167 | 0.9664 | 0.9327 | 0.9474 | 0.9091 | 0.9477 | 0.907 | 0.9506 |
| 1h2eA | 0.9222 | 0.9187 | 0.9252 | 0.942 | 0.9409 | 0.9566 | 0.9534 | 0.959 | 0.9511 | 0.9635 | 0.9538 | 0.9655 | 0.9598 | 0.9697 | 0.9569 | 0.9638 | 0.9414 | 0.9642 |
| 1h4xA | 0.7863 | 0.8921 | 0.7788 | 0.9111 | 0.8369 | 0.915 | 0.8464 | 0.9238 | 0.884 | 0.9373 | 0.8801 | 0.9336 | 0.8673 | 0.9329 | 0.873 | 0.9365 | 0.8815 | 0.9288 |
| 1h98A | 0.8642 | 0.8793 | 0.8288 | 0.8614 | 0.8479 | 0.8802 | 0.8973 | 0.8802 | 0.8585 | 0.9112 | 0.9139 | 0.9209 | 0.8776 | 0.9109 | 0.8983 | 0.9138 | 0.9237 | 0.891 |
| 1hdoA | 0.9428 | 0.9426 | 0.9481 | 0.9499 | 0.9561 | 0.9578 | 0.9557 | 0.9639 | 0.9625 | 0.963 | 0.9666 | 0.9589 | 0.9679 | 0.9607 | 0.9475 | 0.9584 | 0.971 | 0.961 |
| 1hfcA | 0.9003 | 0.9089 | 0.9246 | 0.9159 | 0.9266 | 0.9301 | 0.9357 | 0.9246 | 0.9546 | 0.9393 | 0.9408 | 0.9474 | 0.9329 | 0.952 | 0.9473 | 0.9488 | 0.9532 | 0.9369 |
| 1hh8A | 0.9068 | 0.8301 | 0.9359 | 0.8553 | 0.9371 | 0.8787 | 0.9527 | 0.9187 | 0.9683 | 0.9359 | 0.9613 | 0.9553 | 0.9613 | 0.9464 | 0.9586 | 0.9296 | 0.9699 | 0.9557 |
| 1htwA | 0.9296 | 0.9029 | 0.9365 | 0.9325 | 0.9336 | 0.9337 | 0.9514 | 0.9375 | 0.9637 | 0.9401 | 0.9558 | 0.9516 | 0.9626 | 0.9477 | 0.9658 | 0.9511 | 0.9656 | 0.9451 |
| 1hxnA | 0.9002 | 0.9283 | 0.9142 | 0.9307 | 0.9218 | 0.9393 | 0.9244 | 0.9459 | 0.9342 | 0.9469 | 0.9221 | 0.9458 | 0.9296 | 0.9572 | 0.9361 | 0.9559 | 0.9419 | 0.9582 |
| 1i1jA | 0.7618 | 0.6078 | 0.7795 | 0.6284 | 0.8183 | 0.8796 | 0.829 | 0.8841 | 0.8363 | 0.9031 | 0.8618 | 0.9031 | 0.8554 | 0.3215 | 0.8552 | 0.9089 | 0.8642 | 0.9043 |
| 1i1nA | 0.9289 | 0.9551 | 0.9346 | 0.95 | 0.942 | 0.9579 | 0.9466 | 0.9601 | 0.946 | 0.9623 | 0.9531 | 0.9603 | 0.946 | 0.955 | 0.9582 | 0.9576 | 0.9546 | 0.9634 |
| 1i4jA | 0.8113 | 0.8271 | 0.7931 | 0.8414 | 0.8082 | 0.8591 | 0.8247 | 0.8561 | 0.8257 | 0.865 | 0.8295 | 0.9015 | 0.8356 | 0.9204 | 0.8118 | 0.8946 | 0.7813 | 0.8995 |
| 1i58A | 0.8893 | 0.9029 | 0.9126 | 0.9163 | 0.9247 | 0.9255 | 0.9183 | 0.9316 | 0.9377 | 0.9325 | 0.935 | 0.9453 | 0.9432 | 0.9412 | 0.9454 | 0.9459 | 0.9391 | 0.9467 |
| 1i5gA | 0.8824 | 0.9136 | 0.8861 | 0.9235 | 0.9087 | 0.9324 | 0.923 | 0.9452 | 0.9161 | 0.9514 | 0.925 | 0.9471 | 0.9151 | 0.9485 | 0.9297 | 0.9527 | 0.9365 | 0.9519 |
| 1i71A | 0.7052 | 0.8159 | 0.7613 | 0.8382 | 0.8256 | 0.8667 | 0.7958 | 0.8603 | 0.8459 | 0.8433 | 0.8518 | 0.8628 | 0.8149 | 0.8702 | 0.7887 | 0.8902 | 0.8082 | 0.9065 |
| 1ihzA | 0.8645 | 0.8811 | 0.8795 | 0.9025 | 0.9085 | 0.9064 | 0.8925 | 0.9092 | 0.9186 | 0.9325 | 0.9219 | 0.9377 | 0.9248 | 0.9478 | 0.9078 | 0.9499 | 0.9186 | 0.9623 |
| 1iibA | 0.8821 | 0.8892 | 0.8823 | 0.8921 | 0.9132 | 0.8987 | 0.8959 | 0.9033 | 0.9398 | 0.8949 | 0.9282 | 0.9108 | 0.9347 | 0.9157 | 0.9241 | 0.9131 | 0.9251 | 0.8867 |
| 1im5A | 0.9095 | 0.9242 | 0.8968 | 0.9348 | 0.9078 | 0.9395 | 0.9342 | 0.9631 | 0.94 | 0.9603 | 0.9452 | 0.956 | 0.9257 | 0.9597 | 0.9537 | 0.9602 | 0.9424 | 0.9539 |
| 1iwdA | 0.9327 | 0.9387 | 0.9303 | 0.9492 | 0.95 | 0.9647 | 0.9336 | 0.9569 | 0.9394 | 0.953 | 0.9402 | 0.9559 | 0.9515 | 0.9556 | 0.9473 | 0.9526 | 0.9339 | 0.9553 |
| 1j3aA | 0.8616 | 0.9049 | 0.8843 | 0.9272 | 0.9023 | 0.9284 | 0.9192 | 0.9441 | 0.9051 | 0.9439 | 0.9158 | 0.9523 | 0.9376 | 0.9475 | 0.9221 | 0.9501 | 0.9457 | 0.9512 |
| 1jbeA | 0.8653 | 0.9042 | 0.9018 | 0.9114 | 0.9196 | 0.9197 | 0.9183 | 0.931 | 0.9065 | 0.9463 | 0.9308 | 0.9483 | 0.9369 | 0.9412 | 0.9162 | 0.9441 | 0.9294 | 0.9373 |
| 1jbkA | 0.9095 | 0.9285 | 0.9066 | 0.9231 | 0.9198 | 0.9288 | 0.9476 | 0.9407 | 0.9485 | 0.9533 | 0.9412 | 0.9508 | 0.9511 | 0.952 | 0.9542 | 0.9505 | 0.9516 | 0.9444 |
| 1jfuA | 0.887 | 0.9229 | 0.9172 | 0.9334 | 0.9286 | 0.9341 | 0.9315 | 0.9456 | 0.9416 | 0.9513 | 0.9349 | 0.9515 | 0.9293 | 0.9541 | 0.9305 | 0.9516 | 0.9271 | 0.9481 |
| 1jfxA | 0.9265 | 0.9413 | 0.9325 | 0.9366 | 0.9289 | 0.9286 | 0.943 | 0.9571 | 0.9434 | 0.9618 | 0.9493 | 0.9635 | 0.9374 | 0.9597 | 0.9578 | 0.9619 | 0.951 | 0.9638 |
| 1jkxA | 0.9085 | 0.9224 | 0.9108 | 0.9503 | 0.9103 | 0.9487 | 0.9444 | 0.9522 | 0.9329 | 0.9521 | 0.9385 | 0.9592 | 0.916 | 0.9573 | 0.9512 | 0.9685 | 0.9258 | 0.9681 |
| 1jl1A | 0.8901 | 0.9116 | 0.9088 | 0.9176 | 0.9317 | 0.9145 | 0.9313 | 0.9288 | 0.9118 | 0.9468 | 0.9232 | 0.9353 | 0.9292 | 0.95 | 0.9532 | 0.9453 | 0.9347 | 0.9401 |
| 1jo0A | 0.8378 | 0.8921 | 0.8666 | 0.9186 | 0.8508 | 0.9345 | 0.8434 | 0.9449 | 0.8189 | 0.9404 | 0.8497 | 0.9385 | 0.8143 | 0.9463 | 0.8648 | 0.95 | 0.8492 | 0.9535 |
| 1jo8A | 0.6665 | 0.7258 | 0.7371 | 0.8019 | 0.782 | 0.8531 | 0.7952 | 0.8891 | 0.7806 | 0.8348 | 0.8 | 0.8629 | 0.8131 | 0.8556 | 0.806 | 0.8879 | 0.7643 | 0.8833 |
| 1josA | 0.8274 | 0.8168 | 0.8084 | 0.8394 | 0.8236 | 0.8283 | 0.8605 | 0.8855 | 0.868 | 0.8808 | 0.8551 | 0.8874 | 0.8756 | 0.8956 | 0.8676 | 0.8992 | 0.8745 | 0.92 |
| 1jvwA | 0.8999 | 0.8376 | 0.8979 | 0.8733 | 0.8814 | 0.8973 | 0.8837 | 0.9137 | 0.894 | 0.9132 | 0.9041 | 0.9388 | 0.9029 | 0.935 | 0.8761 | 0.9228 | 0.8943 | 0.9134 |
| 1jwqA | 0.9282 | 0.94 | 0.9371 | 0.9476 | 0.9432 | 0.9542 | 0.9398 | 0.9555 | 0.9445 | 0.963 | 0.9539 | 0.9665 | 0.9564 | 0.9721 | 0.9493 | 0.9657 | 0.9542 | 0.97 |
| 1jyhA | 0.8136 | 0.7026 | 0.8059 | 0.7666 | 0.8742 | 0.9033 | 0.913 | 0.9401 | 0.9111 | 0.937 | 0.9247 | 0.9538 | 0.9282 | 0.9441 | 0.908 | 0.9393 | 0.9216 | 0.9425 |
| 1k6kA | 0.9361 | 0.8768 | 0.95 | 0.9235 | 0.9491 | 0.9295 | 0.9535 | 0.9418 | 0.9513 | 0.95 | 0.9342 | 0.9558 | 0.9368 | 0.9406 | 0.9588 | 0.9572 | 0.9335 | 0.9423 |
| 1k7cA | 0.941 | 0.9534 | 0.9413 | 0.9511 | 0.9635 | 0.9524 | 0.9694 | 0.9577 | 0.9674 | 0.9653 | 0.9723 | 0.9721 | 0.9687 | 0.9704 | 0.9706 | 0.9711 | 0.9732 | 0.9669 |
| 1k7jA | 0.914 | 0.9487 | 0.9189 | 0.9482 | 0.9248 | 0.9523 | 0.9469 | 0.97 | 0.9383 | 0.9708 | 0.9574 | 0.9679 | 0.9561 | 0.9655 | 0.9565 | 0.9687 | 0.9569 | 0.9727 |
| 1kidA | 0.8937 | 0.8983 | 0.9236 | 0.9063 | 0.921 | 0.9195 | 0.9202 | 0.9194 | 0.9407 | 0.9354 | 0.9221 | 0.9352 | 0.9456 | 0.9422 | 0.9294 | 0.9394 | 0.928 | 0.9449 |
| 1kq6A | 0.8205 | 0.862 | 0.8143 | 0.8725 | 0.8295 | 0.8705 | 0.8365 | 0.9179 | 0.8686 | 0.9159 | 0.8877 | 0.9269 | 0.8642 | 0.9013 | 0.8668 | 0.932 | 0.8676 | 0.9133 |
| 1kqrA | 0.8877 | 0.9116 | 0.8625 | 0.9102 | 0.8878 | 0.9243 | 0.9089 | 0.9263 | 0.8878 | 0.9387 | 0.8872 | 0.9332 | 0.8909 | 0.9376 | 0.8717 | 0.9458 | 0.8849 | 0.9458 |
| 1ktgA | 0.8604 | 0.9039 | 0.8438 | 0.9152 | 0.8429 | 0.9181 | 0.8579 | 0.9385 | 0.8537 | 0.9267 | 0.8643 | 0.9338 | 0.8965 | 0.9475 | 0.9139 | 0.9481 | 0.8505 | 0.9533 |
| 1ku3A | 0.7056 | 0.7961 | 0.8186 | 0.8303 | 0.8424 | 0.8411 | 0.8572 | 0.8616 | 0.8685 | 0.8686 | 0.891 | 0.8466 | 0.8846 | 0.9003 | 0.8687 | 0.8758 | 0.8717 | 0.8984 |
| 1kw4A | 0.8518 | 0.7973 | 0.8828 | 0.8508 | 0.8835 | 0.8773 | 0.8206 | 0.8674 | 0.9042 | 0.9014 | 0.9083 | 0.889 | 0.9033 | 0.888 | 0.8853 | 0.8951 | 0.8816 | 0.8811 |
| 1lm4A | 0.8461 | 0.8553 | 0.8579 | 0.881 | 0.8137 | 0.8748 | 0.8466 | 0.8944 | 0.8305 | 0.8862 | 0.8685 | 0.8932 | 0.8488 | 0.8884 | 0.8651 | 0.8905 | 0.8481 | 0.8972 |
| 1lo7A | 0.8526 | 0.8408 | 0.8605 | 0.8767 | 0.8499 | 0.8629 | 0.8192 | 0.8971 | 0.8781 | 0.9167 | 0.9013 | 0.9055 | 0.861 | 0.9008 | 0.8863 | 0.9217 | 0.882 | 0.9216 |
| 1lpyA | 0.85 | 0.821 | 0.8305 | 0.8635 | 0.8886 | 0.8833 | 0.8388 | 0.8931 | 0.872 | 0.903 | 0.8771 | 0.9065 | 0.9032 | 0.9238 | 0.8766 | 0.9317 | 0.8615 | 0.9402 |
| 1m4jA | 0.8383 | 0.8907 | 0.848 | 0.9261 | 0.8629 | 0.9315 | 0.903 | 0.9157 | 0.8826 | 0.9275 | 0.8886 | 0.9414 | 0.8804 | 0.9342 | 0.8463 | 0.9359 | 0.8652 | 0.9343 |
| 1m8aA | 0.6981 | 0.8043 | 0.7904 | 0.8222 | 0.7401 | 0.7879 | 0.7926 | 0.7973 | 0.7628 | 0.8395 | 0.7357 | 0.8851 | 0.8534 | 0.8651 | 0.7537 | 0.8694 | 0.7919 | 0.8902 |
| 1mk0A | 0.8502 | 0.9119 | 0.824 | 0.9153 | 0.8427 | 0.9079 | 0.8766 | 0.9301 | 0.9194 | 0.9451 | 0.9164 | 0.9524 | 0.8997 | 0.9556 | 0.8966 | 0.9334 | 0.8779 | 0.9304 |
| 1mugA | 0.8913 | 0.9252 | 0.9361 | 0.923 | 0.9346 | 0.9331 | 0.9395 | 0.941 | 0.9364 | 0.948 | 0.9416 | 0.9496 | 0.9341 | 0.9479 | 0.9415 | 0.9509 | 0.9409 | 0.9488 |
| 1nb9A | 0.8602 | 0.8605 | 0.8458 | 0.8784 | 0.8966 | 0.8994 | 0.8842 | 0.9257 | 0.8831 | 0.9336 | 0.8734 | 0.9343 | 0.8613 | 0.9439 | 0.9066 | 0.9391 | 0.8877 | 0.9361 |
| 1ne2A | 0.8725 | 0.8852 | 0.8468 | 0.8834 | 0.8729 | 0.899 | 0.8631 | 0.8918 | 0.8718 | 0.9019 | 0.8697 | 0.9117 | 0.9044 | 0.906 | 0.8867 | 0.9065 | 0.8858 | 0.9073 |
| 1npsA | 0.8641 | 0.8658 | 0.8692 | 0.8697 | 0.855 | 0.8892 | 0.8774 | 0.8938 | 0.8812 | 0.9214 | 0.8994 | 0.9211 | 0.8975 | 0.9306 | 0.9003 | 0.9108 | 0.9017 | 0.9122 |
| 1nrvA | 0.8643 | 0.8386 | 0.8872 | 0.8613 | 0.932 | 0.8966 | 0.9112 | 0.9149 | 0.9321 | 0.9103 | 0.9224 | 0.9361 | 0.9304 | 0.939 | 0.9279 | 0.9504 | 0.9113 | 0.9403 |
| 1ny1A | 0.9516 | 0.9553 | 0.9472 | 0.9539 | 0.959 | 0.9556 | 0.9575 | 0.9636 | 0.9601 | 0.9667 | 0.9682 | 0.9708 | 0.9613 | 0.9731 | 0.9723 | 0.977 | 0.9641 | 0.9733 |
| 1o1zA | 0.9089 | 0.9365 | 0.9337 | 0.9475 | 0.9433 | 0.9558 | 0.9465 | 0.9663 | 0.9576 | 0.9661 | 0.9549 | 0.9672 | 0.9602 | 0.9611 | 0.9399 | 0.9628 | 0.9591 | 0.9653 |
| 1p90A | 0.8641 | 0.8815 | 0.9023 | 0.8738 | 0.913 | 0.9023 | 0.9036 | 0.9151 | 0.9145 | 0.9318 | 0.9133 | 0.9393 | 0.9258 | 0.9367 | 0.926 | 0.939 | 0.9227 | 0.9394 |
| 1pchA | 0.9106 | 0.9277 | 0.9416 | 0.925 | 0.9392 | 0.9265 | 0.9421 | 0.9227 | 0.9496 | 0.933 | 0.9601 | 0.9354 | 0.9507 | 0.9319 | 0.9542 | 0.9409 | 0.9563 | 0.9303 |
| 1pkoA | 0.7952 | 0.8411 | 0.8163 | 0.8587 | 0.7558 | 0.8611 | 0.7215 | 0.8922 | 0.7696 | 0.8954 | 0.8136 | 0.8773 | 0.7972 | 0.903 | 0.7691 | 0.9045 | 0.8021 | 0.8923 |
| 1qf9A | 0.9305 | 0.9285 | 0.9197 | 0.947 | 0.9548 | 0.9521 | 0.9359 | 0.9554 | 0.9444 | 0.9557 | 0.9563 | 0.9565 | 0.9569 | 0.9657 | 0.9572 | 0.9647 | 0.9509 | 0.9644 |
| 1qjpA | 0.7107 | 0.791 | 0.7633 | 0.7719 | 0.7944 | 0.7642 | 0.7764 | 0.7937 | 0.8157 | 0.8203 | 0.8 | 0.836 | 0.8205 | 0.8707 | 0.8254 | 0.8905 | 0.8688 | 0.8941 |
| 1ql0A | 0.9324 | 0.9305 | 0.9334 | 0.9463 | 0.9343 | 0.9558 | 0.9446 | 0.966 | 0.9358 | 0.9686 | - | 0.9643 | 0.9579 | 0.9686 | 0.9619 | 0.969 | 0.9605 | 0.9688 |
| 1r26A | 0.882 | 0.885 | 0.8642 | 0.8994 | 0.8631 | 0.9247 | 0.8682 | 0.9281 | 0.8703 | 0.9486 | 0.8851 | 0.9405 | 0.8883 | 0.9463 | 0.8831 | 0.9481 | 0.8575 | 0.9553 |
| 1roaA | 0.7676 | 0.8515 | 0.8476 | 0.9003 | 0.8073 | 0.9224 | 0.8379 | 0.8857 | 0.8003 | 0.8798 | 0.8532 | 0.9073 | 0.8301 | 0.9036 | 0.8396 | 0.9032 | 0.8429 | 0.9196 |
| 1rw1A | 0.8708 | 0.9005 | 0.837 | 0.9033 | 0.9267 | 0.9176 | 0.9071 | 0.924 | 0.9323 | 0.9335 | 0.9506 | 0.9333 | 0.9252 | 0.9397 | 0.9372 | 0.9357 | 0.8994 | 0.9431 |
| 1rw7A | 0.9429 | 0.946 | 0.9541 | 0.9511 | 0.9519 | 0.9533 | 0.9555 | 0.9597 | 0.9441 | 0.9604 | 0.9608 | 0.95 | 0.958 | 0.9588 | 0.9697 | 0.9584 | 0.9676 | 0.9451 |
| 1rybA | 0.9284 | 0.9057 | 0.9148 | 0.9172 | 0.9308 | 0.9388 | 0.9441 | 0.9388 | 0.9365 | 0.937 | 0.9526 | 0.9384 | 0.947 | 0.939 | 0.9481 | 0.9372 | 0.9422 | 0.9418 |
| 1smxA | 0.6853 | 0.7306 | - | 0.7942 | 0.7947 | 0.8347 | 0.7874 | 0.8256 | 0.7615 | 0.8421 | 0.8063 | 0.8527 | 0.8387 | 0.8638 | 0.7732 | 0.8621 | 0.8076 | 0.8652 |
| 1svyA | 0.8698 | 0.8505 | 0.8698 | 0.894 | 0.9183 | 0.9225 | 0.9251 | 0.9093 | 0.9066 | 0.9237 | 0.9298 | 0.9439 | 0.9183 | 0.9295 | 0.9267 | 0.9367 | 0.8986 | 0.9196 |
| 1t8kA | 0.8409 | 0.9077 | 0.9372 | 0.9129 | 0.8889 | 0.9012 | 0.9029 | 0.8869 | 0.9107 | 0.9496 | 0.94 | 0.9455 | 0.9302 | 0.9447 | 0.9408 | 0.9514 | 0.9352 | 0.9395 |
| 1tifA | 0.7453 | 0.6819 | 0.7113 | 0.7081 | 0.6813 | 0.6963 | 0.8038 | 0.7596 | 0.8287 | 0.7474 | 0.8321 | 0.8248 | 0.869 | 0.857 | 0.8311 | 0.8125 | 0.8209 | 0.8213 |
| 1tqgA | 0.9352 | 0.9058 | 0.9287 | 0.9263 | 0.9518 | 0.9139 | 0.9427 | 0.946 | 0.9633 | 0.945 | 0.9574 | 0.959 | 0.9625 | 0.9462 | 0.9488 | 0.958 | 0.955 | 0.9495 |
| 1tqhA | 0.9226 | 0.9305 | 0.9473 | 0.9433 | 0.9636 | 0.9443 | 0.9557 | 0.967 | 0.9569 | 0.9604 | 0.9669 | 0.9716 | 0.9771 | 0.9738 | 0.963 | 0.9769 | 0.9707 | 0.9732 |
| 1tzvA | 0.9256 | 0.9244 | 0.9399 | 0.9456 | 0.9448 | 0.9486 | 0.955 | 0.9482 | 0.9427 | 0.9693 | 0.9418 | 0.9602 | 0.9285 | 0.9587 | 0.9652 | 0.9652 | 0.9628 | 0.9737 |
| 1vfyA | 0.5636 | 0.6441 | 0.6332 | 0.7346 | 0.6395 | 0.8188 | 0.6679 | 0.8192 | 0.6579 | 0.8343 | 0.6263 | 0.8166 | 0.6901 | 0.8679 | 0.6511 | 0.8833 | 0.6893 | 0.838 |
| 1vhuA | 0.9431 | 0.9534 | 0.9389 | 0.958 | 0.9452 | 0.954 | 0.9592 | 0.961 | 0.9621 | 0.9654 | 0.9611 | 0.9646 | 0.9571 | 0.9553 | 0.9615 | 0.9612 | 0.9517 | 0.9567 |
| 1vjkA | 0.8664 | 0.8599 | 0.8862 | 0.8658 | 0.9075 | 0.89 | 0.887 | 0.8976 | 0.9248 | 0.9071 | 0.9191 | 0.922 | 0.9252 | 0.9276 | 0.9298 | 0.9266 | 0.9116 | 0.938 |
| 1vmbA | 0.8019 | 0.8249 | 0.8065 | 0.8217 | 0.7964 | 0.8166 | 0.853 | 0.8316 | 0.8393 | 0.8831 | 0.8479 | 0.9141 | 0.8448 | 0.8988 | 0.8028 | 0.9061 | 0.852 | 0.9216 |
| 1vp6A | 0.8662 | 0.908 | 0.8691 | 0.9197 | 0.8667 | 0.9302 | 0.8885 | 0.9428 | 0.9086 | 0.9456 | 0.8981 | 0.9521 | 0.8864 | 0.9485 | 0.9149 | 0.9471 | 0.9206 | 0.9514 |
| 1w0hA | 0.9052 | 0.9353 | 0.8826 | 0.9441 | 0.9158 | 0.9559 | 0.9236 | 0.9506 | 0.9296 | 0.9595 | 0.9325 | 0.9666 | 0.9364 | 0.965 | 0.9318 | 0.968 | 0.9381 | 0.9648 |
| 1whiA | 0.8322 | 0.8763 | 0.827 | 0.8956 | 0.8409 | 0.8935 | 0.7931 | 0.9108 | 0.8124 | 0.9238 | 0.8189 | 0.9106 | 0.8487 | 0.9 | 0.8862 | 0.9187 | 0.8569 | 0.8801 |
| 1wjxA | 0.8162 | 0.8537 | 0.8589 | 0.8893 | 0.8364 | 0.8805 | 0.8718 | 0.9128 | 0.8383 | 0.9151 | 0.8748 | 0.9283 | 0.8751 | 0.9242 | 0.8662 | 0.9288 | 0.8645 | 0.9331 |
| 1wkcA | 0.9198 | 0.9089 | 0.9003 | 0.9258 | 0.9367 | 0.9235 | 0.9167 | 0.9442 | 0.9232 | 0.9414 | 0.9376 | 0.9469 | 0.94 | 0.9511 | 0.9468 | 0.9509 | 0.9392 | 0.9383 |
| 1xdzA | 0.9336 | 0.9433 | 0.9099 | 0.9446 | 0.9364 | 0.9514 | 0.9422 | 0.9541 | 0.9446 | 0.9623 | 0.9448 | 0.9627 | 0.9533 | 0.966 | 0.9529 | 0.9607 | 0.9493 | 0.9579 |
| 1xffA | 0.9449 | 0.9481 | 0.9542 | 0.9579 | 0.9578 | 0.9609 | 0.9613 | 0.9687 | 0.9693 | 0.9688 | 0.9763 | 0.9687 | 0.969 | 0.9674 | 0.973 | 0.9694 | 0.9712 | 0.9664 |
| 1xkrA | 0.9338 | 0.9195 | 0.9231 | 0.9353 | 0.9551 | 0.9534 | 0.9531 | 0.9504 | 0.958 | 0.9593 | 0.9613 | 0.9662 | 0.9715 | 0.9612 | 0.9662 | 0.9652 | 0.9665 | 0.9642 |
| 2arcA | 0.9092 | 0.9183 | 0.8805 | 0.9257 | 0.9139 | 0.9445 | 0.917 | 0.9539 | 0.9183 | 0.9568 | 0.9138 | 0.9553 | 0.9241 | 0.9603 | 0.9325 | 0.9576 | 0.9332 | 0.9563 |
| 2cuaA | 0.9265 | 0.8892 | 0.8836 | 0.9097 | 0.9153 | 0.925 | 0.888 | 0.932 | 0.9248 | 0.9312 | 0.8776 | 0.9368 | 0.9291 | 0.9369 | 0.9132 | 0.9323 | 0.9114 | 0.9224 |
| 2hs1A | 0.8225 | 0.8099 | 0.811 | 0.7852 | 0.7954 | 0.8634 | 0.8296 | 0.8891 | 0.8445 | 0.8993 | 0.8648 | 0.8665 | 0.8581 | 0.8659 | 0.8663 | 0.91 | 0.8798 | 0.9028 |
| 2mhrA | 0.919 | 0.9052 | 0.9442 | 0.9194 | 0.9419 | 0.9321 | 0.9418 | 0.9394 | 0.9598 | 0.9439 | 0.9477 | 0.9448 | 0.9661 | 0.9515 | 0.9278 | 0.9525 | 0.9548 | 0.965 |
| 2phyA | 0.877 | 0.9052 | 0.8883 | 0.9094 | 0.8753 | 0.9165 | 0.8964 | 0.9267 | 0.9123 | 0.9457 | 0.9107 | 0.9521 | 0.9544 | 0.9488 | 0.9194 | 0.9538 | 0.9244 | 0.9374 |
| 2tpsA | 0.9348 | 0.9421 | 0.9619 | 0.955 | 0.9698 | 0.9512 | 0.9648 | 0.9632 | 0.9697 | 0.9598 | 0.9707 | 0.9573 | 0.968 | 0.9634 | 0.9744 | 0.9652 | 0.9749 | 0.9625 |
| 2vxnA | 0.9416 | 0.954 | 0.9528 | 0.951 | 0.9183 | 0.9583 | 0.9641 | 0.9617 | 0.9709 | 0.9617 | 0.9573 | 0.9699 | 0.9789 | 0.9729 | 0.9698 | 0.9756 | 0.9667 | 0.9682 |
| 3borA | 0.9184 | 0.9339 | 0.9354 | 0.941 | 0.9415 | 0.9429 | 0.9475 | 0.9471 | 0.9373 | 0.9534 | 0.9421 | 0.9436 | 0.9516 | 0.9485 | 0.9483 | 0.9475 | 0.9549 | 0.9468 |
| 3dqgA | 0.7395 | 0.8137 | 0.7476 | 0.841 | 0.7516 | 0.8591 | 0.7986 | 0.8992 | 0.7481 | 0.9071 | 0.7996 | 0.8979 | 0.7807 | 0.8967 | 0.8526 | 0.9285 | 0.8603 | 0.9178 |
| 5ptpA | 0.9219 | 0.9448 | 0.9056 | 0.9517 | 0.9259 | 0.9564 | 0.9249 | 0.959 | 0.9325 | 0.9571 | 0.9193 | 0.9606 | 0.9259 | 0.9634 | 0.9336 | 0.9603 | 0.9352 | 0.9546 |
|  |  |  |  |  |  |  |  |  |  |  |  |  |  |  |  |  |  |  |
| Mean | 0.86 | 0.88 | 0.87 | 0.89 | 0.88 | 0.90 | 0.89 | 0.91 | 0.90 | 0.92 | 0.90 | 0.93 | 0.91 | 0.93 | 0.90 | 0.93 | 0.90 | 0.93 |
| Median | 0.87 | 0.90 | 0.88 | 0.91 | 0.90 | 0.92 | 0.90 | 0.93 | 0.92 | 0.93 | 0.92 | 0.94 | 0.92 | 0.94 | 0.92 | 0.94 | 0.92 | 0.94 |
