# Supplementary material for: Hybridized distance- and contact-based hierarchical structure modeling for folding soluble and membrane proteins
Source: PLoS Comput Biol. 2021 Feb 23;17(2):e1008753. doi: 10.1371/journal.pcbi.1008753 (PMC7935296; doi:10.1371/journal.pcbi.1008753)
Supplement: S3 Table — (DOCX) [file pcbi.1008753.s003.docx]

| **S3 Table.** Target-by-target reconstruction performance on 150 soluble proteins for true C_α_–C_α_ hybrid interaction maps at tri-level thresholding. | |
| --- | --- |
| 1a3aA | 0.9821 |
| 1a6mA | 0.98 |
| 1a70A | 0.9733 |
| 1aapA | 0.8596 |
| 1abaA | 0.9519 |
| 1ag6A | 0.9733 |
| 1aoeA | 0.9848 |
| 1atlA | 0.9882 |
| 1atzA | 0.934 |
| 1avsA | 0.9555 |
| 1bdoA | 0.9554 |
| 1bebA | 0.9849 |
| 1behA | 0.987 |
| 1bkrA | 0.9767 |
| 1brfA | 0.8998 |
| 1bsgA | 0.9916 |
| 1c44A | 0.932 |
| 1c52A | 0.9761 |
| 1c9oA | 0.9363 |
| 1cc8A | 0.9561 |
| 1chdA | 0.9858 |
| 1cjwA | 0.9655 |
| 1ckeA | 0.9241 |
| 1ctfA | 0.9374 |
| 1cxyA | 0.9528 |
| 1cznA | 0.9785 |
| 1d0qA | 0.9704 |
| 1d1qA | 0.977 |
| 1d4oA | 0.9815 |
| 1dbxA | 0.9682 |
| 1dixA | 0.9831 |
| 1dlwA | 0.9658 |
| 1dmgA | 0.9441 |
| 1dqgA | 0.9805 |
| 1dsxA | 0.9696 |
| 1eazA | 0.9699 |
| 1ej0A | 0.9829 |
| 1ej8A | 0.9762 |
| 1ek0A | 0.9755 |
| 1f6bA | 0.9569 |
| 1fcyA | 0.9879 |
| 1fk5A | 0.9528 |
| 1fl0A | 0.9823 |
| 1fnaA | 0.9624 |
| 1fqtA | 0.9744 |
| 1fvgA | 0.9818 |
| 1fvkA | 0.9803 |
| 1fx2A | 0.9411 |
| 1g2rA | 0.9546 |
| 1g9oA | 0.9185 |
| 1gbsA | 0.9793 |
| 1gmiA | 0.973 |
| 1gmxA | 0.9697 |
| 1guuA | 0.9274 |
| 1gz2A | 0.9631 |
| 1gzcA | 0.9884 |
| 1h0pA | 0.9875 |
| 1h2eA | 0.9882 |
| 1h4xA | 0.9795 |
| 1h98A | 0.9474 |
| 1hdoA | 0.9875 |
| 1hfcA | 0.9806 |
| 1hh8A | 0.9794 |
| 1htwA | 0.9808 |
| 1hxnA | 0.9844 |
| 1i1jA | 0.9522 |
| 1i1nA | 0.9883 |
| 1i4jA | 0.9251 |
| 1i58A | 0.9645 |
| 1i5gA | 0.9816 |
| 1i71A | 0.9528 |
| 1ihzA | 0.9747 |
| 1iibA | 0.9618 |
| 1im5A | 0.9853 |
| 1iwdA | 0.9873 |
| 1j3aA | 0.9708 |
| 1jbeA | 0.9747 |
| 1jbkA | 0.9754 |
| 1jfuA | 0.9812 |
| 1jfxA | 0.9916 |
| 1jkxA | 0.9845 |
| 1jl1A | 0.9742 |
| 1jo0A | 0.9767 |
| 1jo8A | 0.9451 |
| 1josA | 0.945 |
| 1jvwA | 0.9732 |
| 1jwqA | 0.9868 |
| 1jyhA | 0.9773 |
| 1k6kA | 0.9798 |
| 1k7cA | 0.9889 |
| 1k7jA | 0.9906 |
| 1kidA | 0.9763 |
| 1kq6A | 0.9496 |
| 1kqrA | 0.9775 |
| 1ktgA | 0.9648 |
| 1ku3A | 0.9281 |
| 1kw4A | 0.9486 |
| 1lm4A | 0.8353 |
| 1lo7A | 0.9721 |
| 1lpyA | 0.974 |
| 1m4jA | 0.9675 |
| 1m8aA | 0.9473 |
| 1mk0A | 0.9759 |
| 1mugA | 0.9816 |
| 1nb9A | 0.9681 |
| 1ne2A | 0.9416 |
| 1npsA | 0.9535 |
| 1nrvA | 0.9684 |
| 1ny1A | 0.9925 |
| 1o1zA | 0.9859 |
| 1p90A | 0.9783 |
| 1pchA | 0.9813 |
| 1pkoA | 0.9301 |
| 1qf9A | 0.9859 |
| 1qjpA | 0.915 |
| 1ql0A | 0.9891 |
| 1r26A | 0.9762 |
| 1roaA | 0.966 |
| 1rw1A | 0.9755 |
| 1rw7A | 0.988 |
| 1rybA | 0.9751 |
| 1smxA | 0.9336 |
| 1svyA | 0.967 |
| 1t8kA | 0.9648 |
| 1tifA | 0.8781 |
| 1tqgA | 0.9726 |
| 1tqhA | 0.989 |
| 1tzvA | 0.9745 |
| 1vfyA | 0.8971 |
| 1vhuA | 0.9847 |
| 1vjkA | 0.9689 |
| 1vmbA | 0.9424 |
| 1vp6A | 0.9779 |
| 1w0hA | 0.9897 |
| 1whiA | 0.9605 |
| 1wjxA | 0.9606 |
| 1wkcA | 0.9795 |
| 1xdzA | 0.9885 |
| 1xffA | 0.9915 |
| 1xkrA | 0.985 |
| 2arcA | 0.9811 |
| 2cuaA | 0.9771 |
| 2hs1A | 0.9623 |
| 2mhrA | 0.9808 |
| 2phyA | 0.9819 |
| 2tpsA | 0.992 |
| 2vxnA | 0.9874 |
| 3borA | 0.9683 |
| 3dqgA | 0.9337 |
| 5ptpA | 0.9857 |
|  |  |
| Mean | 0.966608667 |
| Median | 0.9749 |
