# Supplementary material for: Hybridized distance- and contact-based hierarchical structure modeling for folding soluble and membrane proteins
Source: PLoS Comput Biol. 2021 Feb 23;17(2):e1008753. doi: 10.1371/journal.pcbi.1008753 (PMC7935296; doi:10.1371/journal.pcbi.1008753)
Supplement: S4 Table — (DOCX) [file pcbi.1008753.s004.docx]

| **S4 Table.** Target-by-target reconstruction performance on 150 soluble proteins for true C_β_–C_β_ hybrid interaction maps at tri-level thresholding. | |
| --- | --- |
| 1a3aA | 0.9522 |
| 1a6mA | 0.9386 |
| 1a70A | 0.9318 |
| 1aapA | 0.7809 |
| 1abaA | 0.8931 |
| 1ag6A | 0.937 |
| 1aoeA | 0.9635 |
| 1atlA | 0.9674 |
| 1atzA | 0.9393 |
| 1avsA | 0.8859 |
| 1bdoA | 0.8996 |
| 1bebA | 0.9735 |
| 1behA | 0.9489 |
| 1bkrA | 0.9543 |
| 1brfA | 0.8717 |
| 1bsgA | 0.9617 |
| 1c44A | 0.8659 |
| 1c52A | 0.9312 |
| 1c9oA | 0.8678 |
| 1cc8A | 0.9017 |
| 1chdA | 0.9544 |
| 1cjwA | 0.9447 |
| 1ckeA | 0.8949 |
| 1ctfA | 0.8866 |
| 1cxyA | 0.9361 |
| 1cznA | 0.9458 |
| 1d0qA | 0.8763 |
| 1d1qA | 0.9499 |
| 1d4oA | 0.9531 |
| 1dbxA | 0.9264 |
| 1dixA | 0.9509 |
| 1dlwA | 0.9382 |
| 1dmgA | 0.9115 |
| 1dqgA | 0.9513 |
| 1dsxA | 0.9266 |
| 1eazA | 0.9541 |
| 1ej0A | 0.9494 |
| 1ej8A | 0.9397 |
| 1ek0A | 0.9483 |
| 1f6bA | 0.9183 |
| 1fcyA | 0.9687 |
| 1fk5A | 0.8909 |
| 1fl0A | 0.9531 |
| 1fnaA | 0.9138 |
| 1fqtA | 0.9295 |
| 1fvgA | 0.9499 |
| 1fvkA | 0.9345 |
| 1fx2A | 0.9194 |
| 1g2rA | 0.9194 |
| 1g9oA | 0.8824 |
| 1gbsA | 0.9442 |
| 1gmiA | 0.9504 |
| 1gmxA | 0.9266 |
| 1guuA | 0.9077 |
| 1gz2A | 0.9393 |
| 1gzcA | 0.9694 |
| 1h0pA | 0.9499 |
| 1h2eA | 0.9634 |
| 1h4xA | 0.9258 |
| 1h98A | 0.9271 |
| 1hdoA | 0.9598 |
| 1hfcA | 0.9477 |
| 1hh8A | 0.9635 |
| 1htwA | 0.9514 |
| 1hxnA | 0.9586 |
| 1i1jA | 0.331 |
| 1i1nA | 0.9551 |
| 1i4jA | 0.9102 |
| 1i58A | 0.937 |
| 1i5gA | 0.9596 |
| 1i71A | 0.8989 |
| 1ihzA | 0.9439 |
| 1iibA | 0.9056 |
| 1im5A | 0.9559 |
| 1iwdA | 0.9528 |
| 1j3aA | 0.9524 |
| 1jbeA | 0.9436 |
| 1jbkA | 0.9489 |
| 1jfuA | 0.9499 |
| 1jfxA | 0.9615 |
| 1jkxA | 0.9652 |
| 1jl1A | 0.9436 |
| 1jo0A | 0.952 |
| 1jo8A | 0.9096 |
| 1josA | 0.9302 |
| 1jvwA | 0.9146 |
| 1jwqA | 0.9694 |
| 1jyhA | 0.9404 |
| 1k6kA | 0.9531 |
| 1k7cA | 0.9701 |
| 1k7jA | 0.9668 |
| 1kidA | 0.9309 |
| 1kq6A | 0.9386 |
| 1kqrA | 0.9569 |
| 1ktgA | 0.9614 |
| 1ku3A | 0.9001 |
| 1kw4A | 0.9273 |
| 1lm4A | 0.8913 |
| 1lo7A | 0.9497 |
| 1lpyA | 0.9354 |
| 1m4jA | 0.9331 |
| 1m8aA | 0.9283 |
| 1mk0A | 0.9472 |
| 1mugA | 0.951 |
| 1nb9A | 0.9432 |
| 1ne2A | 0.8998 |
| 1npsA | 0.8947 |
| 1nrvA | 0.9555 |
| 1ny1A | 0.9709 |
| 1o1zA | 0.9632 |
| 1p90A | 0.9305 |
| 1pchA | 0.9476 |
| 1pkoA | 0.9038 |
| 1qf9A | 0.9585 |
| 1qjpA | 0.9104 |
| 1ql0A | 0.9676 |
| 1r26A | 0.9599 |
| 1roaA | 0.9401 |
| 1rw1A | 0.9462 |
| 1rw7A | 0.9524 |
| 1rybA | 0.9418 |
| 1smxA | 0.8576 |
| 1svyA | 0.9227 |
| 1t8kA | 0.947 |
| 1tifA | 0.8956 |
| 1tqgA | 0.9545 |
| 1tqhA | 0.9711 |
| 1tzvA | 0.9768 |
| 1vfyA | 0.8481 |
| 1vhuA | 0.9562 |
| 1vjkA | 0.9466 |
| 1vmbA | 0.9098 |
| 1vp6A | 0.943 |
| 1w0hA | 0.9678 |
| 1whiA | 0.8731 |
| 1wjxA | 0.9364 |
| 1wkcA | 0.9406 |
| 1xdzA | 0.9558 |
| 1xffA | 0.9604 |
| 1xkrA | 0.959 |
| 2arcA | 0.9489 |
| 2cuaA | 0.9326 |
| 2hs1A | 0.9147 |
| 2mhrA | 0.9612 |
| 2phyA | 0.9378 |
| 2tpsA | 0.9471 |
| 2vxnA | 0.9647 |
| 3borA | 0.9515 |
| 3dqgA | 0.907 |
| 5ptpA | 0.9498 |
|  |  |
| Mean | 0.931099333 |
| Median | 0.94375 |
