# Supplementary material for: Hybridized distance- and contact-based hierarchical structure modeling for folding soluble and membrane proteins
Source: PLoS Comput Biol. 2021 Feb 23;17(2):e1008753. doi: 10.1371/journal.pcbi.1008753 (PMC7935296; doi:10.1371/journal.pcbi.1008753)
Supplement: S5 Table — (DOCX) [file pcbi.1008753.s005.docx]

| **S5 Table.** Target-by-target *ab initio* folding performance on 40 CASP FM targets. | | | | | |  |
| --- | --- | --- | --- | --- | --- | --- |
| Targets | DConStruct | DMPfold | CONFOLD2 | ROSETTA | CGLFold | GDFuzz3D |
| T0859-D1 | 0.193 | 0.2446 | 0.1617 | 0.2232 | 0.19 | 0.1961 |
| T0862-D1 | 0.5056 | 0.2755 | 0.2599 | 0.3039 | 0.61 | 0.2081 |
| T0863-D1 | 0.503 | 0.2933 | 0.3374 | 0.2979 | 0.53 |  |
| T0863-D2 | 0.2296 | 0.1721 | 0.2728 | 0.2702 | 0.39 |  |
| T0864-D1 | 0.695 | 0.4792 | 0.5638 | 0.2935 | 0.28 | 0.2938 |
| T0866-D1 | 0.582 | 0.7369 | 0.6356 | 0.6355 | 0.55 | 0.7021 |
| T0869-D1 | 0.7448 | 0.7729 | 0.6522 | 0.7132 | 0.47 | 0.6056 |
| T0870-D1 | 0.6724 | 0.4912 | 0.5863 | 0.6267 | 0.56 | 0.467 |
| T0886-D1 | 0.3042 | 0.3214 | 0.3095 | 0.3277 | 0.29 | 0.3203 |
| T0886-D2 | 0.6944 | 0.6894 | 0.307 | 0.4633 | 0.5 | 0.5558 |
| T0892-D2 | 0.696 | 0.6411 | 0.5397 | 0.4258 | 0.35 | 0.556 |
| T0896-D3 | 0.1558 | 0.1627 | 0.1773 | 0.1566 | 0.22 |  |
| T0897-D1 | 0.2031 | 0.2187 | 0.2038 | 0.2262 | 0.2 | 0.2307 |
| T0897-D2 | 0.2122 | 0.2463 | 0.227 | 0.2518 | 0.29 | 0.2593 |
| T0898-D1 | 0.6463 | 0.3716 | 0.4882 | 0.3413 | 0.52 | 0.4488 |
| T0900-D1 | 0.6251 | 0.6207 | 0.2337 | 0.3583 | 0.45 | 0.2483 |
| T0904-D1 | 0.4335 | 0.4073 | 0.7023 | 0.6011 |  | 0.5478 |
| T0912-D3 | 0.5784 | 0.5599 | 0.3651 | 0.2007 | 0.26 |  |
| T0918-D1 | 0.5547 | 0.5858 | 0.2849 | 0.3806 | 0.43 |  |
| T0918-D2 | 0.3157 | 0.5632 | 0.41 | 0.3767 | 0.47 |  |
| T0918-D3 | 0.5148 | 0.4087 | 0.3084 | 0.4842 | 0.43 |  |
| T0941-D1 | 0.2739 | 0.288 | 0.1869 | 0.2293 | 0.26 |  |
| T0950-D1 | 0.5019 | 0.3062 | 0.3278 | 0.3339 | 0.26 | 0.2491 |
| T0953s1-D1 | 0.3997 | 0.3567 | 0.2486 | 0.2989 | 0.4 | 0.3172 |
| T0953s2-D2 | 0.6466 | 0.5258 | 0.464 | 0.4689 | 0.3 | 0.6744 |
| T0953s2-D3 | 0.5092 | 0.2404 | 0.2825 | 0.2266 |  | 0.4136 |
| T0957s1-D1 | 0.3496 | 0.214 | 0.3741 | 0.3645 |  | 0.3721 |
| T0957s2-D1 | 0.7022 | 0.5481 | 0.6063 | 0.5632 | 0.58 | 0.5198 |
| T0963-D2 | 0.2306 | 0.2244 | 0.2863 | 0.2732 | 0.45 | 0.2551 |
| T0968s1-D1 | 0.6823 | 0.6635 | 0.5202 | 0.5209 | 0.55 | 0.5901 |
| T0968s2-D1 | 0.7371 | 0.5256 | 0.6818 | 0.3609 | 0.54 | 0.5416 |
| T0960-D2 | 0.3601 | 0.25 | 0.2657 | 0.2546 | 0.41 | 0.2074 |
| T0969-D1 | 0.7688 | 0.5698 | 0.4974 | 0.4909 |  |  |
| T0980s1-D1 | 0.2905 | 0.2611 | 0.4544 | 0.4596 |  | 0.3195 |
| T0990-D1 | 0.3254 | 0.5156 | 0.2871 | 0.3605 |  |  |
| T0990-D2 | 0.256 | 0.3714 | 0.2563 | 0.3679 |  |  |
| T0990-D3 | 0.2479 | 0.2625 | 0.2414 | 0.2415 |  |  |
| T1021s3-D1 | 0.4904 | 0.3951 | 0.4469 | 0.4878 |  | 0.4588 |
| T1021s3-D2 | 0.3345 | 0.2975 | 0.2939 | 0.2854 |  | 0.2957 |
| T1022s1-D1 | 0.2682 | 0.5253 | 0.5316 | 0.4479 |  | 0.4955 |
|  |  |  |  |  |  |  |
| Mean | 0.46 | 0.42 | 0.38 | 0.37 | 0.40 | 0.41 |
| Median | 0.50 | 0.38 | 0.32 | 0.36 | 0.43 | 0.39 |
| Correct fold | 20 | 15 | 10 | 6 | 8 | 9 |
| P-value |  | 0.03329857 | 0.001616589 | 0.001311146 |  |  |
