# Supplementary material for: Hybridized distance- and contact-based hierarchical structure modeling for folding soluble and membrane proteins
Source: PLoS Comput Biol. 2021 Feb 23;17(2):e1008753. doi: 10.1371/journal.pcbi.1008753 (PMC7935296; doi:10.1371/journal.pcbi.1008753)
Supplement: S6 Table — (DOCX) [file pcbi.1008753.s006.docx]

**S6 Table.** Target-by-target *ab initio* folding performance on a subset of 29 CASP FM targets relevant to CGLFold.

| Targets | DConStruct | CGLFold |
| --- | --- | --- |
| T0859-D1 | 0.193 | 0.19 |
| T0862-D1 | 0.5056 | 0.61 |
| T0863-D1 | 0.503 | 0.53 |
| T0863-D2 | 0.2296 | 0.39 |
| T0864-D1 | 0.695 | 0.28 |
| T0866-D1 | 0.582 | 0.55 |
| T0869-D1 | 0.7448 | 0.47 |
| T0870-D1 | 0.6724 | 0.56 |
| T0886-D1 | 0.3042 | 0.29 |
| T0886-D2 | 0.6944 | 0.5 |
| T0892-D2 | 0.696 | 0.35 |
| T0896-D3 | 0.1558 | 0.22 |
| T0897-D1 | 0.2031 | 0.2 |
| T0897-D2 | 0.2122 | 0.29 |
| T0898-D1 | 0.6463 | 0.52 |
| T0900-D1 | 0.6251 | 0.45 |
| T0912-D3 | 0.5784 | 0.26 |
| T0918-D1 | 0.5547 | 0.43 |
| T0918-D2 | 0.3157 | 0.47 |
| T0918-D3 | 0.5148 | 0.43 |
| T0941-D1 | 0.2739 | 0.26 |
| T0950-D1 | 0.5019 | 0.26 |
| T0953s1-D1 | 0.3997 | 0.4 |
| T0953s2-D2 | 0.6466 | 0.3 |
| T0957s2-D1 | 0.7022 | 0.58 |
| T0963-D2 | 0.2306 | 0.45 |
| T0968s1-D1 | 0.6823 | 0.55 |
| T0968s2-D1 | 0.7371 | 0.54 |
| T0960-D2 | 0.3601 | 0.41 |
|  |  |  |
| Mean | **0.488293103** | 0.404828 |
| Median | **0.5148** | 0.43 |
| Correct Fold | **18** | 8 |
