# Supplementary material for: Hybridized distance- and contact-based hierarchical structure modeling for folding soluble and membrane proteins
Source: PLoS Comput Biol. 2021 Feb 23;17(2):e1008753. doi: 10.1371/journal.pcbi.1008753 (PMC7935296; doi:10.1371/journal.pcbi.1008753)
Supplement: S7 Table — (DOCX) [file pcbi.1008753.s007.docx]

**S7 Table.** Target-by-target *ab initio* folding performance on a subset of 28 CASP FM targets relevant to GDFuzz3D.

| Targets | DConStruct | GDFuzz3D |
| --- | --- | --- |
| T0859-D1 | 0.193 | 0.1961 |
| T0862-D1 | 0.5056 | 0.2081 |
| T0864-D1 | 0.695 | 0.2938 |
| T0866-D1 | 0.582 | 0.7021 |
| T0869-D1 | 0.7448 | 0.6056 |
| T0870-D1 | 0.6724 | 0.467 |
| T0886-D1 | 0.3042 | 0.3203 |
| T0886-D2 | 0.6944 | 0.5558 |
| T0892-D2 | 0.696 | 0.556 |
| T0897-D1 | 0.2031 | 0.2307 |
| T0897-D2 | 0.2122 | 0.2593 |
| T0898-D1 | 0.6463 | 0.4488 |
| T0900-D1 | 0.6251 | 0.2483 |
| T0904-D1 | 0.4335 | 0.5478 |
| T0950-D1 | 0.5019 | 0.2491 |
| T0953s1-D1 | 0.3997 | 0.3172 |
| T0953s2-D2 | 0.6466 | 0.6744 |
| T0953s2-D3 | 0.5092 | 0.4136 |
| T0957s1-D1 | 0.3496 | 0.3721 |
| T0957s2-D1 | 0.7022 | 0.5198 |
| T0963-D2 | 0.2306 | 0.2551 |
| T0968s1-D1 | 0.6823 | 0.5901 |
| T0968s2-D1 | 0.7371 | 0.5416 |
| T0960-D2 | 0.3601 | 0.2074 |
| T0980s1-D1 | 0.2905 | 0.3195 |
| T1021s3-D1 | 0.4904 | 0.4588 |
| T1021s3-D2 | 0.3345 | 0.2957 |
| T1022s1-D1 | 0.2682 | 0.4955 |
|  |  |  |
|  |  |  |
| Mean | **0.49** | 0.41 |
| Median | **0.50** | 0.39 |
| Correct fold | **15** | 9 |
