# Supplementary material for: Hybridized distance- and contact-based hierarchical structure modeling for folding soluble and membrane proteins
Source: PLoS Comput Biol. 2021 Feb 23;17(2):e1008753. doi: 10.1371/journal.pcbi.1008753 (PMC7935296; doi:10.1371/journal.pcbi.1008753)
Supplement: S8 Table — (DOCX) [file pcbi.1008753.s008.docx]

**S8 Table.** Target-by-target *ab initio* folding performance of top CASP human and server predictors, DConStruct with trRosetta distance maps, customized DMPfold, and trRosetta on 40 CASP FM targets.

| **Targets** | Length | RaptorX-Contact | A7D | BAKER-ROSETTASERVER | BAKER | Zhang-Server | Zhang | Jones-UCL | MULTICOM | DConStruct  (with trRosetta distance maps) | Customized DMPfold | trRosetta  (distance-only) |
| --- | --- | --- | --- | --- | --- | --- | --- | --- | --- | --- | --- | --- |
| T0859-D1 | 113 | 0.22 |  | 0.2 | 0.2 | 0.27 | 0.24 | 0.21 | 0.27 | 0.2056 | 0.2157 | 0.2099 |
| T0862-D1 | 93 | 0.27 |  | 0.47 | 0.48 | 0.46 | 0.39 | 0.5 | 0.34 | 0.5038 | 0.3449 | 0.2711 |
| T0863-D1 | 193 | 0.25 |  | 0.23 | 0.31 | 0.27 | 0.3 | 0.29 | 0.27 | 0.4263 | 0.4914 | 0.4425 |
| T0863-D2 | 356 | 0.15 |  | 0.25 | 0.25 | 0.16 | 0.18 | 0.2 | 0.19 | 0.2859 | 0.2774 | 0.3417 |
| T0864-D1 | 246 | 0.28 |  | 0.37 | 0.31 | 0.21 | 0.33 | 0.26 | 0.33 | 0.7345 | 0.2771 | 0.3093 |
| T0866-D1 | 104 | 0.45 |  | 0.79 | 0.79 | 0.51 | 0.49 | 0.72 | 0.49 | 0.7829 | 0.7094 | 0.8329 |
| T0869-D1 | 104 | 0.4 |  | 0.33 | 0.33 | 0.31 | 0.34 | - | 0.39 | 0.767 | 0.7686 | 0.4795 |
| T0870-D1 | 123 | 0.33 |  | 0.3 | 0.3 | 0.22 | 0.21 | 0.41 | 0.4 | 0.6888 | 0.678 | 0.2761 |
| T0886-D1 | 69 | 0.41 |  | 0.27 | 0.61 | 0.37 | 0.35 | 0.19 | 0.37 | 0.277 | 0.3155 | 0.3325 |
| T0886-D2 | 127 | 0.35 |  | 0.43 | 0.67 | 0.56 | 0.57 | 0.61 | 0.56 | 0.7532 | 0.6932 | 0.7739 |
| T0892-D2 | 110 | 0.34 |  | 0.34 | 0.46 | 0.51 | 0.49 | 0.4 | 0.41 | 0.6259 | 0.6664 | 0.6636 |
| T0896-D3 | 161 | 0.1 |  | 0.17 | 0.17 | 0.21 | 0.19 | 0.21 | 0.21 | 0.1825 | 0.2029 | 0.2184 |
| T0897-D1 | 138 | 0.16 |  | 0.2 | 0.19 | 0.26 | 0.24 | 0.18 | 0.21 | 0.2111 | 0.2578 | 0.2101 |
| T0897-D2 | 124 | 0.19 |  | 0.22 | 0.26 | 0.61 | 0.6 | 0.22 | 0.2 | 0.244 | 0.261 | 0.2553 |
| T0898-D1 | 106 | 0.29 |  | 0.33 | 0.33 | 0.38 | 0.36 | 0.31 | 0.35 | 0.7472 | 0.4447 | 0.7549 |
| T0900-D1 | 102 | 0.21 |  | 0.48 | 0.51 | 0.44 | 0.45 | 0.34 | 0.45 | 0.6832 | 0.6393 | 0.6837 |
| T0904-D1 | 251 | 0.28 |  | 0.48 | 0.47 | 0.46 | 0.46 | 0.46 | 0.46 | 0.7656 | 0.4301 | 0.5855 |
| T0912-D3 | 103 | - |  | 0.13 | 0.44 | 0.16 | 0.2 | 0.23 | 0.18 | 0.4944 | 0.6215 | 0.2769 |
| T0918-D1 | 108 | 0.31 |  | 0.17 | 0.49 | 0.49 | 0.49 | 0.52 | 0.48 | 0.5267 | 0.2637 | 0.5778 |
| T0918-D2 | 123 | 0.34 |  | 0.17 | 0.57 | 0.4 | 0.43 | 0.42 | 0.46 | 0.6422 | 0.317 | 0.3209 |
| T0918-D3 | 118 | 0.4 |  | 0.57 | 0.6 | 0.47 | 0.47 | 0.52 | 0.35 | 0.2568 | 0.5514 | 0.6726 |
| T0941-D1 | 341 | 0.19 |  | 0.19 | 0.19 | 0.23 | 0.22 | 0.16 | 0.19 | 0.2845 | 0.2693 | 0.2876 |
| T0950-D1 | 342 | 0.38 | - | 0.46 | - | 0.44 | - | - | - | 0.5072 | 0.5141 | 0.4675 |
| T0953s1-D1 | 67 | 0.23 | 0.49 | 0.19 | 0.38 | 0.4 | 0.36 | 0.34 | 0.4 | 0.394 | 0.3185 | 0.2548 |
| T0953s2-D2 | 111 | 0.73 | 0.61 | 0.47 | 0.62 | 0.59 | 0.72 | 0.61 | 0.58 | 0.6654 | 0.6721 | 0.3406 |
| T0953s2-D3 | 93 | 0.41 | 0.28 | 0.19 | 0.35 | 0.35 | 0.43 | 0.41 | 0.26 | 0.5214 | 0.5213 | 0.4468 |
| T0957s1-D1 | 108 | 0.33 | 0.57 | 0.42 | 0.49 | 0.38 | 0.44 | 0.42 | 0.4 | 0.4165 | 0.4238 | 0.2503 |
| T0957s2-D1 | 155 | 0.71 | 0.68 | 0.48 | 0.54 | 0.53 | 0.58 | 0.56 | 0.71 | 0.6769 | 0.6961 | 0.6992 |
| T0963-D2 | 82 | 0.51 | 0.39 | 0.36 | 0.36 | 0.48 | 0.58 | 0.23 | 0.49 | 0.3232 | 0.2272 | 0.3173 |
| T0968s1-D1 | 118 | 0.64 | 0.77 | 0.74 | 0.66 | 0.56 | 0.62 | 0.61 | 0.56 | 0.6922 | 0.7071 | 0.2833 |
| T0968s2-D1 | 115 | 0.69 | 0.83 | 0.66 | 0.77 | 0.62 | 0.7 | 0.5 | 0.77 | 0.716 | 0.7122 | 0.3296 |
| T0960-D2 | 84 | 0.41 | 0.39 | 0.28 | 0.28 | 0.39 | 0.55 | 0.3 | 0.38 | 0.2426 | 0.2462 | 0.2385 |
| T0969-D1 | 354 | 0.66 | 0.73 | 0.49 | 0.65 | 0.68 | 0.66 | 0.58 | 0.74 | 0.7936 | 0.4766 | 0.6726 |
| T0980s1-D1 | 104 | 0.43 | 0.43 | 0.41 | 0.41 | 0.29 | 0.44 | 0.33 | 0.54 | 0.4046 | 0.2803 | 0.3508 |
| T0990-D1 | 76 | 0.4 | 0.84 | 0.37 | 0.37 | 0.57 | 0.58 | 0.27 | 0.57 | 0.2919 | 0.2851 | 0.4413 |
| T0990-D2 | 231 | 0.35 | 0.59 | 0.26 | 0.26 | 0.38 | 0.44 | 0.27 | 0.38 | 0.3185 | 0.3672 | 0.25 |
| T0990-D3 | 213 | 0.22 | 0.64 | 0.23 | 0.23 | 0.21 | 0.24 | 0.24 | 0.21 | 0.2259 | 0.259 | 0.3056 |
| T1021s3-D1 | 166 | 0.63 | 0.73 | 0.5 | 0.51 | 0.63 | 0.62 | 0.68 | 0.63 | 0.5576 | 0.5746 | 0.6329 |
| T1021s3-D2 | 97 | 0.56 | 0.67 | 0.19 | 0.3 | 0.44 | 0.48 | 0.4 | 0.44 | 0.2326 | 0.2628 | 0.2666 |
| T1022s1-D1 | 156 | 0.57 | 0.59 | 0.4 | 0.6 | 0.69 | 0.62 | 0.6 | 0.69 | 0.5744 | 0.5127 | 0.5855 |
|  |  |  |  |  |  |  |  |  |  |  |  |  |
| Mean |  | 0.38 | 0.60 | 0.35 | 0.43 | 0.41 | 0.44 | 0.39 | 0.42 | 0.49 | 0.44 | 0.42 |
| Median |  | 0.35 | 0.61 | 0.34 | 0.41 | 0.42 | 0.44 | 0.37 | 0.4 | 0.51 | 0.43 | 0.34 |
| Correct Fold |  | 9 | 12 | 4 | 13 | 12 | 12 | 10 | 10 | 21 | 16 | 12 |
