# Supplementary material for: Hybridized distance- and contact-based hierarchical structure modeling for folding soluble and membrane proteins
Source: PLoS Comput Biol. 2021 Feb 23;17(2):e1008753. doi: 10.1371/journal.pcbi.1008753 (PMC7935296; doi:10.1371/journal.pcbi.1008753)
Supplement: S9 Table — (DOCX) [file pcbi.1008753.s009.docx]

| **S9 Table.** Target-by-target *ab initio* folding performance on 510 membrane proteins. | | |  |
| --- | --- | --- | --- |
| Target | DConStruct  (with DMPfold distance maps) | Xu's DTL with CNS | Dconstruct  (with trRosetta distance maps) |
| 1a0sP | 0.5269 | 0.2815 | 0.5523 |
| 1ar1B | 0.4934 | 0.651 | 0.6746 |
| 1bccE | 0.5235 | 0.2483 | 0.5557 |
| 1bctA | 0.4103 | 0.38 | 0.4218 |
| 1bhaA | 0.6311 | 0.6281 | 0.6435 |
| 1c17M | 0.4385 | 0.4613 | 0.4104 |
| 1e7pC | 0.6162 | 0.6473 | 0.6893 |
| 1ehkB | 0.2438 | 0.5301 | 0.2907 |
| 1fftB | 0.561 | 0.2299 | 0.6656 |
| 1fftC | 0.7429 | 0.5934 | 0.7513 |
| 1fw2A | 0.6645 | 0.2897 | 0.7766 |
| 1fx8A | 0.654 | 0.6239 | 0.7021 |
| 1gzmA | 0.4837 | 0.7332 | 0.5463 |
| 1h2sB | 0.5391 | 0.4751 | 0.6173 |
| 1h6s1 | 0.6223 | 0.6571 | 0.6416 |
| 1izlA | 0.3153 | 0.2777 | 0.2728 |
| 1izlC | 0.515 | 0.4025 | 0.3541 |
| 1jb0K | 0.4186 | 0.4397 | 0.4138 |
| 1k24A | 0.6996 | 0.5633 | 0.7453 |
| 1kf6C | 0.4795 | 0.4833 | 0.455 |
| 1kf6D | 0.556 | 0.4938 | 0.6042 |
| 1kqfB | 0.6082 | 0.2832 | 0.6136 |
| 1kqfC | 0.7434 | 0.6434 | 0.7069 |
| 1kzuA | 0.4865 | 0.4428 | 0.3815 |
| 1lghA | 0.5248 | 0.4573 | 0.4755 |
| 1m56B | 0.5316 | 0.399 | 0.7499 |
| 1m56D | 0.7187 | 0.6127 | 0.6781 |
| 1m57A | 0.8704 | 0.8187 | 0.8197 |
| 1mm4A | 0.2595 | 0.517 | 0.5536 |
| 1mprA | 0.2657 | 0.284 | 0.6611 |
| 1n7lA | 0.4774 | 0.5187 | 0.4871 |
| 1nekC | 0.6404 | 0.6401 | 0.6507 |
| 1nekD | 0.5867 | 0.706 | 0.6569 |
| 1o5wA | 0.7687 | 0.3439 | 0.7467 |
| 1occD | 0.3207 | 0.2525 | 0.3002 |
| 1oedC | 0.4874 | 0.3664 | 0.5037 |
| 1orsC | 0.5548 | 0.4434 | 0.4852 |
| 1p49A | 0.6714 | 0.573 | 0.6849 |
| 1p4tA | 0.7519 | 0.8164 | 0.7751 |
| 1p7bA | 0.3219 | 0.2976 | 0.3271 |
| 1pw4A | 0.7725 | 0.7793 | 0.7657 |
| 1q16C | 0.5805 | 0.6371 | 0.5622 |
| 1q90A | 0.3747 | 0.238 | 0.4484 |
| 1q90B | 0.6578 | 0.6961 | 0.7339 |
| 1qcrD | 0.3155 | 0.3692 | 0.3577 |
| 1qd6C | 0.69 | 0.6994 | 0.7821 |
| 1qleC | 0.7464 | 0.6321 | 0.7174 |
| 1rh5B | 0.3296 | 0.4668 | 0.5255 |
| 1rh5C | 0.5023 | 0.6246 | 0.4638 |
| 1rwtA | 0.4505 | 0.4808 | 0.4939 |
| 1s5lB | 0.2487 | 0.2838 | 0.29 |
| 1s5lE | 0.3049 | 0.2851 | 0.2866 |
| 1s5lX | 0.5962 | 0.668 | 0.6264 |
| 1sqqK | 0.4734 | 0.5394 | 0.4347 |
| 1t16A | 0.7173 | 0.6906 | 0.6538 |
| 1tlwA | 0.5881 | 0.5191 | 0.6368 |
| 1tqqA | 0.4957 | 0.492 | 0.587 |
| 1uunA | 0.6082 | 0.5962 | 0.5774 |
| 1uynX | 0.3806 | 0.7472 | 0.7632 |
| 1vclA | 0.2834 | 0.2259 | 0.2894 |
| 1vf5B | 0.3353 | 0.304 | 0.3777 |
| 1vf5D | 0.3171 | 0.2505 | 0.5192 |
| 1wrgA | 0.4174 | 0.3969 | 0.4404 |
| 1xioA | 0.8301 | 0.8187 | 0.8818 |
| 1xl4A | 0.4535 | 0.2865 | 0.395 |
| 1yc9A | 0.4663 | 0.3928 | 0.5116 |
| 1yewC | 0.5809 | 0.3008 | 0.5461 |
| 1yq3C | 0.5559 | 0.5304 | 0.5269 |
| 1yq3D | 0.6778 | 0.5006 | 0.7131 |
| 1zrtE | 0.2585 | 0.4679 | 0.2612 |
| 1zzaA | 0.2848 | 0.3265 | 0.283 |
| 2a0lA | 0.3574 | 0.3828 | 0.364 |
| 2a9hA | 0.6124 | 0.6239 | 0.6282 |
| 2akhA | 0.3107 | 0.3312 | 0.3312 |
| 2akhB | 0.5311 | 0.3755 | 0.6418 |
| 2bg9A | 0.3933 | 0.2753 | 0.5299 |
| 2bl2A | 0.8082 | 0.8331 | 0.7518 |
| 2cpbA | 0.2452 | 0.4598 | 0.4797 |
| 2d57A | 0.6245 | 0.715 | 0.8214 |
| 2ervA | 0.7336 | 0.6133 | 0.7323 |
| 2evuA | 0.6434 | 0.7779 | 0.7954 |
| 2f1cX | 0.6168 | 0.6544 | 0.652 |
| 2f93B | 0.4896 | 0.343 | 0.499 |
| 2f95B | 0.3856 | 0.3799 | 0.4697 |
| 2fynB | 0.5135 | 0.4227 | 0.6062 |
| 2ge4A | 0.562 | 0.6059 | 0.6046 |
| 2gfpA | 0.4583 | 0.4858 | 0.4488 |
| 2gr7A | 0.3878 | 0.2875 | 0.4588 |
| 2gr8A | 0.4775 | 0.4445 | 0.7368 |
| 2h8aA | 0.4811 | 0.4087 | 0.4098 |
| 2h8pC | 0.7068 | 0.568 | 0.6393 |
| 2hdfA | 0.7489 | 0.8037 | 0.7412 |
| 2ibzG | 0.2953 | 0.2729 | 0.3258 |
| 2ibzI | 0.4438 | 0.3764 | 0.4749 |
| 2iubA | 0.4246 | 0.6307 | 0.7318 |
| 2j58A | 0.2708 | 0.2477 | 0.333 |
| 2j7aC | 0.2457 | 0.3366 | 0.3108 |
| 2jafA | 0.8189 | 0.7918 | 0.8298 |
| 2jlnA | 0.6831 | 0.338 | 0.7657 |
| 2jo1A | 0.3378 | 0.3925 | 0.348 |
| 2jp3A | 0.3518 | 0.2967 | 0.3207 |
| 2k0lA | 0.4969 | 0.5511 | 0.5534 |
| 2k21A | 0.238 | 0.2082 | 0.2082 |
| 2k73A | 0.6629 | 0.6078 | 0.6849 |
| 2k9pA | 0.4038 | 0.3949 | 0.398 |
| 2kluA | 0.3387 | 0.3251 | 0.379 |
| 2kogA | 0.2466 | 0.2278 | 0.2733 |
| 2ks9A | 0.6674 | 0.7056 | 0.6856 |
| 2ksdA | 0.2958 | 0.3225 | 0.305 |
| 2kseA | 0.3718 | 0.3121 | 0.3816 |
| 2ksfA | 0.3109 | 0.3174 | 0.3172 |
| 2ksrA | 0.658 | 0.6323 | 0.6628 |
| 2kyhA | 0.5838 | 0.4183 | 0.4802 |
| 2l35A | 0.435 | 0.3813 | 0.3806 |
| 2l8sA | 0.4896 | 0.4847 | 0.4975 |
| 2lckA | 0.5268 | 0.5581 | 0.5728 |
| 2lhfA | 0.5887 | 0.6081 | 0.6357 |
| 2lkgA | 0.3927 | 0.3744 | 0.3824 |
| 2llyA | 0.5247 | 0.5304 | 0.5514 |
| 2lmeA | 0.3616 | 0.5203 | 0.3587 |
| 2lnlA | 0.4934 | 0.4946 | 0.4935 |
| 2lomA | 0.2924 | 0.2861 | 0.2604 |
| 2loqA | 0.2092 | 0.1977 | 0.2534 |
| 2lorA | 0.3229 | 0.3104 | 0.3448 |
| 2losA | 0.2545 | 0.2579 | 0.2565 |
| 2lotA | 0.3319 | 0.2867 | 0.3082 |
| 2lp1A | 0.3288 | 0.3181 | 0.3409 |
| 2m0qA | 0.2187 | 0.2923 | 0.273 |
| 2m20A | 0.3974 | 0.4096 | 0.3182 |
| 2m67A | 0.2536 | 0.2548 | 0.2929 |
| 2m6bA | 0.4999 | 0.4726 | 0.5128 |
| 2m7gA | 0.3132 | 0.3586 | 0.3032 |
| 2m8rA | 0.2885 | 0.2379 | 0.3328 |
| 2mafA | 0.4911 | 0.4976 | 0.4886 |
| 2mfrA | 0.3819 | 0.4094 | 0.4072 |
| 2mgyA | 0.5898 | 0.5636 | 0.5767 |
| 2mm8A | 0.1479 | 0.1544 | 0.1622 |
| 2mmuA | 0.4418 | 0.4572 | 0.4742 |
| 2mn6A | 0.4084 | 0.4014 | 0.4223 |
| 2mpnA | 0.3132 | 0.3182 | 0.3178 |
| 2mxbA | 0.4022 | 0.6764 | 0.439 |
| 2n4xA | 0.199 | 0.2566 | 0.2361 |
| 2n6lA | 0.4541 | 0.4381 | 0.4838 |
| 2n7qA | 0.5217 | 0.5783 | 0.5949 |
| 2nmrA | 0.6475 | 0.7519 | 0.6364 |
| 2nq2A | 0.8088 | 0.8165 | 0.7365 |
| 2nr9A | 0.7935 | 0.7991 | 0.7772 |
| 2nrgA | 0.289 | 0.3023 | 0.2753 |
| 2o01F | 0.2991 | 0.3315 | 0.3693 |
| 2oarA | 0.2649 | 0.2944 | 0.3107 |
| 2pnoA | 0.5729 | 0.7261 | 0.7294 |
| 2q67A | 0.6807 | 0.6934 | 0.5886 |
| 2q7mA | 0.6188 | 0.747 | 0.7451 |
| 2qomA | 0.7164 | 0.7422 | 0.6901 |
| 2r6gF | 0.3302 | 0.2109 | 0.4126 |
| 2r6gG | 0.4308 | 0.7224 | 0.4814 |
| 2vpwC | 0.6364 | 0.7857 | 0.6631 |
| 2w1pA | 0.6052 | 0.4157 | 0.7338 |
| 2wjqA | 0.6449 | 0.3183 | 0.6685 |
| 2wpdJ | 0.6637 | 0.6021 | 0.6364 |
| 2wpvB | 0.3113 | 0.3071 | 0.3222 |
| 2wsc1 | 0.2946 | 0.2357 | 0.3148 |
| 2wsc3 | 0.2647 | 0.2373 | 0.292 |
| 2wscF | 0.3147 | 0.324 | 0.3621 |
| 2wscG | 0.2313 | 0.193 | 0.2708 |
| 2wscH | 0.2638 | 0.1855 | 0.2609 |
| 2wscK | 0.266 | 0.2404 | 0.2922 |
| 2wscL | 0.385 | 0.4405 | 0.4427 |
| 2wswA | 0.7736 | 0.7599 | 0.827 |
| 2wwbB | 0.2532 | 0.3536 | 0.3499 |
| 2wwbC | 0.4406 | 0.4106 | 0.4607 |
| 2x4mA | 0.7129 | 0.335 | 0.7124 |
| 2xq2A | 0.5796 | 0.3434 | 0.6372 |
| 2xutA | 0.7523 | 0.7454 | 0.7382 |
| 2y5yA | 0.7956 | 0.7817 | 0.8002 |
| 2y69D | 0.2917 | 0.2641 | 0.2721 |
| 2y69G | 0.2654 | 0.2445 | 0.3428 |
| 2y69I | 0.3457 | 0.4361 | 0.3694 |
| 2y69J | 0.407 | 0.4419 | 0.446 |
| 2y69K | 0.3978 | 0.3867 | 0.4271 |
| 2y69L | 0.4056 | 0.4621 | 0.4371 |
| 2y69M | 0.426 | 0.4614 | 0.4336 |
| 2yevB | 0.5969 | 0.4294 | 0.6954 |
| 2yevC | 0.7111 | 0.6005 | 0.6037 |
| 2yiuA | 0.7255 | 0.5086 | 0.739 |
| 2ynkA | 0.6681 | 0.2935 | 0.6694 |
| 2z73A | 0.5899 | 0.2948 | 0.5931 |
| 2ziyA | 0.3481 | 0.7057 | 0.5978 |
| 2zjsE | 0.6019 | 0.4842 | 0.59 |
| 2zxeB | 0.5177 | 0.2496 | 0.628 |
| 2zxeG | 0.4656 | 0.5955 | 0.5375 |
| 3a2sX | 0.5921 | 0.6867 | 0.642 |
| 3a7kA | 0.8192 | 0.7488 | 0.7898 |
| 3anzA | 0.3625 | 0.4512 | 0.4777 |
| 3b4rA | 0.6689 | 0.569 | 0.6855 |
| 3b5dA | 0.625 | 0.4111 | 0.5744 |
| 3b9wA | 0.5918 | 0.4255 | 0.5171 |
| 3bryA | 0.7026 | 0.3173 | 0.6566 |
| 3chxB | 0.4165 | 0.2232 | 0.4289 |
| 3chxC | 0.7087 | 0.4695 | 0.62 |
| 3cn5A | 0.6137 | 0.5536 | 0.813 |
| 3cx5C | 0.7911 | 0.7107 | 0.7874 |
| 3d31C | 0.5306 | 0.6618 | 0.5397 |
| 3ddlA | 0.7723 | 0.7807 | 0.8137 |
| 3dhwA | 0.6188 | 0.782 | 0.6455 |
| 3dinE | 0.3319 | 0.3446 | 0.3558 |
| 3dl8C | 0.573 | 0.77 | 0.6995 |
| 3dl8E | 0.3563 | 0.3891 | 0.3455 |
| 3dwoX | 0.7049 | 0.645 | 0.6842 |
| 3dwwA | 0.5136 | 0.4331 | 0.4933 |
| 3dzmA | 0.3355 | 0.2757 | 0.4908 |
| 3effK | 0.4821 | 0.5159 | 0.5265 |
| 3eh3A | 0.7495 | 0.6252 | 0.7617 |
| 3ejzA | 0.8617 | 0.7434 | 0.8345 |
| 3emnX | 0.6038 | 0.5523 | 0.8134 |
| 3emoA | 0.2366 | 0.2862 | 0.4753 |
| 3fhhA | 0.7555 | 0.7939 | 0.7398 |
| 3fidA | 0.7199 | 0.2853 | 0.7153 |
| 3g67A | 0.2347 | 0.4816 | 0.2872 |
| 3gi8C | 0.7914 | 0.7468 | 0.7815 |
| 3hd6A | 0.5817 | 0.4131 | 0.623 |
| 3hw9A | 0.532 | 0.291 | 0.6677 |
| 3iyzA | 0.6 | 0.7739 | 0.7469 |
| 3iz1A | 0.5221 | 0.6045 | 0.6002 |
| 3j08A | 0.2722 | 0.3292 | 0.549 |
| 3j1zP | 0.5173 | 0.5538 | 0.5408 |
| 3j9tR | 0.7322 | 0.7646 | 0.7629 |
| 3jbrE | 0.5363 | 0.5389 | 0.5236 |
| 3jcuD | 0.2071 | 0.2185 | 0.278 |
| 3jcuH | 0.4147 | 0.434 | 0.4228 |
| 3jcuK | 0.3805 | 0.3667 | 0.4084 |
| 3jcuR | 0.5059 | 0.4017 | 0.4899 |
| 3jcuS | 0.4908 | 0.3986 | 0.5031 |
| 3jcuW | 0.4565 | 0.4223 | 0.449 |
| 3jcuX | 0.415 | 0.6773 | 0.6501 |
| 3jcuZ | 0.676 | 0.6279 | 0.6394 |
| 3jycA | 0.3133 | 0.294 | 0.329 |
| 3k3fA | 0.809 | 0.7263 | 0.7721 |
| 3kj6A | 0.7528 | 0.8199 | 0.8152 |
| 3kp9A | 0.425 | 0.4483 | 0.4318 |
| 3kvnA | 0.4614 | 0.3454 | 0.4786 |
| 3l1lA | 0.7796 | 0.8534 | 0.758 |
| 3lnmB | 0.306 | 0.3101 | 0.3492 |
| 3lw54 | 0.3932 | 0.38 | 0.4242 |
| 3lw5H | 0.2353 | 0.2484 | 0.2858 |
| 3m71A | 0.8324 | 0.8828 | 0.8544 |
| 3mk7A | 0.7962 | 0.8205 | 0.8041 |
| 3mk7B | 0.5235 | 0.2603 | 0.5028 |
| 3mk7C | 0.2503 | 0.2057 | 0.2974 |
| 3mktA | 0.7518 | 0.7786 | 0.738 |
| 3mp7A | 0.4221 | 0.4096 | 0.668 |
| 3mp7B | 0.3917 | 0.4711 | 0.4435 |
| 3njtA | 0.438 | 0.2536 | 0.496 |
| 3nymA | 0.356 | 0.4003 | 0.4409 |
| 3o0rB | 0.8283 | 0.8475 | 0.8078 |
| 3o7pA | 0.7098 | 0.7546 | 0.7443 |
| 3ohnA | 0.4836 | 0.2714 | 0.6254 |
| 3orgA | 0.5042 | 0.4766 | 0.7013 |
| 3oufA | 0.7368 | 0.5915 | 0.6867 |
| 3p5nA | 0.7939 | 0.8294 | 0.7591 |
| 3pjsK | 0.5094 | 0.5404 | 0.5417 |
| 3pjzA | 0.7995 | 0.7571 | 0.7798 |
| 3pwhA | 0.7371 | 0.8031 | 0.7893 |
| 3q7kA | 0.6235 | 0.7033 | 0.74 |
| 3qe7A | 0.7341 | 0.5936 | 0.712 |
| 3qnqA | 0.4609 | 0.4555 | 0.4518 |
| 3qraA | 0.727 | 0.7347 | 0.7873 |
| 3rbzA | 0.4813 | 0.4529 | 0.4104 |
| 3rgwS | 0.7305 | 0.3446 | 0.7936 |
| 3rkoA | 0.4705 | 0.4831 | 0.4316 |
| 3rkoB | 0.7773 | 0.8133 | 0.7572 |
| 3rkoC | 0.8831 | 0.8926 | 0.8696 |
| 3rkoD | 0.7656 | 0.8324 | 0.8442 |
| 3rkoF | 0.3956 | 0.4901 | 0.4534 |
| 3rkoG | 0.628 | 0.7313 | 0.6908 |
| 3s0xA | 0.502 | 0.4617 | 0.5652 |
| 3sljA | 0.7694 | 0.5647 | 0.7533 |
| 3sybA | 0.6238 | 0.2713 | 0.6763 |
| 3tijA | 0.6332 | 0.5008 | 0.6487 |
| 3tx3A | 0.6225 | 0.7126 | 0.7118 |
| 3udcA | 0.3595 | 0.3383 | 0.3966 |
| 3ug9A | 0.5215 | 0.5921 | 0.6132 |
| 3ukmA | 0.447 | 0.3512 | 0.643 |
| 3um7A | 0.3764 | 0.4527 | 0.5569 |
| 3uq7A | 0.4548 | 0.3376 | 0.3701 |
| 3ux4A | 0.658 | 0.6959 | 0.7873 |
| 3v2wA | 0.4069 | 0.5358 | 0.5717 |
| 3v5sA | 0.7771 | 0.714 | 0.7734 |
| 3vmqA | 0.7461 | 0.5257 | 0.6977 |
| 3vouA | 0.3278 | 0.5042 | 0.415 |
| 3vr8C | 0.3862 | 0.5007 | 0.4799 |
| 3vr8D | 0.5395 | 0.4983 | 0.5354 |
| 3vwiA | 0.7459 | 0.6482 | 0.7641 |
| 3wdoA | 0.6548 | 0.4826 | 0.6482 |
| 3wmfA | 0.3968 | 0.3838 | 0.4282 |
| 3wmm1 | 0.4146 | 0.522 | 0.3994 |
| 3wmmM | 0.4311 | 0.476 | 0.5795 |
| 3wo7A | 0.6794 | 0.5283 | 0.6233 |
| 3wvfA | 0.4714 | 0.2973 | 0.6562 |
| 3wxvA | 0.7971 | 0.7177 | 0.7838 |
| 3x29A | 0.7871 | 0.8011 | 0.7968 |
| 3x2rA | 0.4121 | 0.411 | 0.591 |
| 3x3bA | 0.7106 | 0.5401 | 0.7797 |
| 3ze3A | 0.6792 | 0.539 | 0.6705 |
| 3zevA | 0.7579 | 0.8241 | 0.8115 |
| 3zjzA | 0.644 | 0.7247 | 0.6264 |
| 3zk1A | 0.533 | 0.5287 | 0.5241 |
| 3zuxA | 0.7555 | 0.6698 | 0.8018 |
| 4a2nB | 0.4887 | 0.5256 | 0.5454 |
| 4atvA | 0.788 | 0.7374 | 0.7635 |
| 4aw6A | 0.8146 | 0.5563 | 0.8729 |
| 4b4aA | 0.5988 | 0.6279 | 0.6388 |
| 4bemJ | 0.6619 | 0.7172 | 0.6391 |
| 4bgnA | 0.435 | 0.457 | 0.3866 |
| 4bog3 | 0.3463 | 0.2481 | 0.4833 |
| 4bpmA | 0.5268 | 0.6695 | 0.4769 |
| 4bwzA | 0.6707 | 0.7494 | 0.6809 |
| 4c9jA | 0.6974 | 0.7378 | 0.7102 |
| 4cadC | 0.6688 | 0.6779 | 0.6978 |
| 4cfgA | 0.1658 | 0.2822 | 0.2175 |
| 4chvA | 0.3095 | 0.3621 | 0.3897 |
| 4cskA | 0.6 | 0.6345 | 0.8001 |
| 4czbA | 0.7274 | 0.8492 | 0.6993 |
| 4d5bA | 0.4624 | 0.236 | 0.5663 |
| 4d6tD | 0.6198 | 0.4858 | 0.7124 |
| 4d6tG | 0.3183 | 0.3553 | 0.3158 |
| 4d6tJ | 0.4831 | 0.3882 | 0.3712 |
| 4d6uD | 0.5639 | 0.3104 | 0.6505 |
| 4djiA | 0.722 | 0.4747 | 0.6993 |
| 4dojA | 0.7083 | 0.648 | 0.8073 |
| 4dveA | 0.7842 | 0.736 | 0.7675 |
| 4dxwA | 0.4014 | 0.4211 | 0.3224 |
| 4e1tA | 0.7204 | 0.7013 | 0.7611 |
| 4ea3A | 0.8219 | 0.668 | 0.8477 |
| 4ezcA | 0.6454 | 0.6065 | 0.7694 |
| 4f35A | 0.6594 | 0.6328 | 0.642 |
| 4f4lA | 0.6626 | 0.7303 | 0.7043 |
| 4fqeA | 0.6701 | 0.7767 | 0.727 |
| 4fuvA | 0.5398 | 0.3823 | 0.5568 |
| 4g1uA | 0.8449 | 0.3981 | 0.795 |
| 4g7vS | 0.6707 | 0.7479 | 0.6811 |
| 4g80I | 0.6328 | 0.669 | 0.6041 |
| 4gbyA | 0.87 | 0.8771 | 0.8524 |
| 4gd3A | 0.6493 | 0.6337 | 0.6919 |
| 4gx5A | 0.379 | 0.2974 | 0.4129 |
| 4gycB | 0.4636 | 0.3227 | 0.4522 |
| 4h33A | 0.5708 | 0.6844 | 0.6251 |
| 4he8A | 0.5192 | 0.4227 | 0.4389 |
| 4he8C | 0.7757 | 0.6369 | 0.751 |
| 4he8D | 0.4096 | 0.4894 | 0.4707 |
| 4hkrA | 0.4304 | 0.5478 | 0.568 |
| 4hqjE | 0.6997 | 0.6634 | 0.564 |
| 4httA | 0.3703 | 0.4451 | 0.6321 |
| 4huqS | 0.7089 | 0.7746 | 0.7234 |
| 4huqT | 0.4824 | 0.4769 | 0.5739 |
| 4hw9A | 0.2708 | 0.3283 | 0.3863 |
| 4hycA | 0.709 | 0.4693 | 0.7531 |
| 4hyoA | 0.7659 | 0.6789 | 0.7498 |
| 4hzuS | 0.7682 | 0.8406 | 0.7996 |
| 4iffA | 0.3658 | 0.4807 | 0.4003 |
| 4il3A | 0.8274 | 0.5235 | 0.8418 |
| 4in5H | 0.3293 | 0.4018 | 0.6392 |
| 4in5L | 0.4116 | 0.5423 | 0.5962 |
| 4j05A | 0.8409 | 0.8879 | 0.8568 |
| 4j72A | 0.7775 | 0.7985 | 0.778 |
| 4j7cI | 0.7474 | 0.6315 | 0.7081 |
| 4jkvA | 0.4171 | 0.5273 | 0.5972 |
| 4k1cA | 0.6228 | 0.6158 | 0.7059 |
| 4kjrA | 0.8065 | 0.7262 | 0.7768 |
| 4knfA | 0.8399 | 0.7806 | 0.8541 |
| 4kppA | 0.6619 | 0.5542 | 0.6787 |
| 4kt0F | 0.261 | 0.3246 | 0.3927 |
| 4kt0K | 0.5048 | 0.4736 | 0.376 |
| 4ky0A | 0.635 | 0.4199 | 0.5882 |
| 4l6rA | 0.5337 | 0.5422 | 0.6249 |
| 4l6v6 | 0.3761 | 0.3959 | 0.4336 |
| 4l6v8 | 0.5395 | 0.5715 | 0.5459 |
| 4ltoA | 0.5488 | 0.5733 | 0.6245 |
| 4m58A | 0.7495 | 0.7513 | 0.7615 |
| 4m64A | 0.7119 | 0.473 | 0.7421 |
| 4mbsA | 0.6698 | 0.6489 | 0.7293 |
| 4meeA | 0.6672 | 0.4395 | 0.7659 |
| 4mndA | 0.4946 | 0.318 | 0.5377 |
| 4mqsA | 0.7275 | 0.8229 | 0.8118 |
| 4mt4A | 0.4275 | 0.4304 | 0.5052 |
| 4n74A | 0.7335 | 0.2806 | 0.7872 |
| 4n75A | 0.6768 | 0.5265 | 0.6875 |
| 4njnA | 0.7974 | 0.8275 | 0.7897 |
| 4nppA | 0.4635 | 0.3358 | 0.3743 |
| 4ntjA | 0.5233 | 0.5783 | 0.5736 |
| 4nykA | 0.5547 | 0.2433 | 0.5971 |
| 4o6mA | 0.4872 | 0.4464 | 0.5428 |
| 4o6yA | 0.7727 | 0.7953 | 0.8229 |
| 4o9pA | 0.5137 | 0.5024 | 0.5336 |
| 4o9pB | 0.5975 | 0.3882 | 0.8238 |
| 4o9uB | 0.4712 | 0.3253 | 0.5062 |
| 4od4A | 0.8394 | 0.848 | 0.8164 |
| 4ogqC | 0.2345 | 0.2838 | 0.4254 |
| 4oh3A | 0.636 | 0.7527 | 0.755 |
| 4oo9A | 0.4832 | 0.4754 | 0.5402 |
| 4or2A | 0.5175 | 0.6078 | 0.6501 |
| 4p6vB | 0.6718 | 0.7323 | 0.6958 |
| 4p6vC | 0.7186 | 0.592 | 0.7464 |
| 4p6vD | 0.5634 | 0.7646 | 0.5671 |
| 4p6vE | 0.7958 | 0.758 | 0.8022 |
| 4p6vF | 0.6329 | 0.4363 | 0.7147 |
| 4p79A | 0.7562 | 0.822 | 0.8413 |
| 4pgrA | 0.6699 | 0.6633 | 0.6113 |
| 4phzA | 0.3107 | 0.2097 | 0.3443 |
| 4pirA | 0.3036 | 0.3284 | 0.3629 |
| 4px7A | 0.4935 | 0.4682 | 0.4712 |
| 4q2eA | 0.5953 | 0.6021 | 0.6615 |
| 4qncA | 0.8424 | 0.7278 | 0.7784 |
| 4qndA | 0.6597 | 0.6323 | 0.6647 |
| 4qtnA | 0.6382 | 0.7773 | 0.7575 |
| 4quvA | 0.6427 | 0.6513 | 0.7989 |
| 4r1iA | 0.4466 | 0.5739 | 0.5274 |
| 4rdqA | 0.3845 | 0.3497 | 0.4609 |
| 4rfsS | 0.7466 | 0.6987 | 0.7336 |
| 4ri2A | 0.3579 | 0.4477 | 0.3845 |
| 4rjwA | 0.5297 | 0.4822 | 0.6761 |
| 4rl8A | 0.7471 | 0.7557 | 0.7423 |
| 4rl9A | 0.4503 | 0.3291 | 0.6815 |
| 4rlcA | 0.6894 | 0.8185 | 0.8349 |
| 4rngA | 0.734 | 0.6903 | 0.7266 |
| 4rp8A | 0.5488 | 0.2837 | 0.5507 |
| 4ryiA | 0.7922 | 0.7285 | 0.818 |
| 4s0vA | 0.5354 | 0.3733 | 0.5723 |
| 4tkrA | 0.7265 | 0.6963 | 0.7611 |
| 4tq3A | 0.7081 | 0.6874 | 0.6792 |
| 4tquM | 0.4755 | 0.7068 | 0.647 |
| 4tquN | 0.3466 | 0.6698 | 0.4526 |
| 4twkA | 0.31 | 0.3344 | 0.4074 |
| 4u15A | 0.5867 | 0.5872 | 0.6394 |
| 4u4tA | 0.7649 | 0.8076 | 0.7788 |
| 4u9lA | 0.6021 | 0.651 | 0.5423 |
| 4uc1A | 0.8659 | 0.7907 | 0.8175 |
| 4us3A | 0.8744 | 0.7706 | 0.8312 |
| 4v1fA | 0.6338 | 0.7732 | 0.7072 |
| 4wd7A | 0.7395 | 0.658 | 0.7717 |
| 4wgvA | 0.7245 | 0.7758 | 0.7406 |
| 4wmzA | 0.7877 | 0.7337 | 0.7567 |
| 4x5mA | 0.6624 | 0.6395 | 0.6528 |
| 4xk83 | 0.4518 | 0.4564 | 0.5167 |
| 4xnkA | 0.2699 | 0.5554 | 0.6498 |
| 4xnvA | 0.636 | 0.6519 | 0.7164 |
| 4xu4A | 0.7309 | 0.3846 | 0.7649 |
| 4xxjA | 0.8275 | 0.6595 | 0.8604 |
| 4xydB | 0.5457 | 0.4872 | 0.5696 |
| 4y25A | 0.781 | 0.6107 | 0.8162 |
| 4y28G | 0.4373 | 0.3723 | 0.4254 |
| 4y28K | 0.5202 | 0.4484 | 0.5005 |
| 4y28L | 0.4904 | 0.5705 | 0.5279 |
| 4y7jA | 0.4532 | 0.463 | 0.5082 |
| 4ymkA | 0.6606 | 0.3382 | 0.679 |
| 4ymsC | 0.4637 | 0.7708 | 0.4671 |
| 4ytpC | 0.3568 | 0.537 | 0.5551 |
| 4ytpD | 0.5864 | 0.542 | 0.5956 |
| 4z34A | 0.5615 | 0.6433 | 0.5812 |
| 4z3nA | 0.757 | 0.7924 | 0.7656 |
| 4z7fA | 0.7618 | 0.7758 | 0.7437 |
| 4zp0A | 0.8243 | 0.8488 | 0.8026 |
| 4zr0A | 0.5228 | 0.3276 | 0.6786 |
| 4zr1A | 0.4574 | 0.387 | 0.3313 |
| 4zw9A | 0.8757 | 0.5697 | 0.8479 |
| 5a1sA | 0.4329 | 0.3952 | 0.5188 |
| 5a40A | 0.6244 | 0.7976 | 0.647 |
| 5a63C | 0.7314 | 0.6612 | 0.8115 |
| 5a63D | 0.5658 | 0.5393 | 0.5607 |
| 5a6eB | 0.8291 | 0.7337 | 0.8391 |
| 5abbZ | 0.4605 | 0.411 | 0.432 |
| 5araT | 0.2078 | 0.3324 | 0.2182 |
| 5araW | 0.4414 | 0.5197 | 0.5117 |
| 5awwG | 0.6435 | 0.6673 | 0.6333 |
| 5awwY | 0.5922 | 0.4242 | 0.7488 |
| 5awzA | 0.7942 | 0.8204 | 0.8365 |
| 5aymA | 0.8081 | 0.7657 | 0.8184 |
| 5azbA | 0.6844 | 0.7149 | 0.6833 |
| 5bwkE | 0.4651 | 0.4785 | 0.7487 |
| 5c6oA | 0.8266 | 0.8493 | 0.8112 |
| 5c8jI | 0.4038 | 0.3291 | 0.3631 |
| 5cfbA | 0.3658 | 0.3602 | 0.4876 |
| 5ctgA | 0.798 | 0.7663 | 0.8071 |
| 5d0yA | 0.735 | 0.7649 | 0.7581 |
| 5dirA | 0.7698 | 0.594 | 0.7409 |
| 5doqA | 0.8274 | 0.6941 | 0.8479 |
| 5doqB | 0.7743 | 0.4748 | 0.7638 |
| 5ee7A | 0.5575 | 0.4956 | 0.5837 |
| 5ek0A | 0.4517 | 0.3798 | 0.4446 |
| 5ekeA | 0.5233 | 0.5223 | 0.5398 |
| 5eulE | 0.5052 | 0.4719 | 0.5521 |
| 5ezmA | 0.445 | 0.4381 | 0.6955 |
| 5f1cA | 0.2454 | 0.2858 | 0.5266 |
| 5fn2B | 0.3184 | 0.2641 | 0.4329 |
| 5gaeh | 0.4653 | 0.3615 | 0.4305 |
| 5gaqA | 0.1761 | 0.1734 | 0.2144 |
| 5garO | 0.5138 | 0.6123 | 0.5771 |
| 5hk1A | 0.6744 | 0.4621 | 0.7146 |
| 5i1mV | 0.3702 | 0.3898 | 0.4243 |
| 5i20A | 0.7517 | 0.7972 | 0.7298 |
| 5i32A | 0.6282 | 0.4813 | 0.8081 |
| 5i6cA | 0.5711 | 0.3472 | 0.7173 |
| 5i6zA | 0.8654 | 0.821 | 0.8453 |
| 5id3A | 0.2839 | 0.2051 | 0.2993 |
| 5iofA | 0.7028 | 0.754 | 0.8027 |
| 5irxA | 0.2581 | 0.203 | 0.3432 |
| 5ivaA | 0.287 | 0.2933 | 0.5348 |
| 5iwsA | 0.4699 | 0.486 | 0.6503 |
| 5ixmB | 0.453 | 0.7179 | 0.7388 |
| 5jagA | 0.8189 | 0.7728 | 0.7934 |
|  |  |  |  |
| Mean | 0.546479804 | 0.521108627 | 0.586360588 |
| Fold | 294 | 255 | 342 |
| p-Value | 6.54197E-06 |  | 5.10928E-27 |
