# Supplementary material for: Hybridized distance- and contact-based hierarchical structure modeling for folding soluble and membrane proteins
Source: PLoS Comput Biol. 2021 Feb 23;17(2):e1008753. doi: 10.1371/journal.pcbi.1008753 (PMC7935296; doi:10.1371/journal.pcbi.1008753)
Supplement: S10 Table — (DOCX) [file pcbi.1008753.s010.docx]

| **S10 Table.** Target-by-target stagewise reconstruction performance on EVfold dataset for true C_β_–C_β_ contact maps at 8, 10, and 12Å thresholds. | | | | | | | | | | | | | | | |
| --- | --- | --- | --- | --- | --- | --- | --- | --- | --- | --- | --- | --- | --- | --- | --- |
| Targets | 8 Å | | | | | 10 Å | | | | | 12 Å | | | | |
|  | stage 1 | stage 2 | stage 3 | Δ_1_ (stage 2-stage 1) | Δ_2_ (stage 3-stage 2) | stage 1 | stage 2 | stage 3 | Δ_1_ (stage 2-stage 1) | Δ_2_ (stage 3-stage 2) | stage 1 | stage 2 | stage 3 | Δ_1_ (stage 2-stage 1) | Δ_2_ (stage 3-stage 2) |
| 1bkrA | 0.749 | 0.8652 | 0.9031 | 0.1162 | 0.0379 | 0.6087 | 0.8781 | 0.9334 | 0.2694 | 0.0553 | 0.4376 | 0.893 | 0.9541 | 0.4554 | 0.0611 |
| 1e6kA | 0.6059 | 0.7923 | 0.8735 | 0.1864 | 0.0812 | 0.5139 | 0.843 | 0.911 | 0.3291 | 0.068 | 0.4136 | 0.8697 | 0.9179 | 0.4561 | 0.0482 |
| 1f21A | 0.6504 | 0.8178 | 0.8769 | 0.1674 | 0.0591 | 0.5361 | 0.8644 | 0.9214 | 0.3283 | 0.057 | 0.4223 | 0.8832 | 0.9132 | 0.4609 | 0.03 |
| 1g2eA | 0.4311 | 0.6716 | 0.7697 | 0.2405 | 0.0981 | 0.3934 | 0.7598 | 0.8251 | 0.3664 | 0.0653 | 0.3003 | 0.7803 | 0.8692 | 0.48 | 0.0889 |
| 1hzxA | 0.7519 | 0.8705 | 0.9126 | 0.1186 | 0.0421 | 0.6074 | 0.9135 | 0.951 | 0.3061 | 0.0375 | 0.4202 | 0.9311 | 0.953 | 0.5109 | 0.0219 |
| 1oddA | 0.5953 | 0.6706 | 0.7672 | 0.0753 | 0.0966 | 0.4254 | 0.7456 | 0.87 | 0.3202 | 0.1244 | 0.3529 | 0.7378 | 0.8813 | 0.3849 | 0.1435 |
| 1r9hA | 0.4405 | 0.7418 | 0.8207 | 0.3013 | 0.0789 | 0.4371 | 0.8212 | 0.8829 | 0.3841 | 0.0617 | 0.3293 | 0.8461 | 0.8996 | 0.5168 | 0.0535 |
| 1rqmA | 0.5908 | 0.7974 | 0.8402 | 0.2066 | 0.0428 | 0.5443 | 0.8976 | 0.9218 | 0.3533 | 0.0242 | 0.4139 | 0.8937 | 0.9245 | 0.4798 | 0.0308 |
| 1wvnA | 0.4507 | 0.7116 | 0.7808 | 0.2609 | 0.0692 | 0.4141 | 0.7621 | 0.8083 | 0.348 | 0.0462 | 0.3156 | 0.7354 | 0.7846 | 0.4198 | 0.0492 |
| 2hdaA | 0.2412 | 0.5251 | 0.6091 | 0.2839 | 0.084 | 0.2575 | 0.6491 | 0.7728 | 0.3916 | 0.1237 | 0.2576 | 0.6409 | 0.7748 | 0.3833 | 0.1339 |
| 2it6A | 0.5176 | 0.786 | 0.8619 | 0.2684 | 0.0759 | 0.4344 | 0.8228 | 0.9147 | 0.3884 | 0.0919 | 0.3643 | 0.8782 | 0.9253 | 0.5139 | 0.0471 |
| 2o72A | 0.3793 | 0.71 | 0.8067 | 0.3307 | 0.0967 | 0.3709 | 0.7893 | 0.877 | 0.4184 | 0.0877 | 0.3059 | 0.8034 | 0.9031 | 0.4975 | 0.0997 |
| 3tgiE | 0.6413 | 0.8794 | 0.9375 | 0.2381 | 0.0581 | 0.5496 | 0.9196 | 0.9564 | 0.37 | 0.0368 | 0.4423 | 0.9253 | 0.9539 | 0.483 | 0.0286 |
| 5p21A | 0.6113 | 0.8791 | 0.9312 | 0.2678 | 0.0521 | 0.5602 | 0.8956 | 0.9365 | 0.3354 | 0.0409 | 0.4344 | 0.9108 | 0.9558 | 0.4764 | 0.045 |
| 5ptiA | 0.4365 | 0.6378 | 0.7908 | 0.2013 | 0.153 | 0.4046 | 0.7334 | 0.8538 | 0.3288 | 0.1204 | 0.3507 | 0.7283 | 0.8467 | 0.3776 | 0.1184 |
|  |  |  |  |  |  |  |  |  |  |  |  |  |  |  |  |
| Mean | 0.53952 | 0.75708 | 0.832126667 | 0.21756 | 0.075046667 | 0.470506667 | 0.819673333 | 0.889073333 | 0.349166667 | 0.0694 | 0.370726667 | 0.83048 | 0.897133333 | 0.459753333 | 0.066653333 |
