# Supplementary material for: Hybridized distance- and contact-based hierarchical structure modeling for folding soluble and membrane proteins
Source: PLoS Comput Biol. 2021 Feb 23;17(2):e1008753. doi: 10.1371/journal.pcbi.1008753 (PMC7935296; doi:10.1371/journal.pcbi.1008753)
Supplement: S11 Table — (DOCX) [file pcbi.1008753.s011.docx]

| **S11 Table.** Target-by-target stagewise reconstruction performance on EVfold dataset for true C_α_–C_α_ contact maps at 8, 10, and 12Å thresholds. | | | | | | | | | | | | | | | |
| --- | --- | --- | --- | --- | --- | --- | --- | --- | --- | --- | --- | --- | --- | --- | --- |
| Targets | 8 Å | | | | | 10 Å | | | | | 12 Å | | | | |
|  | stage 1 | stage 2 | stage 3 | Δ_1_ (stage 2-stage 1) | Δ_2_ (stage 3-stage 2) | stage 1 | stage 2 | stage 3 | Δ_1_ (stage 2-stage 1) | Δ_2_ (stage 3-stage 2) | stage 1 | stage 2 | stage 3 | Δ_1_ (stage 2-stage 1) | Δ_2_ (stage 3-stage 2) |
| 1bkrA | 0.6891 | 0.8324 | 0.9021 | 0.1433 | 0.0697 | 0.6525 | 0.927 | 0.9645 | 0.2745 | 0.0375 | 0.4458 | 0.9571 | 0.9736 | 0.5113 | 0.0165 |
| 1e6kA | 0.3945 | 0.7814 | 0.8244 | 0.3869 | 0.043 | 0.5539 | 0.8892 | 0.9145 | 0.3353 | 0.0253 | 0.4318 | 0.9377 | 0.9233 | 0.5059 | -0.0144 |
| 1f21A | 0.6073 | 0.7883 | 0.8351 | 0.181 | 0.0468 | 0.5736 | 0.8887 | 0.9191 | 0.3151 | 0.0304 | 0.4469 | 0.9125 | 0.9603 | 0.4656 | 0.0478 |
| 1g2eA | 0.4059 | 0.6749 | 0.7443 | 0.269 | 0.0694 | 0.4578 | 0.8586 | 0.8554 | 0.4008 | -0.0032 | 0.3396 | 0.8229 | 0.8927 | 0.4833 | 0.0698 |
| 1hzxA | 0.7088 | 0.8073 | 0.8813 | 0.0985 | 0.074 | 0.6171 | 0.925 | 0.9634 | 0.3079 | 0.0384 | 0.4499 | 0.9662 | 0.9764 | 0.5163 | 0.0102 |
| 1oddA | 0.5462 | 0.6849 | 0.8033 | 0.1387 | 0.1184 | 0.4965 | 0.8008 | 0.8896 | 0.3043 | 0.0888 | 0.3099 | 0.8182 | 0.9052 | 0.5083 | 0.087 |
| 1r9hA | 0.3951 | 0.296 | 0.3249 | -0.0991 | 0.0289 | 0.4843 | 0.8394 | 0.9155 | 0.3551 | 0.0761 | 0.3517 | 0.8582 | 0.94 | 0.5065 | 0.0818 |
| 1rqmA | 0.4757 | 0.734 | 0.8283 | 0.2583 | 0.0943 | 0.5593 | 0.9123 | 0.9501 | 0.353 | 0.0378 | 0.4023 | 0.9455 | 0.9539 | 0.5432 | 0.0084 |
| 1wvnA | 0.3695 | 0.5584 | 0.7416 | 0.1889 | 0.1832 | 0.4363 | 0.7911 | 0.7527 | 0.3548 | -0.0384 | 0.323 | 0.8093 | 0.8395 | 0.4863 | 0.0302 |
| 2hdaA | 0.1846 | 0.2494 | 0.4379 | 0.0648 | 0.1885 | 0.2997 | 0.6601 | 0.7487 | 0.3604 | 0.0886 | 0.2236 | 0.6655 | 0.7685 | 0.4419 | 0.103 |
| 2it6A | 0.5121 | 0.6736 | 0.7843 | 0.1615 | 0.1107 | 0.4454 | 0.8744 | 0.9395 | 0.429 | 0.0651 | 0.3685 | 0.9095 | 0.9538 | 0.541 | 0.0443 |
| 2o72A | 0.3943 | 0.6761 | 0.7665 | 0.2818 | 0.0904 | 0.2303 | 0.9003 | 0.9222 | 0.67 | 0.0219 | 0.2236 | 0.8865 | 0.915 | 0.6629 | 0.0285 |
| 3tgiE | 0.6393 | 0.8758 | 0.9341 | 0.2365 | 0.0583 | 0.5616 | 0.9521 | 0.9718 | 0.3905 | 0.0197 | 0.2473 | 0.9666 | 0.9757 | 0.7193 | 0.0091 |
| 5p21A | 0.5273 | 0.7845 | 0.8533 | 0.2572 | 0.0688 | 0.5523 | 0.9362 | 0.9502 | 0.3839 | 0.014 | 0.4462 | 0.9357 | 0.97 | 0.4895 | 0.0343 |
| 5ptiA | 0.4433 | 0.6765 | 0.8368 | 0.2332 | 0.1603 | 0.4022 | 0.7791 | 0.8629 | 0.3769 | 0.0838 | 0.3544 | 0.7061 | 0.8131 | 0.3517 | 0.107 |
|  |  |  |  |  |  |  |  |  |  |  |  |  |  |  |  |
| Mean | 0.4862 | 0.6729 | 0.766546667 | 0.1867 | 0.093646667 | 0.488186667 | 0.862286667 | 0.90134 | 0.3741 | 0.039053333 | 0.357633333 | 0.873166667 | 0.9174 | 0.515533333 | 0.044233333 |
