# Supplementary material for: Hybridized distance- and contact-based hierarchical structure modeling for folding soluble and membrane proteins
Source: PLoS Comput Biol. 2021 Feb 23;17(2):e1008753. doi: 10.1371/journal.pcbi.1008753 (PMC7935296; doi:10.1371/journal.pcbi.1008753)
Supplement: S12 Table — (DOCX) [file pcbi.1008753.s012.docx]

| **S12 Table.** Target-by-target stagewise recovery of secondary structure topology on EVfold dataset for true C_β_–C_β_ contact maps at 8, 10, and 12Å thresholds. | | | | | | | | | | | | | | | | | | |
| --- | --- | --- | --- | --- | --- | --- | --- | --- | --- | --- | --- | --- | --- | --- | --- | --- | --- | --- |
| Target | 8 Å | | | | | | 10 Å | | | | | | 12 Å | | | | | |
|  | Stage 1 | | Stage 2 | | Stage 3 | | Stage 1 | | Stage 2 | | Stage 3 | | Stage 1 | | Stage 2 | | Stage 3 | |
|  | Q_H_ | Q_E_ | Q_H_ | Q_E_ | Q_H_ | Q_E_ | Q_H_ | Q_E_ | Q_H_ | Q_E_ | Q_H_ | Q_E_ | Q_H_ | Q_E_ | Q_H_ | Q_E_ | Q_H_ | Q_E_ |
| 1bkrA | 37.14285714 |  | 74.28571429 |  | 98.57142857 |  | 22.85714286 |  | 72.85714286 |  | 98.57142857 |  | 38.57142857 |  | 67.14285714 |  | 100 |  |
| 1e6kA | 37.25490196 | 0 | 70.58823529 | 20 | 92.15686275 | 85 | 17.64705882 | 0 | 23.52941176 | 0 | 96.07843137 | 85 | 9.803921569 | 0 | 45.09803922 | 10 | 100 | 55 |
| 1f21A | 29.31034483 | 4.166666667 | 63.79310345 | 27.08333333 | 98.27586207 | 60.41666667 | 18.96551724 | 0 | 41.37931034 | 31.25 | 96.55172414 | 58.33333333 | 36.20689655 | 2.083333333 | 24.13793103 | 18.75 | 96.55172414 | 64.58333333 |
| 1g2eA | 14.28571429 | 0 | 80.95238095 | 0 | 100 | 36 | 0 | 0 | 71.42857143 | 16 | 100 | 24 | 23.80952381 | 8 | 47.61904762 | 0 | 100 | 44 |
| 1hzxA | 1.104972376 | 0 | 60.22099448 | 0 | 86.1878453 | 25 | 14.36464088 | 0 | 49.17127072 | 37.5 | 86.74033149 | 12.5 | 7.73480663 | 12.5 | 52.48618785 | 12.5 | 88.95027624 | 12.5 |
| 1oddA | 9.375 | 0 | 59.375 | 0 | 100 | 42.85714286 | 9.375 | 0 | 53.125 | 0 | 96.875 | 71.42857143 | 15.625 | 0 | 84.375 | 0 | 100 | 57.14285714 |
| 1r9hA | 0 | 0 | 42.85714286 | 0 | 100 | 77.77777778 | 0 | 0 | 35.71428571 | 5.555555556 | 78.57142857 | 80.55555556 | 0 | 11.11111111 | 71.42857143 | 38.88888889 | 71.42857143 | 72.22222222 |
| 1rqmA | 10.25641026 | 0 | 51.28205128 | 24 | 89.74358974 | 68 | 30.76923077 | 0 | 58.97435897 | 24 | 87.17948718 | 80 | 0 | 0 | 61.53846154 | 20 | 87.17948718 | 80 |
| 1wvnA | 19.35483871 | 0 | 74.19354839 | 17.64705882 | 100 | 70.58823529 | 29.03225806 | 5.882352941 | 87.09677419 | 23.52941176 | 100 | 82.35294118 | 0 | 0 | 41.93548387 | 23.52941176 | 100 | 82.35294118 |
| 2hdaA |  | 5.263157895 |  | 0 |  | 47.36842105 |  | 5.263157895 |  | 21.05263158 |  | 68.42105263 |  | 0 |  | 26.31578947 |  | 89.47368421 |
| 2it6A | 11.11111111 | 0 | 66.66666667 | 8.823529412 | 100 | 58.82352941 | 0 | 11.76470588 | 48.14814815 | 5.882352941 | 100 | 58.82352941 | 0 | 2.941176471 | 37.03703704 | 29.41176471 | 96.2962963 | 70.58823529 |
| 2o72A |  | 2.127659574 |  | 27.65957447 |  | 87.23404255 |  | 8.510638298 |  | 27.65957447 |  | 61.70212766 |  | 0 |  | 12.76595745 |  | 80.85106383 |
| 3tgiE | 0 | 2.631578947 | 0 | 13.15789474 | 57.14285714 | 71.05263158 | 42.85714286 | 2.631578947 | 0 | 13.15789474 | 100 | 77.63157895 | 0 | 1.315789474 | 42.85714286 | 26.31578947 | 57.14285714 | 81.57894737 |
| 5p21A | 0 | 0 | 75.80645161 | 5.128205128 | 98.38709677 | 74.35897436 | 12.90322581 | 0 | 50 | 15.38461538 | 98.38709677 | 71.79487179 | 17.74193548 | 0 | 53.22580645 | 28.20512821 | 98.38709677 | 87.17948718 |
| 5ptiA | 0 | 0 | 50 | 40 | 100 | 60 | 0 | 0 | 100 | 26.66666667 | 100 | 86.66666667 | 0 | 20 | 37.5 | 40 | 100 | 100 |
|  |  |  |  |  |  |  |  |  |  |  |  |  |  |  |  |  |  |  |
| Mean | 13.01508851 | 1.013504506 | 59.23240687 | 13.10711399 | 93.8819648 | 61.74838725 | 15.29009364 | 2.432316712 | 53.18648263 | 17.68847879 | 95.30422524 | 65.65787347 | 11.49950097 | 4.139386456 | 51.26012046 | 20.47733785 | 91.99510071 | 69.8194837 |
