# Supplementary material for: Hybridized distance- and contact-based hierarchical structure modeling for folding soluble and membrane proteins
Source: PLoS Comput Biol. 2021 Feb 23;17(2):e1008753. doi: 10.1371/journal.pcbi.1008753 (PMC7935296; doi:10.1371/journal.pcbi.1008753)
Supplement: S13 Table — (DOCX) [file pcbi.1008753.s013.docx]

| **S13 Table.** Target-by-target stagewise recovery of secondary structure topology on EVfold dataset for true C_α_–C_α_ contact maps at 8, 10, and 12Å thresholds. | | | | | | | | | | | | | | | | | | |
| --- | --- | --- | --- | --- | --- | --- | --- | --- | --- | --- | --- | --- | --- | --- | --- | --- | --- | --- |
| Target | 8 Å | | | | | | 10 Å | | | | | | 12 Å | | | | | |
|  | Stage 1 | | Stage 2 | | Stage 3 | | Stage 1 | | Stage 2 | | Stage 3 | | Stage 1 | | Stage 2 | | Stage 3 | |
|  | Q_H_ | Q_E_ | Q_H_ | Q_E_ | Q_H_ | Q_E_ | Q_H_ | Q_E_ | Q_H_ | Q_E_ | Q_H_ | Q_E_ | Q_H_ | Q_E_ | Q_H_ | Q_E_ | Q_H_ | Q_E_ |
| 1bkrA | 18.57142857 |  | 72.85714286 |  | 95.71428571 |  | 21.42857143 |  | 77.14285714 |  | 97.14285714 |  | 28.57142857 |  | 75.71428571 |  | 97.14285714 |  |
| 1e6kA | 5.882352941 | 0 | 68.62745098 | 0 | 94.11764706 | 80 | 5.882352941 | 0 | 68.62745098 | 30 | 96.07843137 | 45 | 1.960784314 | 0 | 80.39215686 | 20 | 96.07843137 | 40 |
| 1f21A | 0 | 0 | 63.79310345 | 16.66666667 | 94.82758621 | 68.75 | 32.75862069 | 0 | 86.20689655 | 47.91666667 | 96.55172414 | 62.5 | 13.79310345 | 0 | 86.20689655 | 29.16666667 | 100 | 75 |
| 1g2eA | 14.28571429 | 0 | 71.42857143 | 0 | 95.23809524 | 8 | 0 | 4 | 76.19047619 | 0 | 100 | 24 | 0 | 0 | 90.47619048 | 0 | 100 | 16 |
| 1hzxA | 17.12707182 | 0 | 62.98342541 | 0 | 83.97790055 | 0 | 12.15469613 | 25 | 70.71823204 | 0 | 87.84530387 | 0 | 13.8121547 | 0 | 78.45303867 | 0 | 87.29281768 | 12.5 |
| 1oddA | 15.625 | 0 | 65.625 | 0 | 96.875 | 100 | 0 | 0 | 93.75 | 0 | 96.875 | 71.42857143 | 0 | 0 | 90.625 | 28.57142857 | 100 | 71.42857143 |
| 1r9hA | 0 | 0 | 28.57142857 | 0 | 64.28571429 | 16.66666667 | 28.57142857 | 0 | 57.14285714 | 11.11111111 | 78.57142857 | 91.66666667 | 0 | 2.777777778 | 57.14285714 | 16.66666667 | 78.57142857 | 80.55555556 |
| 1rqmA | 0 | 0 | 43.58974359 | 36 | 89.74358974 | 88 | 28.20512821 | 8 | 66.66666667 | 24 | 92.30769231 | 88 | 30.76923077 | 0 | 71.79487179 | 32 | 89.74358974 | 72 |
| 1wvnA | 22.58064516 | 0 | 70.96774194 | 0 | 100 | 47.05882353 | 0 | 0 | 87.09677419 | 0 | 100 | 70.58823529 | 0 | 11.76470588 | 77.41935484 | 23.52941176 | 100 | 82.35294118 |
| 2hdaA |  | 0 |  | 0 |  | 10.52631579 |  | 0 |  | 21.05263158 |  | 63.15789474 |  | 0 |  | 0 |  | 89.47368421 |
| 2it6A | 11.11111111 | 0 | 70.37037037 | 0 | 85.18518519 | 23.52941176 | 0 | 5.882352941 | 81.48148148 | 23.52941176 | 96.2962963 | 58.82352941 | 0 | 5.882352941 | 48.14814815 | 0 | 100 | 58.82352941 |
| 2o72A |  | 0 |  | 17.0212766 |  | 74.46808511 |  | 4.255319149 |  | 12.76595745 |  | 80.85106383 |  | 0 |  | 31.91489362 |  | 65.95744681 |
| 3tgiE | 0 | 0 | 42.85714286 | 23.68421053 | 100 | 67.10526316 | 0 | 5.263157895 | 57.14285714 | 32.89473684 | 100 | 81.57894737 | 0 | 1.315789474 | 42.85714286 | 48.68421053 | 100 | 76.31578947 |
| 5p21A | 11.29032258 | 0 | 69.35483871 | 30.76923077 | 100 | 66.66666667 | 14.51612903 | 2.564102564 | 85.48387097 | 35.8974359 | 98.38709677 | 58.97435897 | 17.74193548 | 0 | 79.03225806 | 38.46153846 | 100 | 87.17948718 |
| 5ptiA | 12.5 | 0 | 87.5 | 26.66666667 | 100 | 86.66666667 | 0 | 0 | 87.5 | 26.66666667 | 100 | 86.66666667 | 0 | 0 | 62.5 | 13.33333333 | 100 | 86.66666667 |
|  |  |  |  |  |  |  |  |  |  |  |  |  |  |  |  |  |  |  |
| Mean | 9.921049729 | 0 | 62.9635354 | 10.77200366 | 92.30500031 | 52.67413567 | 11.03976362 | 3.926066611 | 76.55003235 | 18.988187 | 95.38891004 | 63.08828103 | 8.203741329 | 1.552901863 | 72.36632316 | 20.1662964 | 96.06377881 | 65.30383371 |
