# Supplementary material for: Hybridized distance- and contact-based hierarchical structure modeling for folding soluble and membrane proteins
Source: PLoS Comput Biol. 2021 Feb 23;17(2):e1008753. doi: 10.1371/journal.pcbi.1008753 (PMC7935296; doi:10.1371/journal.pcbi.1008753)
Supplement: S14 Table — (DOCX) [file pcbi.1008753.s014.docx]

| **S14 Table.** *Ab initio* folding performance of DConStruct on EVfold dataset using top hybrid interaction maps with tri-level thresholding at increasing xL values (x = 2, 4, 8, 16). | | | | |
| --- | --- | --- | --- | --- |
| Target | 2L | 4L | 8L | 16L |
| 1bkrA | 0.8383 | 0.8267 | 0.8257 | 0.7377 |
| 1e6kA | 0.8332 | 0.8516 | 0.8972 | 0.784 |
| 1f21A | 0.7557 | 0.7753 | 0.8162 | 0.7386 |
| 1g2eA | 0.5946 | 0.7795 | 0.7998 | 0.6301 |
| 1hzxA | 0.6165 | 0.715 | 0.7326 | 0.6934 |
| 1oddA | 0.194 | 0.2153 | 0.2592 | 0.2625 |
| 1r9hA | 0.544 | 0.7663 | 0.7929 | 0.6209 |
| 1rqmA | 0.7086 | 0.7168 | 0.7427 | 0.6888 |
| 1wvnA | 0.719 | 0.807 | 0.8566 | 0.5849 |
| 2hdaA | 0.2272 | 0.2188 | 0.2816 | 0.1883 |
| 2it6A | 0.1248 | 0.2079 | 0.2617 | 0.2008 |
| 2o72A | 0.1724 | 0.1568 | 0.1749 | 0.1777 |
| 3tgiE | 0.729 | 0.7821 | 0.8513 | 0.8407 |
| 5p21A | 0.781 | 0.7736 | 0.8141 | 0.7656 |
| 5ptiA | 0.6135 | 0.7171 | 0.7072 | 0.5867 |
|  |  |  |  |  |
| Mean | 0.563453333 | 0.620653333 | 0.654246667 | 0.566713333 |
